# New insights into host adaptation to swine respiratory disease revealed by genetic differentiation and RNA sequencing analyses

**DOI:** 10.1111/eva.12737

**Published:** 2018-12-03

**Authors:** Mingpeng Zhang, Tao Huang, Xiaochang Huang, Xinkai Tong, Jiaqi Chen, Bin Yang, Shijun Xiao, Yuanmei Guo, Huashui Ai, Lusheng Huang

**Affiliations:** ^1^ State Key Laboratory for Swine Genetic Improvement and Production Technology Jiangxi Agricultural University Nanchang China

**Keywords:** adaptation to chronic disease, genetic differentiation analyses, pig, RNA sequencing, swine respiratory disease

## Abstract

Swine respiratory disease (SRD) causes massive economic losses in the swine industry and is difficult to control and eradicate on pig farms. Here, we employed population genetics and transcriptomics approaches to decipher the molecular mechanism of host adaptation to swine respiratory disease. We recorded two SRD‐related traits, the enzootic pneumonia‐like (EPL) score and lung lesion (LL) levels, and performed four body weight measurements, at ages of 150, 180, 240, and 300 days, in a Chinese Bamaxiang pig herd (*n* = 314) raised under consistent indoor rearing conditions. We divided these animals into disease‐resistant and disease‐susceptible groups based on the most likely effects of both SRD‐related traits on their weight gain, and performed genetic differentiation analyses in these two groups. Significant loci showing the top 1% of genetic differentiation values, exceeding the threshold of *p* = 0.005 set based on 1,000‐times permutation tests, were defined as candidate regions related to host resistance or susceptibility to SRD. We identified 107 candidate genes within these regions, which are mainly involved in the biological processes of immune response, fatty acid metabolism, lipid metabolism, and growth factor signaling pathways. Among these candidate genes, *TRAF6*, *CD44*, *CD22*, *TGFB1*, *CYP2B6,* and *SNRPA* were highlighted due to their central regulatory roles in host immune response or fat metabolism and their differential expression between healthy lung tissues and lung lesions. These findings advance our understanding of the molecular mechanisms of host resistance or susceptibility to respiratory disease in pigs and are of significance for the breeding pigs resistant to respiratory disease in the swine industry.

## 1 INTRODUCTION

Swine respiratory disease (SRD), also referred as the swine/porcine respiratory disease complex (SRDC/PRDC), is prevalent in modern intensive pig farms worldwide and can cause poor porcine growth performance, in turn leading to serious economic losses to the swine industry (Opriessnig, Gimenez‐Lirola, & Halbur, 2011). This disease is arguably the most important health concern for swine producers at present (Brockmeier, Halbur, & Thacker, 2002). Multiple infectious agents are usually involved in swine respiratory disease (Brockmeier et al., 2002; Opriessnig et al., 2011). These agents can be divided into primary bacterial pathogens, such as *Mycoplasma hyopneumoniae *(Thacker, 2004) and *Bordetella bronchiseptica* (Brockmeier, 2004); primary viral pathogens, such as porcine circovirus type 2 (Kim, Chung, & Chae, 2003) and porcine reproductive and respiratory syndrome virus (Thacker, Halbur, Ross, Thanawongnuwech, & Thacker, 1999); and secondary or opportunistic pathogens, such as *Pasteurella multocida* (Ciprian et al., 1988). In addition, environmental factors and management conditions, such as a high animal stocking density and negligent housing maintenance, could increase risk to affect swine respiratory disease (Maes et al., 2008; Stark, 2000). On pig farms, swine respiratory disease caused by viral pathogens can often be effectively controlled by timely vaccination. However, swine respiratory disease caused by microbial pathogens, especially *M. hyopneumoniae*, is difficult to prevent and is prone to developing into a chronic, endemic disease that recessively exists in pig populations over the long term, resulting in reduction of the porcine growth rate and decreasing feed efficiency (Brockmeier et al., 2002). Under natural farming conditions, pigs are always subject to an adverse disease environment for long‐term recessive swine respiratory disease caused by bacterial agents, most likely by *M. hyopneumoniae*. It was shown that 93.4% of pig lungs exhibited pneumonic lesions among 4,508 pigs at slaughter, and 98.1% of enzootic pneumonia (EP)‐affected lungs showed lesions similar to pneumonic lesions caused by *M. hyopneumoniae* (Wallgren, Beskow, Fellström, & Renström, 1994), which demonstrated that the pigs had been subjected to an adverse SRD environment.

Great efforts have been devoted to controlling the prevalence of swine respiratory disease, including improving the feeding environment and management, implementing vaccination schedules, and creating pathogen eradication schemes (Bargen, 2004; Maes, Verdonck, Deluyker, & Kruif, 1996; Tzivara, Kritas, Bourriel, Alexopoulos, & Kyriakis, 2007). On the other hand, increasing host immunity or resistance to the pathogens of swine respiratory disease would be an alternative important approach to control disease development. Recently, several pilot studies have been conducted to explore the genetic mechanisms of susceptibility/resistance to swine respiratory disease (Fang et al., 2013, 2015; Huang et al., 2017; Okamura et al., 2012). Using quantitative genetic methods, five significant and 18 suggestive quantitative trait loci (QTL) for swine respiratory disease traits and immune capacity traits were identified in a Landrace pig line selected for SRD resistance over five generations (Okamura et al., 2012). Genome‐wide association studies for an SRD trait have been performed in Chinese Erhualian pigs, and five novel loci were identified on swine chromosomes 2, 8, 12, and 14 (Huang et al., 2017). The *CXCL6*, *CXCL8*, *KIT,* and *CTBP2* genes were identified as possibly corresponding to host resistance or susceptibility to the pathogens of swine respiratory disease (Huang et al., 2017). Some other candidate genes, such as *TLR4* (Fang et al., 2013) and *CYP1A1* (Fang et al., 2016), have also been reported to be relevant to host resistance or susceptibility to swine respiratory disease. In addition, the primary immunodeficiency, Toll‐like receptor signaling, and steroid metabolism pathways might play important roles in the regulation of the inflammatory response to *M. hyopneumoniae* infection in pigs (Fang et al., 2015). Previous genetic studies have provided important clues to elucidate the biological mechanism of host resistance to the pathogens of respiratory disease in pigs, which have suggested that the genetic basis of host resistance to swine respiratory disease is complex.

Bamaxiang pigs are a well‐known Chinese indigenous breed distributed around Bama Yao Autonomous County in Guangxi Province, China. These pigs are characterized by two‐end black coat color, small body size, and good meat quality (Wang et al., 2011). Owing to their small body size, Bamaxiang pigs can serve as model animals for biomedical research (Liu, Zeng, Shang, Cen, & Wei, 2008). In the present study, Bamaxiang pigs were used to investigate host resistance or susceptibility to swine respiratory disease. We recorded body weights with different ages and two traits representing the extent to which the pigs suffered from swine respiratory disease in a herd of Bamaxiang pigs under consistent indoor farming conditions. According to the effects of both SRD traits on weight gain, which were estimated via the maximum likelihood method, the pigs were divided into disease‐susceptible and disease‐resistant groups. Then, the polygenic effects on animal adaptation to the respiratory disease were explored through genetic differentiation analyses with a whole‐genome high‐density SNP dataset. Finally, a transcriptomics study was conducted to compare the expression of candidate genes between healthy lung tissues and lung lesion tissues.

The present study attempted to employ population genetics and transcriptomics methods to elucidate the genetic mechanism of host adaption to a chronic disease in mammals. Our findings provide novel insights into the genetic architecture of host adaptation to swine respiratory disease, will be beneficial for the breeding of pigs resistant to swine respiratory disease in the swine industry, and may provide a useful reference for revealing the molecular genetic basis of host resistance to chronic refractory diseases in other mammals.

## 2 MATERIALS AND METHODS

### 2.1 Ethics statement

All procedures performed in this study involving animals were in compliance with guidelines for the care and use of experimental animals established by the Ministry of Agriculture of China. The ethics committee of Jiangxi Agricultural University specifically approved this study.

### 2.2 Experimental animals

The experimental animals investigated in the present study were bought from the core‐breeding farm of Bamaxiang pigs in Bama Yao Autonomous County, Guangxi Province. These animals consisted of 155 males and 159 females from 43 sire and 61 dam families, which were selected so that the genetic diversity of the population was as high as possible in this study. These piglets were immunized with several common vaccines according to their instructions, including those for classical swine fever, foot‐and‐mouth disease, pseudorabies, porcine reproductive and respiratory syndrome, and porcine circovirus type 2. All male pigs were castrated postweaning. At the age of approximately 2 months, the pigs were transported to Nanchang, Jiangxi Province, and were raised under consistent indoor rearing conditions on a farm in two rows of head‐to‐head pens, where each pen (approximately 10 m^2^) hosted 5–8 pigs. All animals were fed twice a day (10:00 a.m. and 16:00 p.m.) with a diet containing 16% crude protein, 3,100 kJ of digestible energy, and 0.78% lysine. Water was available ad libitum from automatic faucets. During the study period, the affected pigs with severe respiratory symptoms were treated with the medicine florfenicol following the provided instructions. All pigs were uniformly slaughtered at 300 ± 3 days at an abattoir for trait measurements.

### 2.3 Phenotypic measurements

To estimate the extent to which the pigs suffered from swine respiratory disease, we recorded two related traits. The first trait was the enzootic pneumonia‐like (EPL) score, and the other was lung lesion (LL) levels.

The EPL score was described previously (Huang et al., 2017). In brief, the respiratory status of all pigs was recorded twice a day within one hour of the feeding time. When we found a pig standing with a dry cough or lying down with obvious abdominal breathing, accompanied by fast breath and a lack of appetite, an EPL score of one was given to the individual. The sum of the EPL scores was then calculated for each individual during the rearing period from 100 to 300 days of age, which was treated as the value of the EPL score trait for that pig. In the Bamaxiang pig population, the smallest EPL score was zero, indicating that the animal did not suffer from asthma; the largest EPL score was 112, indicating that the pig suffered from the most severe asthma.

Immediately after slaughter, each pig lung was placed in a brightly lit position, and photographs of both the anterior and posterior sides were taken. Lung lesion levels were estimated based on lung photographs referring to a scoring criterion similar to that described in a previous report (Qu, Liu, Yao, & Jin, 2012). First, we evaluated the proportion of the lesion area in different parts of pig lung, including the anterior and posterior sides. Then, different weights were assigned to different lung parts; that is, weights of 10%, 10%, 25%, 10%, 10%, 25%, and 10% were assigned to the left apical, left cardiac, left diaphragmatic, right apical, right cardiac, right diaphragmatic, and accessory lobes, respectively. As all six parts of the lung except for the accessory lobe can be observed from both the anterior and posterior sides, a weight of 50% was equally assigned to the anterior and posterior sides, and a weight of 100% was assigned to the posterior side of the accessory lobe. Finally, the lung lesion level in each pig was obtained by summing the lesion area proportions of these seven lobes multiplied by their own weights (Supporting information Figure S1).

All the Bamaxiang pigs were weighed using an electronic scale, and their weights were recorded at the ages of 150, 180, 240, and 300 days.

### 2.4 SNP genotyping and quality control

Genomic DNA was extracted from the ear tissue of each animal using a standard phenol/chloroform approach. DNA quality and concentrations were determined via agarose gel electrophoresis and using a NanoDrop 1,000 spectrophotometer (Thermo Fisher, USA). Before genotyping, DNA samples were diluted to a final concentration of 50 ng/ml. All experimental pigs were genotyped using Affymetrix Axiom customized genotyping arrays containing 1,348,804 porcine SNPs (Zhang et al., 2017). Quality control was performed with Plink v1.07 (Purcell et al., 2007) with parameters of an SNP call rate>90% and a minor allele frequency (MAF) > 0.01. After the quality control step, a total of 782,364 SNPs were used for genetic differentiation analyses, among which 25,427 SNPs were located on the X chromosome.

### 2.5 Animal grouping

To perform genetic differentiation analyses, the Bamaxiang pigs were divided into disease‐susceptible and disease‐resistant subgroups based on their phenotypic data related to respiratory disease and the effects of respiratory disease on their weight gain. Before grouping the animals, we conducted independent maximum likelihood tests to estimate the reasonable effects of the EPL score and LL levels on porcine weight gain. The maximum likelihood test was described previously (Huang et al., 2017). Taking the EPL score as an example, we first determined a threshold of EPL score (*x*): If the EPL score of an individual was less than or equal to *x*, the individual was assigned to the disease‐resistant group; otherwise, it was assigned to the disease‐susceptible group. *t* test values between the two groups were calculated for body weight on all test days. Then, the likelihood statistic *L*(x) was calculated according to the following model:

(1) $$L\left(x\right)=-{\text{log}}_{10}\left(\prod_{i=1}^{4}p_{i}\left(x\right)\right)$$

where *p*(*x*) denotes the *p*‐value of the *t* test between the two groups at a given presumed *x*, and *i* ranged from 1 to 4, indicating the time point (150, 180, 240, and 300 days, respectively). The most likely classification threshold of the EPL score was determined based on the maximum likelihood statistic *L*(*x*). Then, the pigs were assigned to the disease‐susceptible group if their EPL scores were greater than the most likely classification threshold but were assigned to the disease‐resistant group if their EPL scores were less than or equal to the threshold.

The same strategy was used for animal grouping based on the effects of LL levels on porcine weight gain. Additionally, in the third grouping method, we clustered the animals in both disease‐resistant groups according to EPL scores and LL levels in the disease‐resistant group, while the animals in both disease‐susceptible groups according to EPL scores and LL levels were assigned to the disease‐susceptible group.

### 2.6 Genetic differentiation analyses

Two parameters, population genetic differentiation and the delta allele frequency, were estimated between the disease‐susceptible and disease‐resistant groups in the Bamaxiang pig population. Unbiased genetic differentiation estimates of the fixation index (F_ST_) were calculated as described by Akey, Zhang, Zhang, Jin, and Shriver (2002) using the SNP dataset. Briefly, *F*_ST_ was estimated as follows:

(2) $$F_{\text{ST}}=\frac{\text{MSP}-\text{MSG}}{\text{MSP}+(n_{c}-1)\text{MSG}}$$

where MSG and MSP denote the observed mean square errors for loci within and between populations, respectively, and *n_c_* is the average sample size across samples, which incorporates and corrects for the variance in the sample size over population.

(3) $$\text{MSG}=\frac{1}{\sum_{i=1}^{S}n_{i}-1}\sum_{i}^{S}n_{i}p_{\mathrm{Ai}}\left(1-p_{\mathrm{Ai}}\right)$$

(4) $$\text{MSP}=\frac{1}{S-1}\sum_{i}^{S}n_{i}{\left(p_{\mathrm{Ai}}-{\overset{¯}{p}}_{A}\right)}^{2}$$

(5) $$n_{c}=\frac{1}{S-1}\sum_{i=1}^{S}n_{i}-\frac{\sum_{i}n_{i}^{2}}{\sum_{i}n_{i}}$$

In the above formulae, *n_i_*denotes the sample size in the *i*th population; *p_Ai_* is the frequency of SNP allele A in the *i*th population; and $\overset{-}{p}$ *_A_* is a weighted average of *p_A_* across populations. Because the range of *F*_ST_ was originally defined as 0 to 1 (Wright, 1951), negative F_ST _values with no biological interpretation were set to 0. The mean *F*_ST_ value in each 50 kb window, with a sliding window size of 25 kb, was calculated to represent the genetic differentiation extent of a locus. Finally, the mean *F*_ST_ values of 93,425 loci in total were obtained. The top 1% of loci according to genetic differentiation values were defined as candidate regions related to host resistance or susceptibility to swine respiratory disease.

The delta allele frequency is the difference in the frequencies of an allele between the two populations. To verify the results of the population differentiation analysis, we calculated the absolute delta minor allele frequency (deltaMAF) of each SNP within the candidate loci between the disease‐susceptible and disease‐resistant subgroups, treating the disease‐susceptible subgroup as the reference population. The mean deltaMAF values were calculated in each 50 kb window, with a sliding window size of 25 kb, in the same manner as for the *F*_ST _computation.

### 2.7 Permutation test

The permutation test was performed similar to a previous report (Churchill & Doerge, 1994). Individuals in the experiment were indexed from 1 to *n*. The data were shuffled by computing a random permutation of the indices 1,..., *n* and assigning the ith phenotypic value to the individual whose index was given by the ith element of the permutation. The shuffled data were then analyzed for F_ST_ calculation according to the initial grouping state. The resulting test statistics at each analysis point are stored, and the entire procedure of shuffling and F_ST_ calculation was repeated 1,000‐times. At the end of this process, we stored the results of genetic differentiation analyses on 1,000 shuffled datasets. For each time, the 100(1–*α*) percentile was found. The mean of these 100(1–*α*) percentile values was set as the threshold of significance level α. For example, the mean of the 99.5 percentile values of 1,000‐times permutation tests was set as the threshold of *p* = 0.005.

### 2.8 Characterization of candidate genes

Candidate genes within the above candidate regions were searched in Build 10.2 of the pig reference genome via the Ensembl Genome Browser (http://www.ensembl.org/Sus_scrofa/Info/Index). The genes that were orthologous to human genes were annotated with Ensembl Biomart (https://www.ensembl.org/biomart) with filter parameters of “orgholog confidence==1” and “ortholog_one2one.” GO terms and KEGG pathways were enriched using the default options of the ClueGO plug‐in (Bindea et al., 2009) of the Cytoscape 3.5.0 platform (Shannon et al., 2003), setting human orthologous EnsemblGeneIDs as the gene list and the human Gene Ontology database as the query database.

Additionally, we input these human orthologous genes to query the STRING 10.5 database (Szklarczyk et al., 2015), searching protein–protein interaction (PPI) networks related to pig adaption to swine respiratory disease. The topological properties of PPI networks, such as betweenness, node degree, and eigenvector, were analyzed using the CentiScape v2.2 plug‐in (Scardoni, Petterlini, & Laudanna, 2009) of the Cytoscape 3.5.0 platform. The intersecting genes passing the thresholds of the three parameters were considered to be critical nodes. These nodes with high centrality are also referred to as key node genes and act as key connector proteins of the major network processes.

### 2.9 Gene expression analysis

Five infected tissue samples from the lung lesions and nine noninfected tissue samples from healthy lung parts were collected from eleven pigs in a commercial black pig population (Supporting information Table S1) under common indoor farming conditions. RNA sequencing data were obtained from these fourteen lung tissue samples; RNA‐seq reads from each sample were aligned to Build 10.2 of the pig reference genome with STAR v.2.5.3 (Dobin et al., 2013), using genomic sequence and transcript annotations derived from Release 89 of the Ensembl database. Transcripts were assembled with Cufflinks v.2.1.1 (Trapnell et al., 2010). Gene counts were calculated with FeatureCounts implemented in Subread v.1.5.2 (Liao, Smyth, & Shi, 2014) and were then normalized and used for differential gene expression analysis between infected and noninfected lung samples with the DESeq2 package (Love, Huber, & Anders, 2014). The *P*‐values from the differential gene expression analyses were adjusted via Benjamini–Hochberg multiple corrections. An adjusted *p*‐value <0.05 was set as the level of significance.

## 3 RESULTS

### 3.1 Phenotypic statistics of EPL scores and LL levels

Although the Bamaxiang pigs were immunized with several common vaccines, including the classical swine fever, foot‐and‐mouth disease, pseudorabies, porcine reproductive and respiratory syndrome, and porcine circovirus type 2 vaccines, this herd suffered from swine respiratory disease. Similar to the previous study of Erhualian herd (Huang et al., 2017), no typical clinical symptoms of these viral diseases were observed during the entire rearing time on the farm, and the disease‐tolerant period was long, lasting for almost the entire farming stage in Nanchang, and the hot, wet environment could intensify the incidence of respiratory disease (Supporting information Figure S2). We again defined this respiratory disease infecting the Bamaxiang herd as “enzootic pneumonia‐like (EPL) disease”.

**Figure 1:**
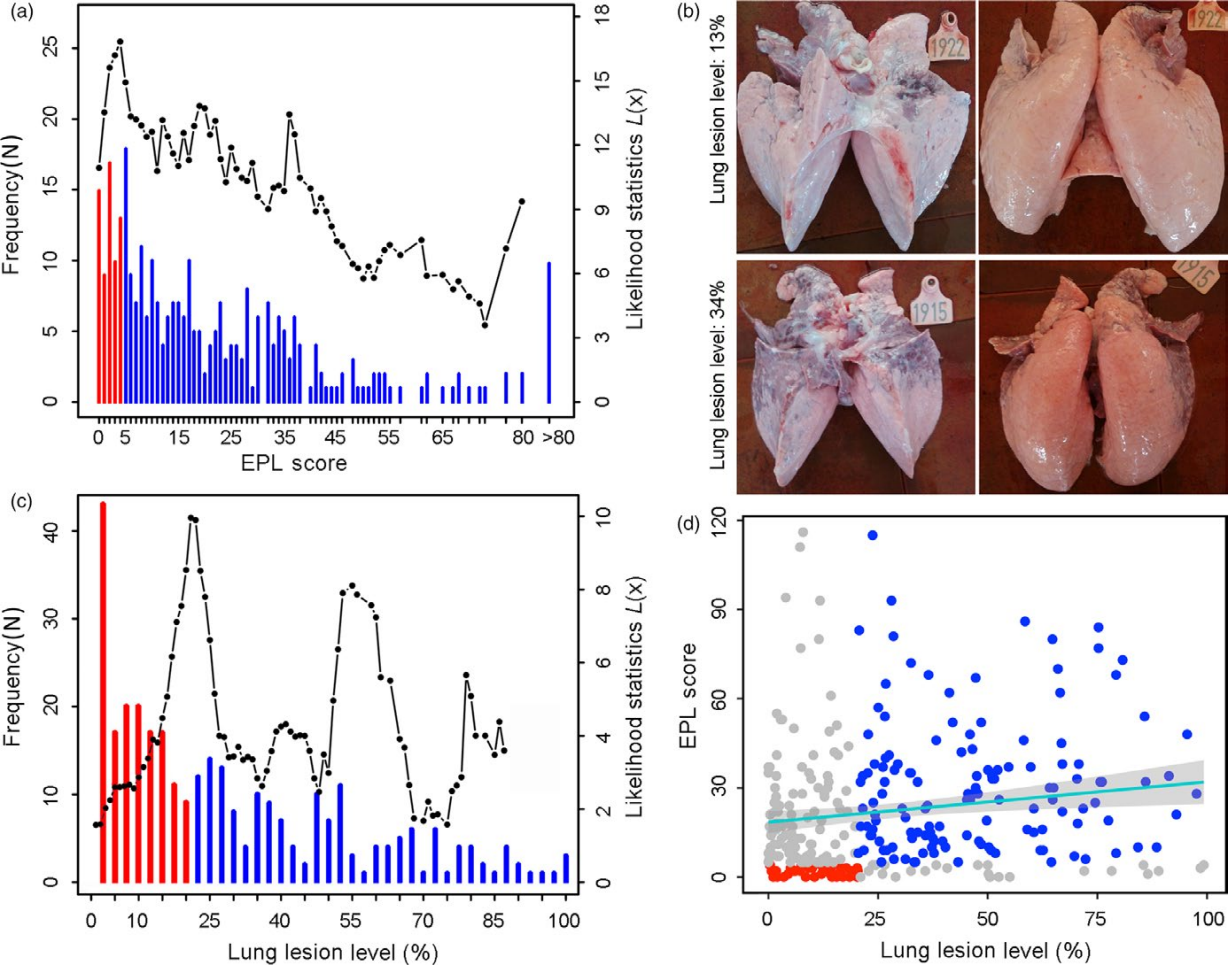
Characteristics of two swine‐respiratory‐disease (SRD)‐related traits in Bamaxiang pigs. *Red* denotes disease‐resistant pigs; *blue* denotes disease‐susceptible pigs; and *gray* denotes pigs that were not classified. (a) Frequency distribution of EPL scores and the likelihood statistic *L(x)*. The average EPL score was 22.3 for the Bamaxiang herd. The likelihood statistic, *L(x)*, was maximal when the grouping threshold of the EPL score was set to 4. The number of animals assigned to the disease‐resistant group (*N*_RG_) was 64, and the number of animals assigned to the disease‐susceptible group (*N*_SG_) was 250. (b) Typical lung lesions of two Bamaxiang pigs suffering from SRD, with LL levels of 13% and 34%. (c) Frequency distribution of LL levels and the likelihood statistic *L(x)*. The maximum and average LL levels were 0.993 and 0.282, respectively, for the Bamaxiang herd. The likelihood statistic, *L(x)*, was maximal when the grouping threshold of LL levels was set to 0.204. *N*_RG_ =154 and *N*_SG_ = 160. (d) Grouping information based on both EPL scores and LL levels. *N*_RG_ = 40 and *N*_SG_ = 136. A small correlation was observed between the EPL scores and LL levels (*r* ^2^ = 0.152, *p* = 6.79 × 10^–3^)

Among the 314 Bamaxiang individuals, only 4.8% (15 individuals) did not appear to exhibit any respiratory symptoms, while 50.3% suffered from severe dyspnea and coughs at least 16 times (Figure 1a). The average EPL score of the Bamaxiang herd was 22.3 (Supporting information Table S2). Regarding LL levels, most of the Bamaxiang pigs suffered from lung lesions to different degrees. As an example, we present two pig lungs with estimated LL levels of 0.13 and 0.34 (Figure 1b). Only five individuals exhibited no lung lesions, and their LL levels were set to 0; the maximum LL level was 0.993; and the average LL level for these Bamaxiang pigs was 0.282 (Figure 1c, Supporting information Table S2). Surprisingly, the phenotypic correlation between EPL scores and LL levels was small (*r* ^2^ = 0.152, *p* = 6.79 × 10^–3^) in the Bamaxiang pig herd (Figure 1d). For example, some pigs with zero EPL scores suffered from severe lung lesions, presenting an LL level of more than 0.50, while some pigs with no lung lesions suffered from severe dyspnea. These findings suggested that there was not a strong relationship between the two traits of swine respiratory disease.

To clarify whether the relationship between EPL scores and LL levels was constant throughout the observed growth period, we divided the growth period into six intervals: age ≤120 days; 120 days <age ≤ 150 days; 150 days <age ≤ 180 days; 180 days <age ≤ 210 days; 210 days <age ≤ 240 days; and age ≥240 days. We performed correlation analysis between EPL scores and LL levels in each interval. We found that there were no correlations between EPL scores and LL levels in the two age intervals of less than or equal to 150 days. The highest correlation (*r* ^2^ = 0.279, *p = *5.13 × 10^–7^) existed within the interval of 180 days <age ≤ 210 days, while those for the other three intervals were approximately 0.117 and were all less than the overall correlation value of 0.152 (Supporting information Figure S3). These findings indicated that the clinical respiratory symptoms presented a stronger relationship with the extent of lung lesions during the growth period from 180 to 210 days than in other growth stages in the Bamaxiang pigs.

### 3.2 Effects of EPL scores and LL levels on weight gain

It has been repeatedly reported that swine respiratory disease affects weight gain in pig farming (Hoy, 1994; Huang et al., 2017). In the present study, we also investigated the effect of respiratory disease on body weights in the Bamaxiang population. We found that both EPL scores and LL levels showed a negative correlation with body weight at the ages of 150, 180, 240, and 300 days (Table 1), and the EPL score generally had a larger negative effect on body weight than LL levels at the above four ages.

**Table 1:** Weight gain influenced by enzootic pneumonia‐like (EPL) scores, lung lesion (LL) levels, and both parameters in Bamaxiang pigs

Grouping method	Age (day)	Body weight (kg)		Weight gain reduction (%)	*p*‐value
		Healthy pigs	Affected pigs		
EPL scores	150	24.62 ± 3.98	20.67 ± 3.92	16.0	1.21 × 10^−7^
	180	32.00 ± 6.01	27.37 ± 4.80	14.5	1.70 × 10^−7^
	240	49.48 ± 7.28	45.86 ± 7.74	7.3	8.47 × 10^−4^
	300	61.71 ± 8.16	59.61 ± 10.00	3.4	8.00 × 10^−2^
LL levels	150	22.10 ± 3.91	21.02 ± 4.63	4.9	1.08 × 10^−1^
	180	29.59 ± 5.59	27.17 ± 4.96	8.2	1.04 × 10^−4^
	240	48.30 ± 7.27	44.96 ± 7.87	6.9	3.48 × 10^−4^
	300	61.44 ± 9.50	58.60 ± 9.62	4.6	9.00 × 10^−3^
EPL scores and LL levels	150	24.50 ± 3.75	20.42 ± 4.40	16.7	7.66 × 10^−6^
	180	32.76 ± 6.18	26.62 ± 4.67	18.7	3.61 × 10^−7^
	240	50.48 ± 7.17	44.49 ± 8.01	11.9	3.68 × 10^−5^
	300	62.30 ± 8.18	58.32 ± 9.90	6.4	1.20 × 10^−2^

To perform genetic differentiation analyses in this Bamaxiang population, we applied three strategies for dividing the herd into two groups, in which the pigs were divided into disease‐susceptible and disease‐resistant groups based on EPL scores or LL level, or both EPL scores and LL levels (Table 1). In the grouping method based on the EPL score, the likelihood statistic was maximized when the threshold of the EPL score (*x*) was set to 4 (Figure 1a). We compared the body weights of the disease‐susceptible pigs (EPL score>4) and disease‐resistant pigs (EPL score ≤4). Significant differences were found between the two groups at the ages of 150, 180, and 240 days (*p < *0.01), but not at the age of 300 days (*p* = 0.08). The disease‐susceptible group exhibited 16.0% (day 150), 14.5% (day 180), and 7.3% (day 240) lower body weights compared with the disease‐resistant group. In the grouping method based on LL levels, the likelihood statistic was maximized when the threshold of the LL level (*y*) was set to 0.204 (Figure 1c). Significant differences were found between the two groups at days 180, 240, and 300 (*p* < 0.01), but not at day 150 (*p* = 0.11). In the grouping method based on both EPL scores and LL levels (Figure 1d), significant differences were found between the two groups at all ages (150, 180, 240, and 300 days). The body weights of the disease‐susceptible group decreased by 16.7% (150 days), 18.7% (180 days), 11.9% (240 days), and 6.4% (300 days). These findings indicated that this respiratory disease has a persistent influence on the weight gain of disease‐susceptible pigs during the growth period, and the combined effect of EPL scores and LL levels on weight gain was greater than each single effect.

### 3.3 Candidate loci detected through genetic differentiation analyses

**Figure 2:**
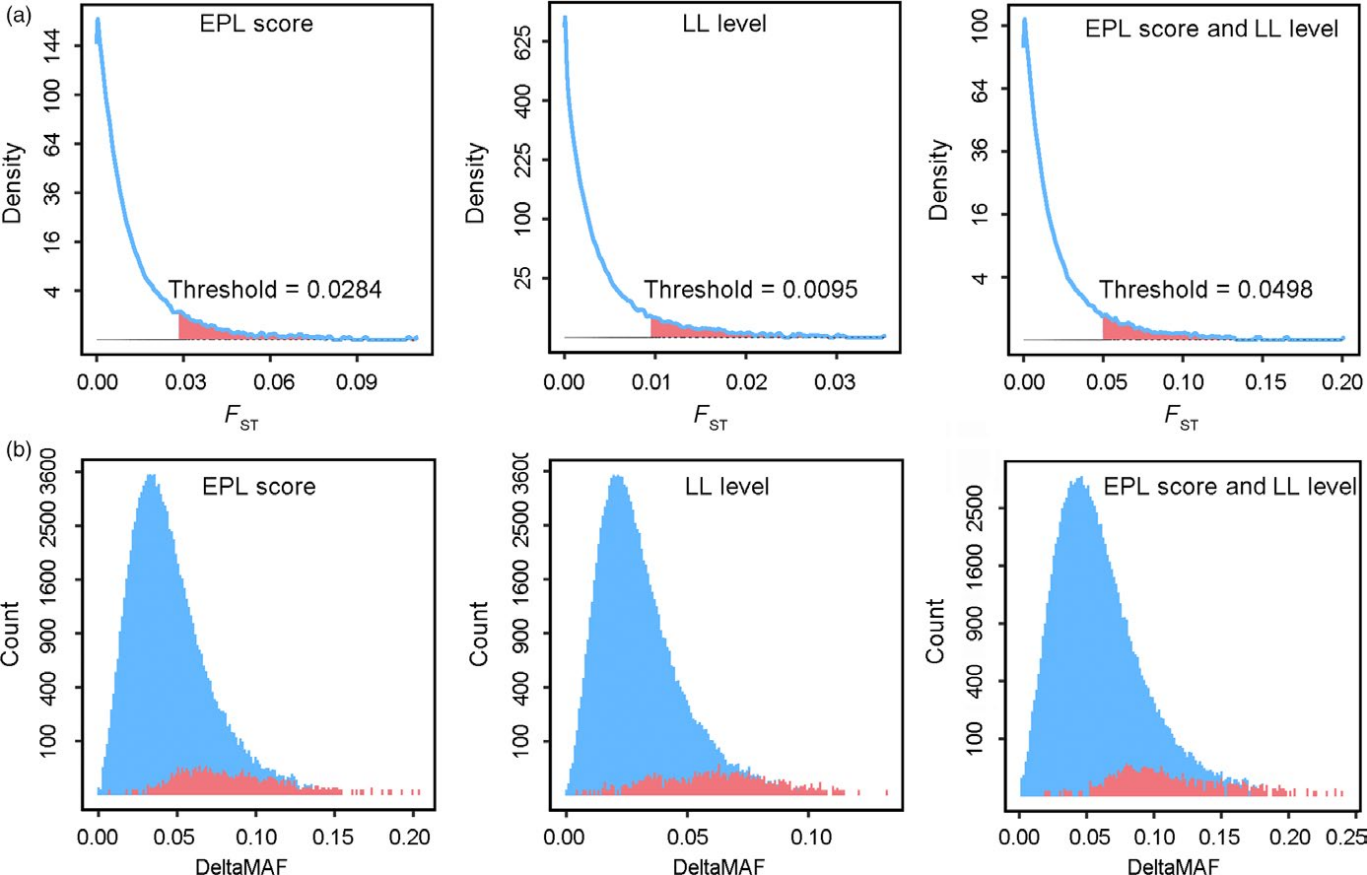
Genetic differentiation analyses in the Bamaxiang population. (a) Density curve of *F*_ST_ values between disease‐susceptible pigs and disease‐resistant pigs; the red portion indicates loci above than the threshold of 1%. The F_ST_ value of 0.0284 was set as the top 1% threshold for the EPL score grouping method; 0.0095 was set as the top 1% threshold for the LL level grouping method; and 0.0498 was set as the top 1% threshold for the grouping method based on both EPL scores and LL levels. (b) Distribution of deltaMAF values. The blue histogram represents all loci; the red histogram represents the loci with genetic differentiation values surpassing the thresholds

We estimated the genetic differentiation of the fixation index (*F*_ST_) within these three‐paired animal groups for 93,425 loci throughout the genome (Supporting information Figure S4). Under each grouping method, the *F*_ST_ distribution was highly skewed toward zero and exhibited a long tail (Figure 2a). In the grouping method based on the EPL score, the maximum *F*_ST_ value was 0.1109, and the mean was 0.0051; based on LL levels, the maximum *F*_ST_ value was 0.0354, and the mean was 0.0015; and based on both EPL scores and LL levels, the maximum *F*_ST_ value was 0.2015, and the mean was 0.0085. The thresholds of the top 1% of loci were 0.0284, 0.0095, and 0.0498 for the grouping methods based on EPL scores, LL levels, and both EPL scores and LL levels, respectively.

To test whether the higher *F*_ST_ values resulted from a true null distribution of genetic differentiation or not, we performed 1,000‐times permutation tests for all three grouping strategies. We observed that the top 1% of the *F*_ST_ thresholds of the real data were more stringent than the thresholds of *p* = 0.005 set based on permutation tests. In detail, the mean of F_ST_ values of the permutation tests at *p* = 0.005 was 0.0132 for grouping based on EPL scores, 0.0086 for grouping based on LL levels, and 0.0215 for both EPL scores and LL levels, which were lower than the top 1% *F*_ST_ thresholds of EPL scores (0.0284), LL levels (0.0095), and both EPL scores and LL levels (0.0498). Therefore, these permutation tests confirmed that the large *F*_ST_ loci between the disease‐resistant and disease‐susceptible subgroups were not simply due to normal neutral variation but were more likely a selection result of human‐driven or SRD‐pathogen selection pressure.

To validate the reliability of the candidate loci we identified, we also examined the mean deltaMAF in the candidate regions. We found that the deltaMAF distribution of the candidate loci was generally skewed toward higher deltaMAF values and showed a significant difference compared with the overall values for all genomic loci (Figure 2b). These differences confirmed the results of population genetic differentiation analysis between the disease‐resistant and disease‐susceptible groups of the Bamaxiang population.

### 3.4 Overview of candidate genes for host adaptation to SRD

**Figure 3:**
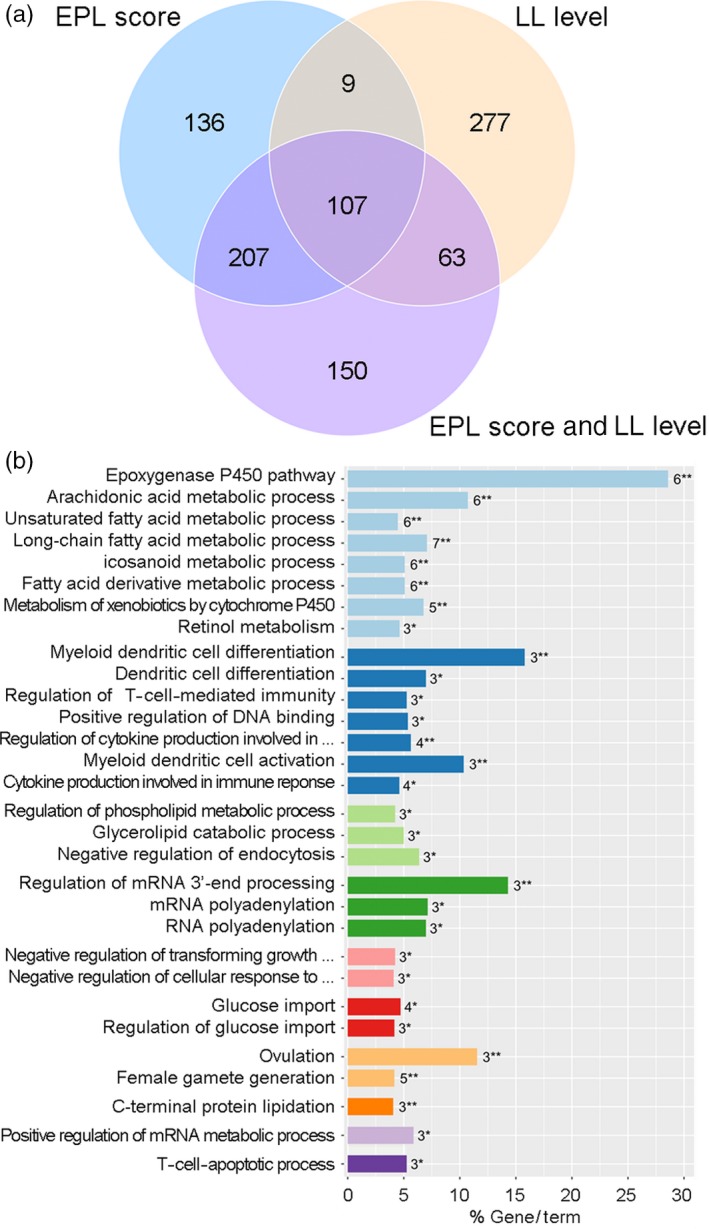
The 107 common candidate genes and their enriched gene ontology terms and KEGG pathways. (a) Venn diagram showing that a total of 107 candidate genes were common to the top 1% of loci obtained under the three different grouping methods. A list of these 107 genes is provided in Supporting information Table S7. (b) Gene ontology terms and KEGG pathways were enriched using these 107 common genes. The enriched terms are color‐coded to reflect relatedness in the ontology or functional proximity. All GO and KEGG terms are listed in Supporting information Table S4

A total of 459, 456, and 527 candidate genes (Supporting information Table S3) were identified and annotated for the animal grouping methods based on EPL scores (EPL), LL levels (LL), and both parameters (EPL_LL), respectively. The top 10 candidate genes detected in the EPL grouping method were *PPP2R5E*, *CERS3*, *CCDC73*, *EIF3M*, *ETFDH*, *PPID*, *MECP2*, *FSHB*, *IRAK1*, and *FAM133A*; the top 10 candidate genes in the LL grouping method were *HS6ST2*, *GAA*, *USP26*, *CCDC40*, *C3ORF70*, *AR*, *CARD14*, *RNF213*, *SGSH*, and *SLC26A11*; and the top 10 candidate genes in the EHL_LL grouping method were *CERS3*, *ETFDH*, *PPID*, *CIC*, *PAFAH1B3*, *PRR19*, *PPP2R5E*, *FAM133A*, *DEDD2*, and *ERF *(Supporting information Figure S4). Of the above 459, 456, and 527 candidate genes, 314 were commonly detected in the EPL and EPL_LL grouping methods; 116 were shared between the EPL and LL grouping methods; 170 were shared between the LL and EPL_LL grouping methods; and 107 were shared among all three grouping methods (Figure 3a). Using these 107 common candidate genes as an input gene list, 28 significant GO terms and two significant KEGG pathways were enriched (Supporting information Table S4), which were clustered into ten GO groups (Figure 3b).

Notably, two GO groups related to host immune responses and fatty acid metabolism were identified. The GO group for host immune responses included seven biological processes: myeloid dendritic cell differentiation (GO:0,043,011), myeloid dendritic cell activation (GO:0001773), regulation of cytokine production involved in immune response (GO:0,002,718), cytokine production involved in immune response (GO:0,002,367), dendritic cell differentiation (GO:0,097,028), regulation of T‐cell‐mediated immunity (GO:0,002,709), and positive regulation of DNA binding (GO:0,043,388). Seven overrepresented genes participated in these biological processes: *RELB*, *TGFB1*, *TRAF6*, *CLC*, *FFAR3*, *NECTIN2*, and *PLAUR*. For fatty acid metabolism, the GO group involved six biological processes and two KEGG pathways: the epoxygenase P450 pathway (GO:0,019,373), arachidonic acid metabolic process (GO:0,019,369), long‐chain fatty acid metabolic process (GO:0001676), fatty acid derivative metabolic process (GO:1,901,568), icosanoid metabolic process (GO:0,006,690), unsaturated fatty acid metabolic process (GO:0,033,559), metabolism of xenobiotics by cytochrome P450 (GO:0,000,980), and retinol metabolism (GO:0,000,830). Seven genes were also overrepresented in these biological processes or pathways: *CYP2A13*, *CYP2A6*, *CYP2A7*, *CYP2B6*, *CYP2F1*, *CYP2S1*, and *LIPE*. In addition, three biological processes related to lipid metabolism, two glucose import processes, and two growth factor signaling pathways were enriched (Figure 3b). These findings suggested that the genes involved in the above GO processes and KEGG pathways might play important roles in the adaptation of pigs to swine respiratory disease.

To investigate the interaction networks of candidate genes related to host adaptation to SRD, we used the protein–protein interaction (PPI) database from STRING (Szklarczyk et al., 2015) to construct undirected graphs of gene coding proteins obtained from the three different grouping methods. We detected 295 nodes and 528 edges of interactions in the protein network for the EPL score grouping methods using 459 candidate gene coding proteins; 299 nodes and 593 edges for the LL level grouping method using 456 candidate proteins; and 338 nodes and 607 edges for the grouping method based on both EPL scores and LL levels using 527 candidate proteins. Subsequently, we searched for network biomarkers and determined the core network from the above PPI networks via centrality analyses. For the EPL score grouping method, 113 nodes were identified as hub genes due to high node degrees surpassing the threshold of 3.58; 69 nodes were defined as bottlenecks due to higher betweenness scores surpassing the threshold of 754.59; and 102 nodes showed high eigenvector values surpassing the threshold of 0.036. Finally, 55 nodes representing 18.6% of the initial PPI networks showed higher centrality values for the three parameters of degree, betweenness, and eigenvector (Supporting information Figure S5A). For the LL level grouping method, 118 and 69 nodes were identified as hub genes and bottlenecks, respectively, and 97 nodes showed high eigenvector values surpassing the threshold of 0.035. Finally, 48 nodes participating in 16.1% of the initial PPI networks showed high centrality values (Supporting information Figure S5B). For the grouping methods based on both EPL scores and LL levels, 122 nodes were detected as hub genes; 86 nodes were defined as bottlenecks; and 94 nodes showed high eigenvector values surpassing their thresholds. Furthermore, 55 nodes corresponding to 16.3% of the initial PPI networks showed high centrality values for all centrality parameters (Supporting information Figure S5C). Taking all of these findings together, we observed the intersection of the results of the three different grouping methods and ultimately obtained seven common genes (*TGFB1*, *SNRPA*, *TRAF6*, *CYP2A6*, *CD44*, *CYP2B6*, and *PLAUR*) with high centrality values (Supporting information Figure S5D).

We further investigated the SNP effects of these common genes on both SRD‐related traits of EPL scores and LL levels. First, we examined the genotypes of the SNPs with largest F_ST_ values located in the candidate loci harboring these common genes, which were defined here as the genotypes of the corresponding genes. Five candidate genes (*TGFB1*, *SNRPA*, *TRAF6*, *CD44*, and *CYP2B6*) exhibited only two genotypes (Supporting information Table S5). Association analysis revealed significant differences in EPL scores between the two genotypes of *TGFB1*, *SNRPA*, *TRAF6*, *CYP2B6*, and *CD44*, with *p*‐values of 1.01 × 10^–2^, 1.13 × 10^–3^, 4.35 × 10^–4^, 7.37 × 10^–4^, and 1.67 × 10^–4^, respectively. Significant differences in LL levels also existed between the two genotypes of *TGFB1*, *TRAF6*, *CYP2B6*, and *CD44*, with *p*‐values of 7.48 × 10^–3^, 2.57 × 10^–3^, 2.41 × 10^–2^, and 1.37 × 10^–2^, respectively. In addition, we found another gene coding for a family member of the sialic acid binding lectins among 107 common candidate genes, the *CD22* gene, showing significant differences between the EPL scores of its three genotypes (*p = *1.28 × 10^–3^). When the combined effects of *CD22* and *CD44* were considered, a more significant difference (*p* = 4.78 × 10^–5^) in EPL scores was found among the individuals with different genotypes (Supporting information Table S6) than was observed for the independent effects of *CD22* and *CD44*.

### 3.5 Differential gene expression in the lungs

**Figure 4:**
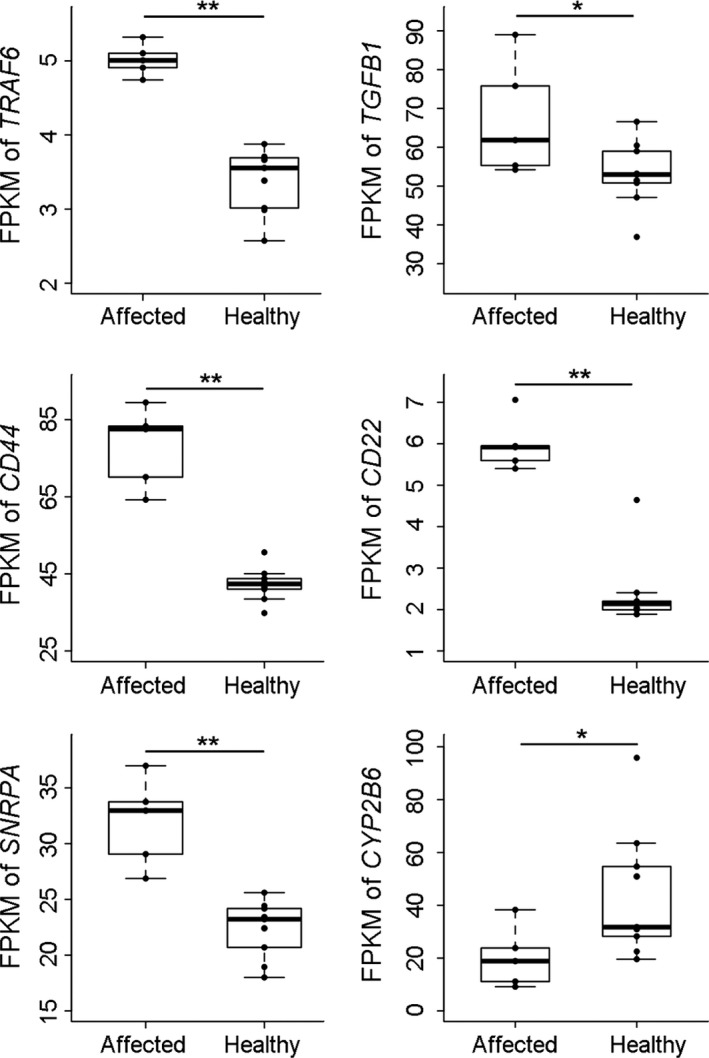
Expression of the six highlighted genes in healthy lung tissues and affected lung lesions. The differences in the expression of *TRAF6*, *CD44*, *CD22*, and *SNRPA* between healthy lung tissues and affected lung lesions were extremely significant, with corrected *P*‐values of 2.09 × 10^–5^, 5.24 × 10^–15^, 4.71 × 10^–10^, and 6.48 × 10^–5^, respectively. *TGFB1* and *CYP2B6 *were significantly differentially expressed in healthy lung tissues and affected lung lesions, with corrected *p*‐values of 1.89 × 10^–2^ and 4.88 × 10^–2^, respectively

To gain insight into the mechanism whereby pig lungs respond to SRD infection, we generated and analyzed transcriptomic data from nine healthy lung tissues and five affected lung lesions collected from a commercial black pig population under common indoor farming conditions. Hierarchical cluster analyses showed that the transcriptomic profiles of the healthy and affected lung samples differed from each other, and the healthy and affected lung samples were grouped on their own branches (Supporting information Figure S6A). A total of 23,382 *Sus scrofa* reference genes downloaded from the Ensembl database (ftp://ftp.ensembl.org/pub/release-75/gtf/sus_scrofa/) and 20,564 predicted genes generated with Cufflinks software were used for the differential expression analysis. Among these genes, 5,894 reference genes (2,716 upregulated, 3,178 downregulated) and 2,137 predicted genes (1,416 upregulated, 721 downregulated) were detected as being differentially expressed between the infected and noninfected lung tissues. In summary, 4,132 and 3,899 genes were up‐ and downregulated in healthy tissues compared with infected tissues, respectively (Supporting information Figure S6B). Notably, among the above 107 common candidate genes shared by the three grouping methods and 158 key node genes highlighted through PPI analysis, 72 genes were identified as differentially expressed genes (Supporting information Table S7). *TGFB1*, *TRAF6*, *CD44*, *CD22*, *SNRPA*, and *CYP2B6* were highlighted as the six most essential genes in this set of differentially expressed genes, which were downregulated 1.37‐, 1.47‐, 1.83‐, 2.45‐, and 1.42‐fold or upregulated 1.94‐fold in lung lesions, with adjusted *p*‐values of 1.89 × 10^–2^, 2.09 × 10^–5^, 5.24 × 10^–15^, 4.71 × 10^–7^, 6.48 × 10^–5^, and 4.88 × 10^–2^, respectively (Figure 4, Supporting information Table S7).

## 4 DISCUSSION

### 4.1 The Bamaxiang pigs were under selection pressure in the SRD environment

In the present study, we defined an SRD environment as the biological and physical factors, along with their interactions, that cause pigs to develop swine respiratory disease. We consider pigs to always be in an SRD environment under common indoor farming conditions because of the major reasons indicated below.

First, the lungs are among the open organs that closely interact with the outside environment, including bacteria, viruses, dust, and other xenobiotics. Diverse communities of microbes are found in the lower respiratory tract particularly on the mucus layer and epithelial surfaces, whether the lungs are healthy or not (Dickson, Erbdownward, & Huffnagle, 2013). Under common indoor farming conditions, pigs are generally in a state of reciprocal interaction and struggle with environmental microbes. The complex variety of microorganisms in the respiratory tract often leads to swine respiratory disease in pigs. Second, swine respiratory disease is prevalent on global high‐density pig farms and difficult to control. Historical records show that a pandemic of enzootic pneumonia in local Chinese pigs began in the late of 1950s, resulting in mortality rates of 20.3%, 38.7%, and 31.6% for pigs with body weights of more than 50 kg, 20 to 50 kg, or <20 kg, respectively (Chinese Academy of Agricultural Sciences, 1958). Contagious respiratory disease is one of the common diseases plaguing local Chinese pigs during rearing. Third, we observed that most of the Bamaxiang pigs (93.2%) in our study suffered from asthma and that lesions occurred in 98.4% of their lungs. Respiratory symptoms appeared throughout the farming period and were most severe at an age of ~160 days, during the hottest and wettest season (June and July), at which time the highest frequency of cough occurred in this population.

Furthermore, we believe that human‐driven selection pressure and swine respiratory disease have shaped the evolutionary trajectory of these Bamaxiang pigs. In the breeding of local Chinese pigs, breeders prefer to choose healthy, normal, or fast‐growing pigs to reproduce subsequent generations. Disease‐resistant pigs would have a greater opportunity to be selected as sires or sows to generate offspring. In addition, during the rearing period, some pigs suffering from severe lung lesions will be easy to die if simultaneously infected by secondary or opportunistic pathogens. In the present study, some Bamaxiang pigs died of swine respiratory disease in the farming process. Unfortunately, these pigs were not included in this study because they had no available genotypic data and no phenotypic records.

Considering these findings together, we believe that the Bamaxiang pigs were under selection pressure in the SRD environment. These pigs generally experienced a long‐term mutual struggle with respiratory disease pathogens under common indoor feeding conditions. In the SRD environment, these pigs could be genetically classified into two subgroups: one resistant to SRD and another susceptible to swine respiratory disease. The related genes may have differentiated during pig adaptation to respiratory disease, and their allele frequencies might also have changed.

### 4.2 SRD phenotypes and their effects on weight gain

Previously, there were two major methods for determining the extent of swine respiratory disease through genetic analyses: one based on the count or frequency of asthma cases (Huang et al., 2017) and the other based on the scoring of lung lesions (Okamura et al., 2012). Here, we employed both methods to estimate the extent of SRD suffering in a Chinese Bamaxiang pig herd. Compared to previous findings in Erhualian pigs, the EPL scores of the Bamaxiang pigs (average value: 22.3) were generally higher than those of Erhualian pigs (average value: 3.5), possibly due to differences in germplasms and/or statistical batches, or other factors. This observation suggested that Bamaxiang pigs might be more susceptible to swine respiratory disease than Erhualian pigs under common indoor farming conditions. Unexpectedly, we found that the phenotypic correlation between EPL scores and LL levels was as low as 0.152 in the Bamaxiang pig population, which indicated that these two traits might reflect different impacts of swine respiratory disease on pigs. Similarly, a low correlation between clinical signs of respiratory system disorders and lung lesions at slaughter was previously observed in veal calves (Leruste et al., 2012). Both traits exerted a negative effect on the weight gain of Bamaxiang pigs, which was consistent with previous reports from other pig breeds (Fang et al., 2015; Huang et al., 2017). We considered the EPL scores to exert a more adverse effect on weight gain than LL levels, due to greater differences between the disease‐resistant and disease‐susceptible pigs when grouped by EPL scores. Furthermore, the superposed effect of EPL scores and LL levels together was larger than each single effect alone.

### 4.3 Candidate genes for host adaptation to SRD

Under general indoor farming conditions, pigs usually live in a microbe‐rich environment, and interactions will occur between the host and microbes. The host immune system will be triggered to cope with the invasion of microorganisms, and the microbes in turn influence the host, such as through adverse effects on host growth due to SRD infection. In this study, we used genetic differentiation analyses to identify candidate genes responding to host adaptation to SRD. We identified 107 common candidate genes using three animal grouping methods based on EPL scores, LL levels, and both parameters. Interestingly, two major groups of biological processes, related to host immune responses and fatty acid metabolism, were enriched, which was consistent with the interaction between the host and microbes. These results also indicated that the genes involved in these biological processes might play important roles in host adaptation to SRD.

For host immune responses, two enriched biological processes, myeloid dendritic cell differentiation (GO:0043011) and myeloid dendritic cell activation (GO:0001773), overrepresented by *RELB*, *TGFB1*, and *TRAF6*, might play important roles in the initiation of immunity in response to swine respiratory disease, because it has been reported that myeloid dendritic cells pick up antigens at the periphery and travel to T‐cell areas to initiate immunity (Steinman & Inaba, 1999). Additionally, the biological processes of cytokine production and regulation of cytokine production involved in the immune response were enriched by the overrepresented genes *CLC, FFAR3, TGFB1*, and *TRAF6*. For fatty acid metabolism, six related GO terms were enriched by the cytochrome P450 family 2 genes *CYP2A6*, *CYP2A7*, *CYP2A13*, *CYP2B6*, *CYP2F1*, and *CYP2S1*; these terms included the epoxygenase P450 pathway, arachidonic acid metabolic process, unsaturated fatty acid metabolic process, long‐chain fatty acid metabolic process, icosanoid metabolic process, and fatty acid derivative metabolic process. In addition to their involvement in fatty acid metabolism, cytochrome P450 (CYP) enzymes often play a dominant role in target tissue metabolic activation of xenobiotic compounds in the respiratory and gastrointestinal tracts (Ding & Kaminsky, 2003). Furthermore, the enriched biological process of regulation of phospholipid metabolic process, overrepresented by *APOC1*, *APOC2*, and *TGFB1*, is related to the host immune response to microbial infection, as phospholipids have proven to be essential in inflammation (Nixon, 2009).

We further applied centrality analyses to the top 1% of genes identified under the EPL score, LL level, and both EPL score and LL level grouping methods. A total of 55, 48, and 55 genes showed higher centrality values for the parameters of degree, betweenness, and eigenvector, respectively, among which seven genes coexisted under the three classification methods. Notably, six genes (*TRAF6*, *CD44*, *CD22*, *TGFB1*, *SNRPA*, and *CYP2B6*) were highlighted as strong candidate genes due to showing differential expression between healthy lung tissue and lung lesion tissue, along with their structural and functional centrality in the biological processes of immune responses or fat metabolism.

*TRAF6* encodes tumor necrosis factor receptor (TNFR)‐associated factor protein 6, which functions as a signal transducer in the NF‐kappa B pathway that activates I kappa B kinase in response to proinflammatory cytokines (Cao, Xiong, Takeuchi, Kurama, & Goeddel, 1996). TRAF6‐mediated signals have proven critical for the development, homeostasis, and/or activation of B cells, T cells, and myeloid cells, as well as for organogenesis of thymic and secondary lymphoid tissues (Walsh, Lee, & Choi, 2015). In addition, TRAF6 was demonstrated to be related to the pathways of bacterial infections in cystic fibrosis airways (Walsh et al., 2001). CD44 is a transmembrane adhesion receptor and serves as the major cell‐surface receptor for the nonsulfated glycosaminoglycan hyaluronan (Aruffo, Stamenkovic, Melnick, Underhill, & Seed, 1990). CD44 is involved in recruiting T cells to inflammatory sites and regulates T‐cell‐mediated endothelial injury (Degrendele, Kosfiszer, Estess, & Siegelman, 1997; Rafi‐Janajreh et al., 1999). Teder et al. (2002) demonstrated a role of CD44 in resolving the inflammatory response following lung injury. CD22 is also a transmembrane sialo‐adhesion protein expressed by nearly all mature B cells as well as a member of the immunoglobulin superfamily (Engel, Wagner, Miller, & Tedder, 1995). In humans, *CD22* has been observed to positively associate with regional emphysema severity of the lungs and is regarded as a potential causal gene for airflow obstruction (Lamontagne et al., 2014). *TGFB1* encodes a secreted ligand of the transforming growth factor‐beta superfamily of proteins. The *TGFB1* gene product is a secreted protein with numerous functions, including roles in lung development and disease (Bartram & Speer, 2004); TGFB1 appears to be the predominant isoform involved in pulmonary fibrosis (Bartram & Speer, 2004). In mice, an overdose of *TGFB1* transmission resulted in disruption of epithelial differentiation and inhibitory synthesis of the Clara cell secretory protein, phospholipids, and surface proteins A, B, and C. In humans, the T allele of the SNP rs1800469 in the promoter region of the *TGFB1* gene has been associated with phenotypes related to asthma (Silverman et al., 2004), and *TGFB1* levels in the airways of asthmatics are reported to be higher than in normal subjects (Nakao, 2001). Similarly, the expression of *TGFB1* in affected pig lungs was found to be significantly higher than that in healthy lungs in the present study. *SNRPA* encodes small nuclear ribonucleoprotein polypeptide A, which has been reported to function in the binding stem loop II of the U1 snRNA and might be involved in coupled premRNA splicing and polyadenylation (Jessen, Nagai, Oubridge, Teo, & Pritchard, 1991). Recently, many reports have indicated that *SNRPA* is highly associated with tumors and apoptosis (Tewari, Beidler, & Dixit, 1995). *CYP2B6* encodes a member of the cytochrome P450 superfamily of enzymes. The cytochrome P450 proteins are monooxygenases that catalyze many reactions involved in drug metabolism and the synthesis of cholesterol, steroids, and other lipids. Altogether, these six genes could be considered as strong candidates associated with pig adaptation to SRD.

## 5 CONCLUSIONS

In this study, we first investigated the relationship of two SRD‐related traits, enzootic pneumonia‐like (EPL) scores and lung lesion (LL) levels in a Bamaxiang population. We found that there was no strong relationship between EPL scores and LL levels in the Bamaxiang population. Swine respiratory disease has a persistent influence on weight gain in disease‐susceptible pigs during the growth period, and the joint effect of EPL scores and LL levels on weight gain was found to be greater than each single effect alone. By performing genetic differentiation analyses, we identified 107 common candidate genes, which are mainly involved in biological processes related to the immune response, fatty acid metabolism, lipid metabolism, and growth factor signaling pathways. Among these candidate genes, six genes (*TRAF6*, *CD44*, *CD22*, *TGFB1*, *CYP2B6*, and *SNRPA*) were highlighted as the most essential genes related to pig adaptation to SRD due to their central regulatory roles in the host immune response or fat metabolism and their differential expression between healthy lung tissues and lung lesion tissues.

In the present study, we attempted to reveal the molecular mechanism of mammalian adaption to a chronic disease through genetic differentiation and RNA sequencing analyses. Our results advance the understanding of the genetic mechanisms of host resistance or susceptibility to swine respiratory disease.

## CONFLICT OF INTEREST

None Declared.

## Data Availability

RNA‐seq data for this study are available at the NCBI database with the access number PRJNA496272.
