# Supplementary material for: New insights into host adaptation to swine respiratory disease revealed by genetic differentiation and RNA sequencing analyses
Source: Evol Appl. 2018 Dec 3;12(3):535–48. doi: 10.1111/eva.12737 (PMC6383736; doi:10.1111/eva.12737)
Supplement: Supplementary file 1 [file EVA-12-535-s001.docx]

**Supplementary materials for “New insights into host adaptation to swine respiratory disease revealed by genetic differentiation and RNA sequencing analyses”**


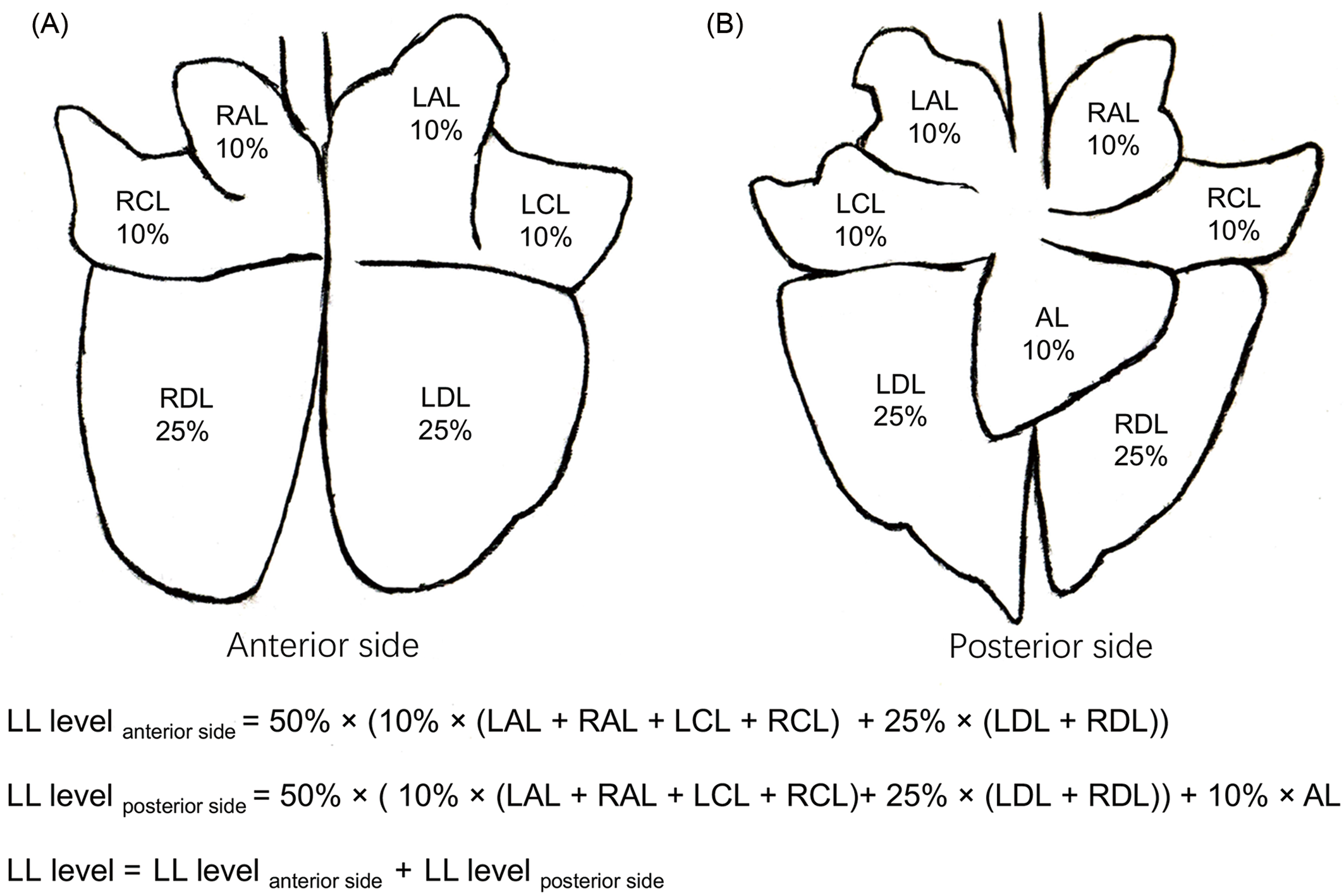


**Figure S1.** **The formula used to** **calculate lung lesion (LL) levels.** LAL denotes left apical lobe, LCL denotes left cardiac lobe, LDL denotes left diaphragmatic lobe, RAL denotes right apical lobe, RCL denotes right cardiac lobe, RDL denotes right diaphragmatic lobe, and AL denotes accessory lobe.


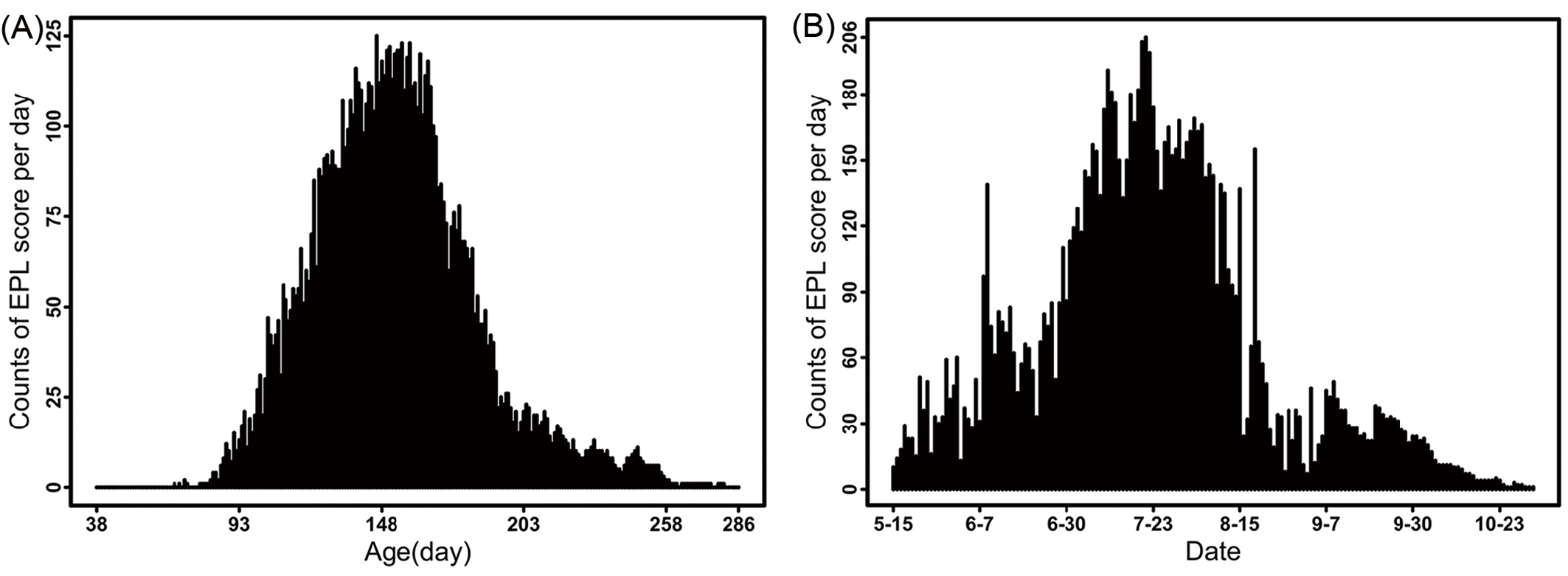


**Figure S2.** **Clinical records of enzootic pneumonia-like (EPL) scores in Bamaxiang pigs.** (A) EPL scores per pig per day were recorded along with its ages during the rearing period; the highest EPL score occurred at the age of approximate 160 days. (B) Date distribution of EPL scores; the peak of EPL scores was observed in the middle of July, the muggiest days in Nanchang city.


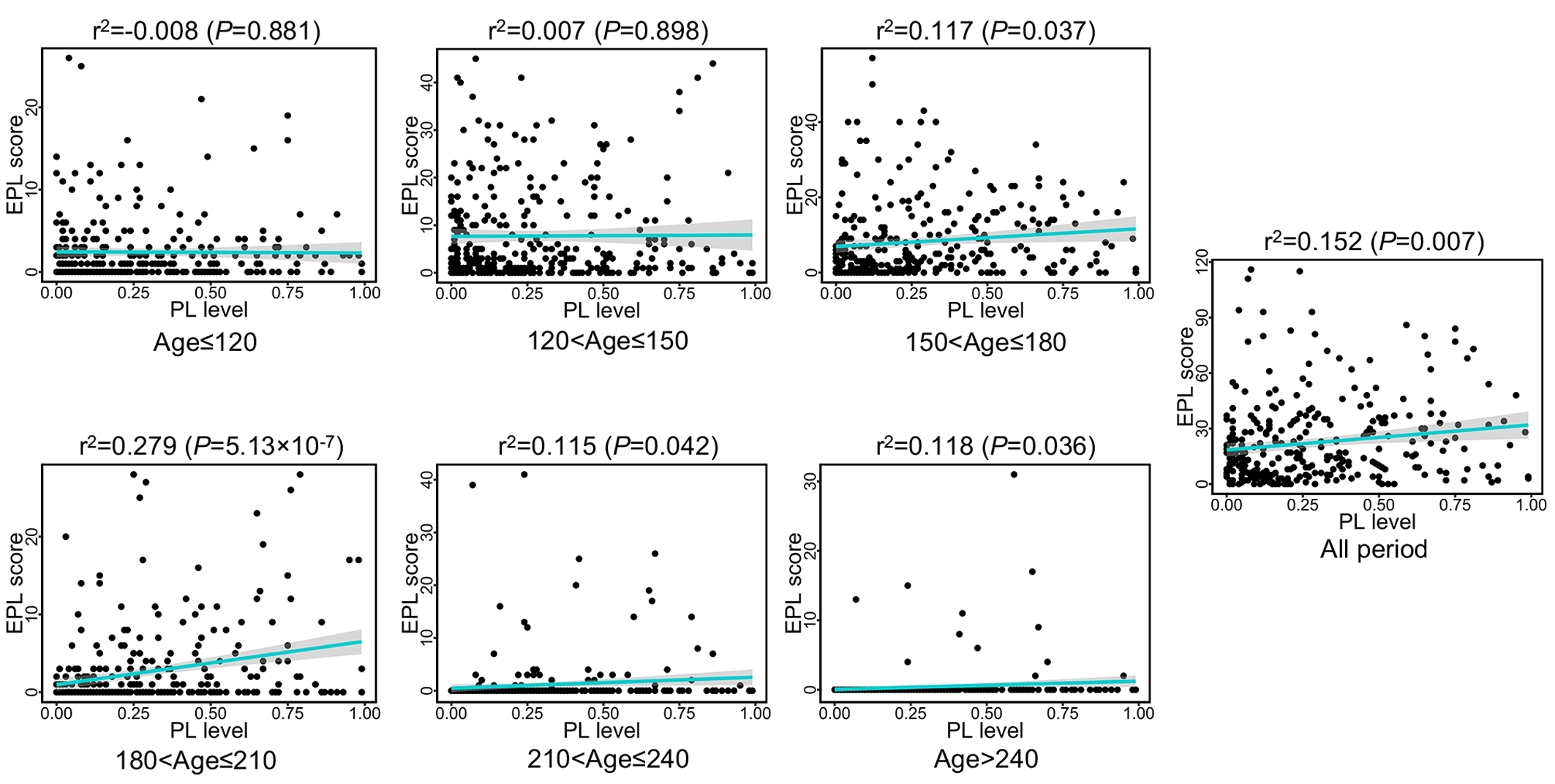


**Figure S3. Correlation tests between EPL scores and LL levels at different growth periods.** The largest correlation (r^2^ = 0.279, *P* = 5.13 × 10^-7^) existed within the interval of 180 days < age ≤ 210 days, which was higher than the overall correlation value of 0.152.


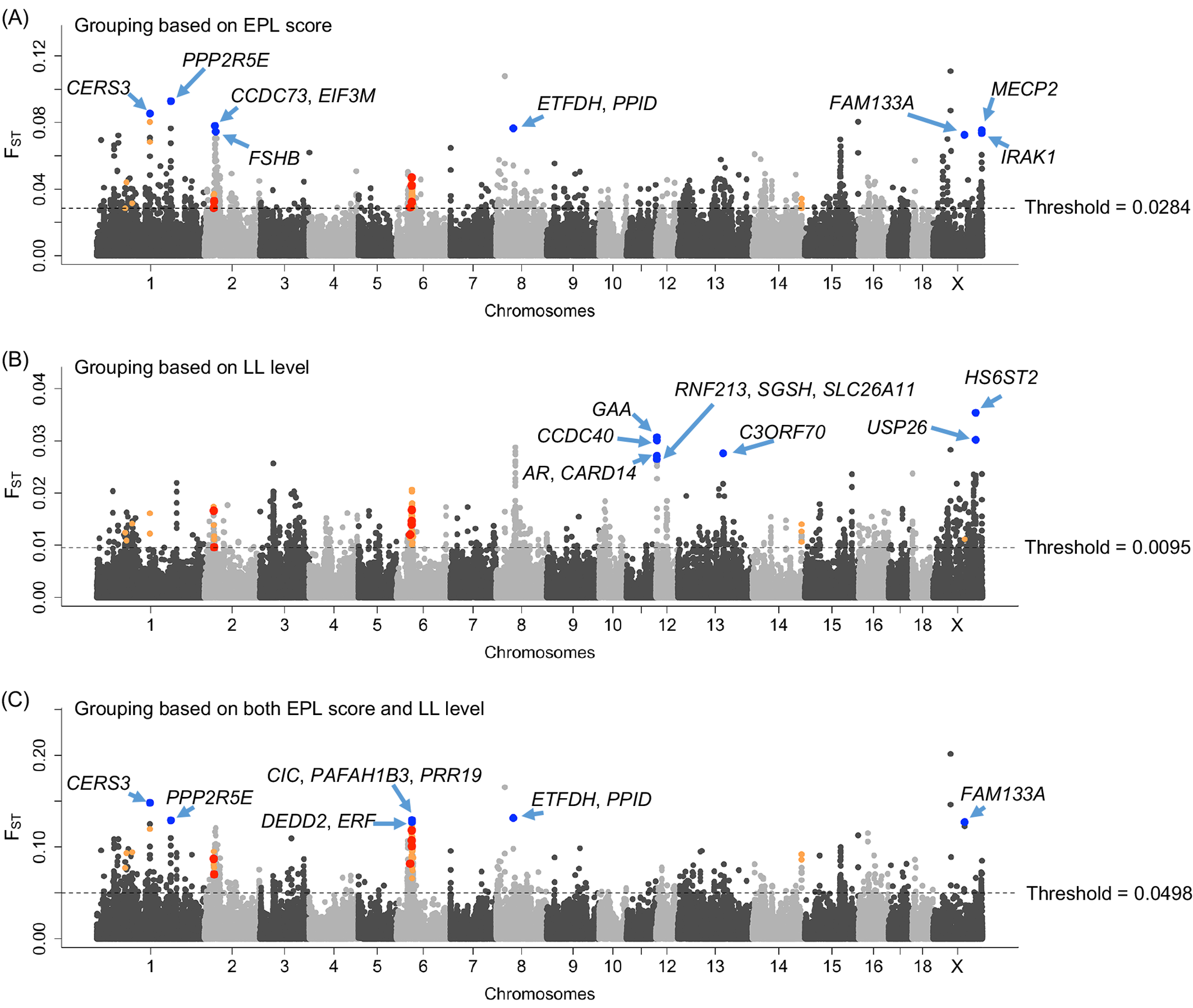


**Figure S4. Manhattan plots of F_ST_ values throughout the genome for three pairs of SRD-resistant and susceptible groups.** *Red* dots denote the 6 candidate genes; *orange* dots denote the 107 common genes identified in three different grouping methods based on EPL scores, LL levels and both parameters in Bamaxiang pigs; *blue* dots denote the top 10 candidate genes in three different grouping methods. (A) Manhattan plots of F_ST_ values for the grouping method based on EPL scores. (B) Manhattan plots of F_ST_ values for the grouping method based on LL levels. (C) Manhattan plots of F_ST_ values for the grouping method based on both EPL scores and LL levels.


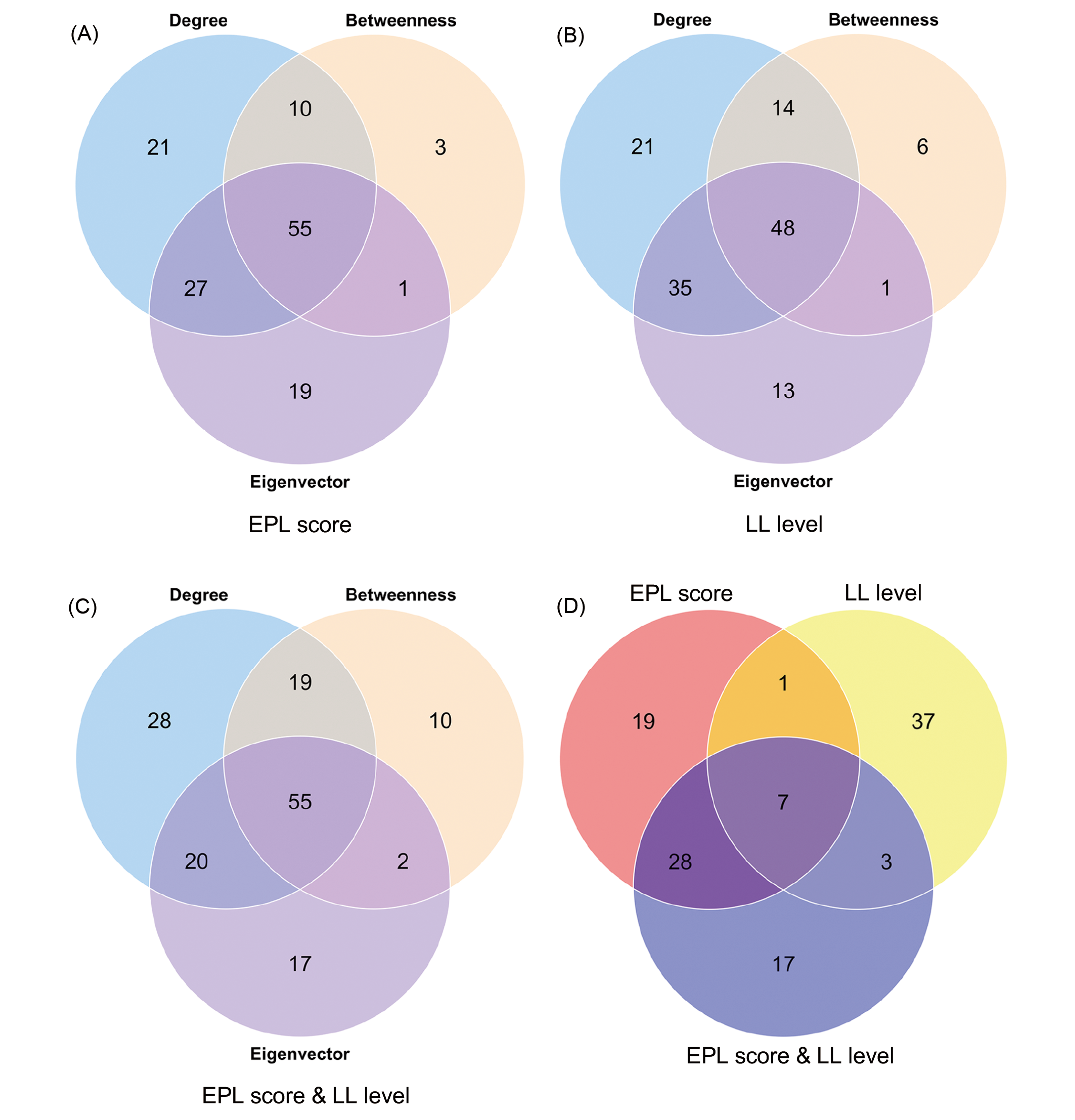


**Figure S5. Venn diagram showing key-node genes identified in this study.** (A) The 55 key-node genes in the grouping method of EPL scores. (B) The 48 key-node genes in the grouping method of LL levels. (C) The 55 key-node genes in the grouping method based on both EPL scores and LL levels. (D) The 7 common genes among the three grouping methods.


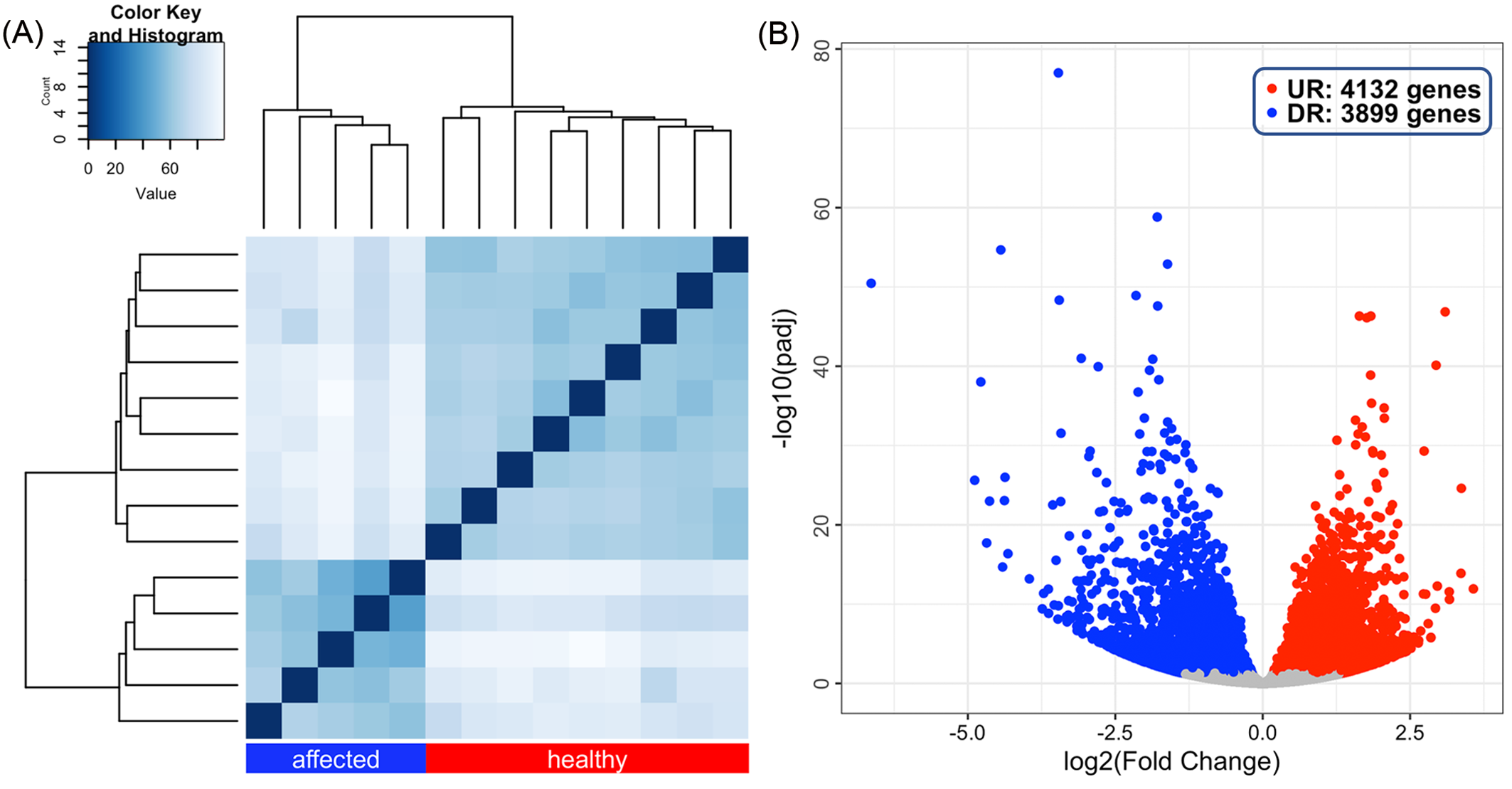


**Figure S6. Differential gene expression in healthy lung tissues and affected lung lesions.** The 14 lung tissues (five from affected lung lesions, nine from healthy lung parts) were sampled from a commercial black pig herd. (A) Hierarchical cluster plot showed that the transcriptomic profiles of healthy and affected lung samples were divergent to each other, and healthy and affected lung samples were grouped into their own branches. **(**B) The log2 of fold change between healthy and affected lung tissues is represented on the x-axis and the negative log of *P*-values from the t-test is represented on the y-axis. *Red* and *blue* dots indicate 4,132 genes and 3,899 genes that are significantly upregulated (UR) and downregulated (DR) in the healthy lung samples compared to the affected samples (adjusted *P* value < 0.05), respectively. *Gray* dots indicate genes with no significant difference.

| **Table S1**. The lung tissue samples and the derived individuals used in RNA-seq analysis. | | | |
| --- | --- | --- | --- |
| No | Derived pig ID | Healthy part | Infected part |
| 1 | SX1904 | 0 | 1 |
| 2 | SX1901 | 0 | 2 |
| 3 | SX2907 | 1 | 1 |
| 4 | SX6901 | 1 | 1 |
| 5 | SX5201 | 1 | 0 |
| 6 | SX4709 | 1 | 0 |
| 7 | SX0510 | 1 | 0 |
| 8 | SX4705 | 1 | 0 |
| 9 | SX5312 | 1 | 0 |
| 10 | SX5203 | 1 | 0 |
| 11 | SX0102 | 1 | 0 |
|  | In total | 9 | 5 |

| **Table S2**. Description statistics of EPL scores, LL levels and body weights of Bamaxing pigs at the ages of 150, 180, 240 and 300 days. | | | | | | |
| --- | --- | --- | --- | --- | --- | --- |
|  | EPL score | LL level | BodyWt_D150 (kg) | BodyWt_D180 (kg) | BodyWt_D240 (kg) | BodyWt_D300 (kg) |
| Min | 0 | 0 | 11.84 | 17.07 | 27.23 | 36.74 |
| Mean | 22.3 ± 22.3 | 0.282 ± 0.251 | 21.65 ± 4.25 | 28.32 ± 5.40 | 46.66 ± 7.74 | 60.00 ± 9.65 |
| Max | 116 | 0.993 | 36.24 | 48.59 | 71.68 | 84.73 |

| **Table S3**. Candidate genes surpassing the significant thresholds in the EPL, LL and EPL_LL groups | | | | |
| --- | --- | --- | --- | --- |
| **Chromosome** | **Start** | **End** | **F_ST_** | **Gene** |
| **EPL scores** | | | | |
| 1 | 20425000 | 20475000 | 0.0285 | *SAMD5* |
| 1 | 20550000 | 20600000 | 0.0409 | *STXBP5* |
| 1 | 49775000 | 49825000 | 0.0295 | *ROS1* |
| 1 | 49800000 | 49850000 | 0.0401 | *ROS1* |
| 1 | 50475000 | 50525000 | 0.0639 | *PTP4A1* |
| 1 | 50500000 | 50550000 | 0.0664 | *PTP4A1* |
| 1 | 50850000 | 50900000 | 0.0374 | *EYS* |
| 1 | 50875000 | 50925000 | 0.0351 | *EYS* |
| 1 | 59650000 | 59700000 | 0.0507 | *KIAA1009* |
| 1 | 59675000 | 59725000 | 0.0512 | *KIAA1009* |
| 1 | 59700000 | 59750000 | 0.0313 | *KIAA1009* |
| 1 | 62750000 | 62800000 | 0.0723 | *AKIRIN2* |
| 1 | 62750000 | 62800000 | 0.0723 | *ORC3L* |
| 1 | 62775000 | 62825000 | 0.0678 | *AKIRIN2* |
| 1 | 62775000 | 62825000 | 0.0678 | *ORC3L* |
| 1 | 80700000 | 80750000 | 0.0373 | *POPDC3* |
| 1 | 82900000 | 82950000 | 0.0285 | *SOBP* |
| 1 | 83075000 | 83125000 | 0.0289 | *SCML4* |
| 1 | 86375000 | 86425000 | 0.0302 | *AMD1* |
| 1 | 86400000 | 86450000 | 0.0290 | *AMD1* |
| 1 | 86425000 | 86475000 | 0.0398 | *CDK19* |
| 1 | 86450000 | 86500000 | 0.0441 | *CDK19* |
| 1 | 94525000 | 94575000 | 0.0321 | *IBTK* |
| 1 | 94550000 | 94600000 | 0.0307 | *IBTK* |
| 1 | 103250000 | 103300000 | 0.0315 | *CD109* |
| 1 | 104225000 | 104275000 | 0.0401 | *SETBP1* |
| 1 | 141675000 | 141725000 | 0.0296 | *EIF3J* |
| 1 | 141675000 | 141725000 | 0.0296 | *SPG11* |
| 1 | 141725000 | 141775000 | 0.0290 | *CTDSPL* |
| 1 | 141725000 | 141775000 | 0.0290 | *CTDSPL2* |
| 1 | 141725000 | 141775000 | 0.0290 | *EIF3J* |
| 1 | 141750000 | 141800000 | 0.0303 | *CTDSPL* |
| 1 | 141750000 | 141800000 | 0.0303 | *CTDSPL2* |
| 1 | 141775000 | 141825000 | 0.0375 | *CASC4* |
| 1 | 141775000 | 141825000 | 0.0375 | *CTDSPL* |
| 1 | 141775000 | 141825000 | 0.0375 | *CTDSPL2* |
| 1 | 141800000 | 141850000 | 0.0406 | *CASC4* |
| 1 | 141850000 | 141900000 | 0.0409 | *CTDSPL* |
| 1 | 141850000 | 141900000 | 0.0409 | *CTDSPL2* |
| 1 | 141950000 | 142000000 | 0.0370 | *CASC4* |
| 1 | 153450000 | 153500000 | 0.0399 | *PGPEP1L* |
| 1 | 155250000 | 155300000 | 0.0803 | *CERS3* |
| 1 | 155275000 | 155325000 | 0.0684 | *CERS3* |
| 1 | 155300000 | 155350000 | 0.0854 | *CERS3* |
| 1 | 155325000 | 155375000 | 0.0690 | *LINS1* |
| 1 | 155375000 | 155425000 | 0.0710 | *ASB7* |
| 1 | 155375000 | 155425000 | 0.0710 | *LINS1* |
| 1 | 155400000 | 155450000 | 0.0597 | *ASB7* |
| 1 | 155425000 | 155475000 | 0.0484 | *ASB7* |
| 1 | 156050000 | 156100000 | 0.0364 | *GABRG3* |
| 1 | 156050000 | 156100000 | 0.0364 | *SELS* |
| 1 | 156075000 | 156125000 | 0.0359 | *GABRG3* |
| 1 | 179975000 | 180025000 | 0.0313 | *NEDD4L* |
| 1 | 180025000 | 180075000 | 0.0426 | *NEDD4L* |
| 1 | 185350000 | 185400000 | 0.0289 | *RPLP1* |
| 1 | 185925000 | 185975000 | 0.0418 | *TLE3* |
| 1 | 185950000 | 186000000 | 0.0538 | *TLE3* |
| 1 | 205925000 | 205975000 | 0.0324 | *PELI2* |
| 1 | 216250000 | 216300000 | 0.0530 | *SGPP1* |
| 1 | 216275000 | 216325000 | 0.0440 | *SGPP1* |
| 1 | 216325000 | 216375000 | 0.0296 | *SGPP1* |
| 1 | 216550000 | 216600000 | 0.0929 | *PPP2R5E* |
| 1 | 216575000 | 216625000 | 0.0676 | *PPP2R5E* |
| 1 | 216600000 | 216650000 | 0.0342 | *PPP2R5E* |
| 1 | 216750000 | 216800000 | 0.0568 | *RHOJ* |
| 1 | 216775000 | 216825000 | 0.0446 | *GPHB5* |
| 1 | 216775000 | 216825000 | 0.0446 | *RHOJ* |
| 1 | 256525000 | 256575000 | 0.0289 | *PRUNE2* |
| 1 | 256625000 | 256675000 | 0.0327 | *FOXB2* |
| 1 | 275550000 | 275600000 | 0.0308 | *OR13D1* |
| 1 | 275575000 | 275625000 | 0.0348 | *OR13D1* |
| 1 | 275800000 | 275850000 | 0.0301 | *ABCA1* |
| 1 | 288000000 | 288050000 | 0.0291 | *PAPPA* |
| 1 | 288000000 | 288050000 | 0.0291 | *PAPPAS* |
| 1 | 312975000 | 313025000 | 0.0311 | *OR4A16* |
| 2 | 7625000 | 7675000 | 0.0384 | *ATL3* |
| 2 | 7625000 | 7675000 | 0.0384 | *HRASLS2* |
| 2 | 7625000 | 7675000 | 0.0384 | *PLA2G16* |
| 2 | 7650000 | 7700000 | 0.0397 | *HRASLS2* |
| 2 | 7650000 | 7700000 | 0.0397 | *PLA2G16* |
| 2 | 7675000 | 7725000 | 0.0284 | *ATL3* |
| 2 | 7675000 | 7725000 | 0.0284 | *HRASLS2* |
| 2 | 7675000 | 7725000 | 0.0284 | *LGALS12* |
| 2 | 7675000 | 7725000 | 0.0284 | *PLA2G16* |
| 2 | 7675000 | 7725000 | 0.0284 | *RTN3* |
| 2 | 26675000 | 26725000 | 0.0296 | *RAG2* |
| 2 | 26750000 | 26800000 | 0.0287 | *TRAF6* |
| 2 | 26800000 | 26850000 | 0.0292 | *PRR5L* |
| 2 | 27075000 | 27125000 | 0.0301 | *COMMD9* |
| 2 | 27450000 | 27500000 | 0.0366 | *LDLRAD3* |
| 2 | 28075000 | 28125000 | 0.0317 | *SLC1A2* |
| 2 | 28100000 | 28150000 | 0.0372 | *SLC1A2* |
| 2 | 28125000 | 28175000 | 0.0327 | *CD44* |
| 2 | 28125000 | 28175000 | 0.0327 | *SLC1A2* |
| 2 | 28175000 | 28225000 | 0.0294 | *CD44* |
| 2 | 28350000 | 28400000 | 0.0438 | *PDHX* |
| 2 | 28375000 | 28425000 | 0.0341 | *PDHX* |
| 2 | 30000000 | 30050000 | 0.0393 | *HIPK3* |
| 2 | 30025000 | 30075000 | 0.0301 | *HIPK3* |
| 2 | 30325000 | 30375000 | 0.0360 | *CSTF3* |
| 2 | 30325000 | 30375000 | 0.0360 | *DEPDC7* |
| 2 | 30325000 | 30375000 | 0.0360 | *TCP11L1* |
| 2 | 30350000 | 30400000 | 0.0312 | *TCP11L1* |
| 2 | 30475000 | 30525000 | 0.0484 | *QSER1* |
| 2 | 30525000 | 30575000 | 0.0571 | *PRRG4* |
| 2 | 30550000 | 30600000 | 0.0591 | *PRRG4* |
| 2 | 30575000 | 30625000 | 0.0702 | *CCDC73* |
| 2 | 30600000 | 30650000 | 0.0666 | *CCDC73* |
| 2 | 30625000 | 30675000 | 0.0708 | *CCDC73* |
| 2 | 30650000 | 30700000 | 0.0606 | *CCDC73* |
| 2 | 30675000 | 30725000 | 0.0566 | *CCDC73* |
| 2 | 30700000 | 30750000 | 0.0780 | *CCDC73* |
| 2 | 30700000 | 30750000 | 0.0780 | *EIF3M* |
| 2 | 30725000 | 30775000 | 0.0642 | *CCDC73* |
| 2 | 30725000 | 30775000 | 0.0642 | *EIF3M* |
| 2 | 30750000 | 30800000 | 0.0518 | *EIF3M* |
| 2 | 30875000 | 30925000 | 0.0332 | *WT1* |
| 2 | 30900000 | 30950000 | 0.0287 | *WT1* |
| 2 | 32600000 | 32650000 | 0.0291 | *MPPED2* |
| 2 | 32825000 | 32875000 | 0.0745 | *FSHB* |
| 2 | 32850000 | 32900000 | 0.0615 | *FSHB* |
| 2 | 34625000 | 34675000 | 0.0430 | *METT5D1* |
| 2 | 34650000 | 34700000 | 0.0375 | *METT5D1* |
| 2 | 34775000 | 34825000 | 0.0344 | *METT5D1* |
| 2 | 34850000 | 34900000 | 0.0398 | *KIF18A* |
| 2 | 34875000 | 34925000 | 0.0350 | *KIF18A* |
| 2 | 35175000 | 35225000 | 0.0432 | *BDNF* |
| 2 | 35200000 | 35250000 | 0.0512 | *BDNF* |
| 2 | 35225000 | 35275000 | 0.0301 | *BDNF* |
| 2 | 35400000 | 35450000 | 0.0413 | *LIN7C* |
| 2 | 35425000 | 35475000 | 0.0706 | *LGR4* |
| 2 | 35425000 | 35475000 | 0.0706 | *LIN7C* |
| 2 | 35450000 | 35500000 | 0.0704 | *LGR4* |
| 2 | 35475000 | 35525000 | 0.0398 | *LGR4* |
| 2 | 35550000 | 35600000 | 0.0381 | *LGR4* |
| 2 | 35575000 | 35625000 | 0.0375 | *CCDC34* |
| 2 | 35575000 | 35625000 | 0.0375 | *LGR4* |
| 2 | 35600000 | 35650000 | 0.0301 | *CCDC34* |
| 2 | 35600000 | 35650000 | 0.0301 | *LGR4* |
| 2 | 35625000 | 35675000 | 0.0326 | *CCDC34* |
| 2 | 35650000 | 35700000 | 0.0293 | *CCDC34* |
| 2 | 36225000 | 36275000 | 0.0291 | *ANO3* |
| 2 | 36275000 | 36325000 | 0.0288 | *ANO3* |
| 2 | 36275000 | 36325000 | 0.0288 | *MUC15* |
| 2 | 36300000 | 36350000 | 0.0451 | *ANO3* |
| 2 | 36300000 | 36350000 | 0.0451 | *MUC15* |
| 2 | 37600000 | 37650000 | 0.0368 | *LUZP2* |
| 2 | 37625000 | 37675000 | 0.0294 | *LUZP2* |
| 2 | 37875000 | 37925000 | 0.0301 | *LUZP2* |
| 2 | 37900000 | 37950000 | 0.0331 | *LUZP2* |
| 2 | 38250000 | 38300000 | 0.0316 | *LUZP2* |
| 2 | 39825000 | 39875000 | 0.0322 | *GAS2* |
| 2 | 39850000 | 39900000 | 0.0372 | *GAS2* |
| 2 | 39875000 | 39925000 | 0.0331 | *GAS2* |
| 2 | 40000000 | 40050000 | 0.0303 | *GAS2* |
| 2 | 40025000 | 40075000 | 0.0337 | *GAS2* |
| 2 | 40050000 | 40100000 | 0.0390 | *FANCF* |
| 2 | 40050000 | 40100000 | 0.0390 | *GAS2* |
| 2 | 40075000 | 40125000 | 0.0322 | *FANCF* |
| 2 | 40350000 | 40400000 | 0.0317 | *ANO5* |
| 2 | 41700000 | 41750000 | 0.0291 | *NELL1* |
| 2 | 44225000 | 44275000 | 0.0358 | *SERGEF* |
| 2 | 44275000 | 44325000 | 0.0407 | *SERGEF* |
| 2 | 44375000 | 44425000 | 0.0286 | *SERGEF* |
| 2 | 44400000 | 44450000 | 0.0320 | *KCNC1* |
| 2 | 44400000 | 44450000 | 0.0320 | *SERGEF* |
| 2 | 44600000 | 44650000 | 0.0317 | *USH1C* |
| 2 | 45225000 | 45275000 | 0.0331 | *PLEKHA7* |
| 2 | 45400000 | 45450000 | 0.0326 | *C11ORF58* |
| 2 | 45400000 | 45450000 | 0.0326 | *C6* |
| 2 | 45400000 | 45450000 | 0.0326 | *LOC645086* |
| 2 | 45400000 | 45450000 | 0.0326 | *PLEKHA7* |
| 2 | 45425000 | 45475000 | 0.0304 | *C11ORF58* |
| 2 | 45425000 | 45475000 | 0.0304 | *C6* |
| 2 | 45425000 | 45475000 | 0.0304 | *LOC645086* |
| 2 | 45600000 | 45650000 | 0.0478 | *SOX6* |
| 2 | 45625000 | 45675000 | 0.0463 | *SOX6* |
| 2 | 72850000 | 72900000 | 0.0346 | *VAV1* |
| 2 | 76375000 | 76425000 | 0.0294 | *SGTA* |
| 2 | 76375000 | 76425000 | 0.0294 | *SLC39A3* |
| 2 | 76375000 | 76425000 | 0.0294 | *THOP1* |
| 2 | 79200000 | 79250000 | 0.0330 | *THEG* |
| 2 | 79225000 | 79275000 | 0.0354 | *MIER2* |
| 2 | 79225000 | 79275000 | 0.0354 | *THEG* |
| 2 | 79250000 | 79300000 | 0.0493 | *MIER2* |
| 2 | 79250000 | 79300000 | 0.0493 | *PPAP2C* |
| 2 | 79275000 | 79325000 | 0.0293 | *MIER2* |
| 2 | 79275000 | 79325000 | 0.0293 | *PPAP2C* |
| 2 | 79375000 | 79425000 | 0.0364 | *FLT4* |
| 2 | 86950000 | 87000000 | 0.0326 | *IQGAP2* |
| 2 | 90500000 | 90550000 | 0.0293 | *THBS4* |
| 2 | 90525000 | 90575000 | 0.0346 | *THBS4* |
| 3 | 1925000 | 1975000 | 0.0308 | *GNA12* |
| 3 | 1950000 | 2000000 | 0.0290 | *GNA12* |
| 3 | 2075000 | 2125000 | 0.0285 | *C7ORF27* |
| 3 | 2075000 | 2125000 | 0.0285 | *IQCE* |
| 3 | 2100000 | 2150000 | 0.0289 | *C7ORF27* |
| 3 | 2100000 | 2150000 | 0.0289 | *IQCE* |
| 3 | 101050000 | 101100000 | 0.0314 | *SRBD1* |
| 3 | 126225000 | 126275000 | 0.0332 | *MATN3* |
| 3 | 126225000 | 126275000 | 0.0332 | *WDR35* |
| 4 | 121225000 | 121275000 | 0.0313 | *PSMA5* |
| 4 | 121225000 | 121275000 | 0.0313 | *SORT1* |
| 4 | 121250000 | 121300000 | 0.0312 | *CELSR2* |
| 4 | 121250000 | 121300000 | 0.0312 | *PSRC1* |
| 4 | 121250000 | 121300000 | 0.0312 | *SORT1* |
| 4 | 137125000 | 137175000 | 0.0370 | *HFM1* |
| 4 | 137150000 | 137200000 | 0.0508 | *HFM1* |
| 4 | 137175000 | 137225000 | 0.0359 | *HFM1* |
| 4 | 141325000 | 141375000 | 0.0327 | *LMO4* |
| 5 | 1025000 | 1075000 | 0.0364 | *ATXN10* |
| 5 | 1025000 | 1075000 | 0.0364 | *FBLN1* |
| 5 | 1050000 | 1100000 | 0.0360 | *FBLN1* |
| 5 | 1100000 | 1150000 | 0.0339 | *FBLN1* |
| 5 | 8650000 | 8700000 | 0.0288 | *CACNG2* |
| 5 | 36125000 | 36175000 | 0.0347 | *CPSF6* |
| 5 | 36125000 | 36175000 | 0.0347 | *YEATS4* |
| 5 | 36150000 | 36200000 | 0.0406 | *CPSF6* |
| 5 | 36150000 | 36200000 | 0.0406 | *LYZ* |
| 5 | 36150000 | 36200000 | 0.0406 | *YEATS4* |
| 5 | 36175000 | 36225000 | 0.0300 | *LYZ* |
| 5 | 36175000 | 36225000 | 0.0300 | *YEATS4* |
| 5 | 55750000 | 55800000 | 0.0286 | *PDE3A* |
| 6 | 32175000 | 32225000 | 0.0311 | *PHKB* |
| 6 | 33075000 | 33125000 | 0.0284 | *C16ORF87* |
| 6 | 33075000 | 33125000 | 0.0284 | *F8* |
| 6 | 38850000 | 38900000 | 0.0299 | *PEPD* |
| 6 | 39450000 | 39500000 | 0.0319 | *KIAA0355* |
| 6 | 39450000 | 39500000 | 0.0319 | *PDCD2L* |
| 6 | 39475000 | 39525000 | 0.0330 | *GPI* |
| 6 | 39475000 | 39525000 | 0.0330 | *PDCD2L* |
| 6 | 39925000 | 39975000 | 0.0293 | *ZNF599* |
| 6 | 39925000 | 39975000 | 0.0293 | *ZNF792* |
| 6 | 40300000 | 40350000 | 0.0308 | *CD22* |
| 6 | 40300000 | 40350000 | 0.0308 | *FFAR1* |
| 6 | 40300000 | 40350000 | 0.0308 | *FFAR3* |
| 6 | 40325000 | 40375000 | 0.0290 | *CD22* |
| 6 | 40325000 | 40375000 | 0.0290 | *FFAR1* |
| 6 | 40325000 | 40375000 | 0.0290 | *FFAR3* |
| 6 | 40325000 | 40375000 | 0.0290 | *GAPDHS* |
| 6 | 40325000 | 40375000 | 0.0290 | *MAG* |
| 6 | 40400000 | 40450000 | 0.0297 | *ATP4A* |
| 6 | 40425000 | 40475000 | 0.0304 | *ATP4A* |
| 6 | 40600000 | 40650000 | 0.0317 | *ARHGAP33* |
| 6 | 40600000 | 40650000 | 0.0317 | *C19ORF55* |
| 6 | 40600000 | 40650000 | 0.0317 | *HSPB6* |
| 6 | 40600000 | 40650000 | 0.0317 | *LIN37* |
| 6 | 40600000 | 40650000 | 0.0317 | *MLL* |
| 6 | 40600000 | 40650000 | 0.0317 | *MLL4* |
| 6 | 40600000 | 40650000 | 0.0317 | *NPHS1* |
| 6 | 40600000 | 40650000 | 0.0317 | *PSENEN* |
| 6 | 40600000 | 40650000 | 0.0317 | *TMEM149* |
| 6 | 40600000 | 40650000 | 0.0317 | *U2AF1L4* |
| 6 | 40600000 | 40650000 | 0.0317 | *ZBTB32* |
| 6 | 40975000 | 41025000 | 0.0329 | *ZNF146* |
| 6 | 40975000 | 41025000 | 0.0329 | *ZNF26* |
| 6 | 40975000 | 41025000 | 0.0329 | *ZNF260* |
| 6 | 41175000 | 41225000 | 0.0289 | *F8* |
| 6 | 41175000 | 41225000 | 0.0289 | *ZNF345* |
| 6 | 41175000 | 41225000 | 0.0289 | *ZNF382* |
| 6 | 41175000 | 41225000 | 0.0289 | *ZNF461* |
| 6 | 41175000 | 41225000 | 0.0289 | *ZNF829* |
| 6 | 42175000 | 42225000 | 0.0349 | *ZFP3* |
| 6 | 42175000 | 42225000 | 0.0349 | *ZFP30* |
| 6 | 42175000 | 42225000 | 0.0349 | *ZNF790* |
| 6 | 42225000 | 42275000 | 0.0292 | *ZNF607* |
| 6 | 42500000 | 42550000 | 0.0297 | *SIPA1L3* |
| 6 | 43775000 | 43825000 | 0.0442 | *DLL3* |
| 6 | 43825000 | 43875000 | 0.0292 | *SUPT5H* |
| 6 | 43825000 | 43875000 | 0.0292 | *TIMM50* |
| 6 | 43850000 | 43900000 | 0.0338 | *SUPT5H* |
| 6 | 43900000 | 43950000 | 0.0334 | *CLC* |
| 6 | 43900000 | 43950000 | 0.0334 | *LGALS13* |
| 6 | 43900000 | 43950000 | 0.0334 | *LGALS14* |
| 6 | 43925000 | 43975000 | 0.0298 | *CLC* |
| 6 | 43925000 | 43975000 | 0.0298 | *EID2* |
| 6 | 43925000 | 43975000 | 0.0298 | *EID2B* |
| 6 | 43925000 | 43975000 | 0.0298 | *LGALS13* |
| 6 | 43925000 | 43975000 | 0.0298 | *LGALS14* |
| 6 | 44075000 | 44125000 | 0.0451 | *CGB* |
| 6 | 44075000 | 44125000 | 0.0451 | *FCGBP* |
| 6 | 44100000 | 44150000 | 0.0309 | *CGB* |
| 6 | 44100000 | 44150000 | 0.0309 | *FCGBP* |
| 6 | 44225000 | 44275000 | 0.0287 | *ZNF780B* |
| 6 | 44575000 | 44625000 | 0.0351 | *ADCK4* |
| 6 | 44575000 | 44625000 | 0.0351 | *LTBP4* |
| 6 | 44600000 | 44650000 | 0.0365 | *ADCK4* |
| 6 | 44600000 | 44650000 | 0.0365 | *C19ORF54* |
| 6 | 44600000 | 44650000 | 0.0365 | *ITPKC* |
| 6 | 44600000 | 44650000 | 0.0365 | *LTBP4* |
| 6 | 44625000 | 44675000 | 0.0353 | *C19ORF54* |
| 6 | 44625000 | 44675000 | 0.0353 | *ITPKC* |
| 6 | 44625000 | 44675000 | 0.0353 | *MIA* |
| 6 | 44625000 | 44675000 | 0.0353 | *RAB4B* |
| 6 | 44625000 | 44675000 | 0.0353 | *SNRPA* |
| 6 | 44650000 | 44700000 | 0.0421 | *C19ORF54* |
| 6 | 44650000 | 44700000 | 0.0421 | *EGLN2* |
| 6 | 44650000 | 44700000 | 0.0421 | *MIA* |
| 6 | 44650000 | 44700000 | 0.0421 | *RAB4B* |
| 6 | 44650000 | 44700000 | 0.0421 | *SNRPA* |
| 6 | 44675000 | 44725000 | 0.0374 | *ADCK4* |
| 6 | 44675000 | 44725000 | 0.0374 | *CYP2F1* |
| 6 | 44675000 | 44725000 | 0.0374 | *EGLN2* |
| 6 | 44675000 | 44725000 | 0.0374 | *NUMBL* |
| 6 | 44675000 | 44725000 | 0.0374 | *RAB4B* |
| 6 | 44700000 | 44750000 | 0.0344 | *ADCK4* |
| 6 | 44700000 | 44750000 | 0.0344 | *CYP2F1* |
| 6 | 44700000 | 44750000 | 0.0344 | *NUMBL* |
| 6 | 44725000 | 44775000 | 0.0407 | *NUMBL* |
| 6 | 44750000 | 44800000 | 0.0398 | *LTBP4* |
| 6 | 44750000 | 44800000 | 0.0398 | *SHKBP1* |
| 6 | 44875000 | 44925000 | 0.0361 | *CYP2A13* |
| 6 | 44875000 | 44925000 | 0.0361 | *CYP2A6* |
| 6 | 44875000 | 44925000 | 0.0361 | *CYP2A7* |
| 6 | 44875000 | 44925000 | 0.0361 | *CYP2F1* |
| 6 | 44900000 | 44950000 | 0.0470 | *CYP2B6* |
| 6 | 44900000 | 44950000 | 0.0470 | *CYP2F1* |
| 6 | 44900000 | 44950000 | 0.0470 | *CYP2S1* |
| 6 | 44925000 | 44975000 | 0.0316 | *CYP2B6* |
| 6 | 44925000 | 44975000 | 0.0316 | *CYP2F1* |
| 6 | 44925000 | 44975000 | 0.0316 | *CYP2S1* |
| 6 | 44975000 | 45025000 | 0.0296 | *AXL* |
| 6 | 44975000 | 45025000 | 0.0296 | *CYP2F1* |
| 6 | 45025000 | 45075000 | 0.0331 | *TGFB1* |
| 6 | 45050000 | 45100000 | 0.0322 | *HNRNPUL1* |
| 6 | 45050000 | 45100000 | 0.0322 | *TGFB1* |
| 6 | 45075000 | 45125000 | 0.0342 | *CCDC9* |
| 6 | 45075000 | 45125000 | 0.0342 | *CCDC97* |
| 6 | 45075000 | 45125000 | 0.0342 | *HNRNPUL1* |
| 6 | 45425000 | 45475000 | 0.0313 | *CNFN* |
| 6 | 45425000 | 45475000 | 0.0313 | *F8* |
| 6 | 45425000 | 45475000 | 0.0313 | *LIPE* |
| 6 | 45425000 | 45475000 | 0.0313 | *MEGF8* |
| 6 | 45450000 | 45500000 | 0.0324 | *CNFN* |
| 6 | 45450000 | 45500000 | 0.0324 | *F8* |
| 6 | 45450000 | 45500000 | 0.0324 | *LIPE* |
| 6 | 45450000 | 45500000 | 0.0324 | *MEGF8* |
| 6 | 45500000 | 45550000 | 0.0351 | *CIC* |
| 6 | 45500000 | 45550000 | 0.0351 | *F8* |
| 6 | 45500000 | 45550000 | 0.0351 | *MEGF8* |
| 6 | 45500000 | 45550000 | 0.0351 | *PAFAH1B3* |
| 6 | 45500000 | 45550000 | 0.0351 | *PRR19* |
| 6 | 45500000 | 45550000 | 0.0351 | *TMEM145* |
| 6 | 45525000 | 45575000 | 0.0434 | *CIC* |
| 6 | 45525000 | 45575000 | 0.0434 | *PAFAH1B3* |
| 6 | 45525000 | 45575000 | 0.0434 | *PRR19* |
| 6 | 45550000 | 45600000 | 0.0378 | *ERF* |
| 6 | 45575000 | 45625000 | 0.0414 | *ERF* |
| 6 | 45575000 | 45625000 | 0.0414 | *GSK3A* |
| 6 | 45575000 | 45625000 | 0.0414 | *ZNF526* |
| 6 | 45600000 | 45650000 | 0.0414 | *DEDD2* |
| 6 | 45600000 | 45650000 | 0.0414 | *ERF* |
| 6 | 45600000 | 45650000 | 0.0414 | *GSK3A* |
| 6 | 45600000 | 45650000 | 0.0414 | *ZNF526* |
| 6 | 45625000 | 45675000 | 0.0357 | *DEDD2* |
| 6 | 45850000 | 45900000 | 0.0316 | *GRIK5* |
| 6 | 45850000 | 45900000 | 0.0316 | *POU2F2* |
| 6 | 46125000 | 46175000 | 0.0346 | *ETHE1* |
| 6 | 46125000 | 46175000 | 0.0346 | *TEX101* |
| 6 | 46125000 | 46175000 | 0.0346 | *XRCC1* |
| 6 | 46125000 | 46175000 | 0.0346 | *ZNF575* |
| 6 | 46150000 | 46200000 | 0.0387 | *IRGQ* |
| 6 | 46150000 | 46200000 | 0.0387 | *XRCC1* |
| 6 | 46150000 | 46200000 | 0.0387 | *ZNF575* |
| 6 | 46150000 | 46200000 | 0.0387 | *ZNF576* |
| 6 | 46175000 | 46225000 | 0.0396 | *CADM4* |
| 6 | 46175000 | 46225000 | 0.0396 | *IRGQ* |
| 6 | 46175000 | 46225000 | 0.0396 | *SRRM5* |
| 6 | 46175000 | 46225000 | 0.0396 | *XRCC1* |
| 6 | 46175000 | 46225000 | 0.0396 | *ZNF428* |
| 6 | 46175000 | 46225000 | 0.0396 | *ZNF576* |
| 6 | 46200000 | 46250000 | 0.0299 | *CADM4* |
| 6 | 46200000 | 46250000 | 0.0299 | *ETHE1* |
| 6 | 46200000 | 46250000 | 0.0299 | *PHLDB3* |
| 6 | 46200000 | 46250000 | 0.0299 | *SRRM5* |
| 6 | 46200000 | 46250000 | 0.0299 | *ZNF428* |
| 6 | 46225000 | 46275000 | 0.0295 | *ETHE1* |
| 6 | 46225000 | 46275000 | 0.0295 | *LYPD3* |
| 6 | 46225000 | 46275000 | 0.0295 | *PHLDB3* |
| 6 | 46250000 | 46300000 | 0.0390 | *LYPD3* |
| 6 | 46250000 | 46300000 | 0.0390 | *PHLDB3* |
| 6 | 46325000 | 46375000 | 0.0371 | *PLAUR* |
| 6 | 46350000 | 46400000 | 0.0385 | *PLAUR* |
| 6 | 47200000 | 47250000 | 0.0285 | *BCAM* |
| 6 | 47200000 | 47250000 | 0.0285 | *PVRL2* |
| 6 | 47225000 | 47275000 | 0.0307 | *APOE* |
| 6 | 47225000 | 47275000 | 0.0307 | *LOC100129500* |
| 6 | 47225000 | 47275000 | 0.0307 | *PVRL2* |
| 6 | 47225000 | 47275000 | 0.0307 | *TOMM40* |
| 6 | 47275000 | 47325000 | 0.0364 | *APOC1* |
| 6 | 47275000 | 47325000 | 0.0364 | *APOC2* |
| 6 | 47275000 | 47325000 | 0.0364 | *APOC4* |
| 6 | 47275000 | 47325000 | 0.0364 | *CLPTM1* |
| 6 | 47275000 | 47325000 | 0.0364 | *TOMM40* |
| 6 | 47300000 | 47350000 | 0.0359 | *CLPTM1* |
| 6 | 47300000 | 47350000 | 0.0359 | *PVRL2* |
| 6 | 47300000 | 47350000 | 0.0359 | *RELB* |
| 6 | 47300000 | 47350000 | 0.0359 | *TOMM40* |
| 6 | 47375000 | 47425000 | 0.0304 | *RSPH6A* |
| 6 | 47375000 | 47425000 | 0.0304 | *SYMPK* |
| 6 | 47400000 | 47450000 | 0.0416 | *FOXA3* |
| 6 | 47400000 | 47450000 | 0.0416 | *RSPH6A* |
| 6 | 47400000 | 47450000 | 0.0416 | *SYMPK* |
| 6 | 47425000 | 47475000 | 0.0406 | *FOXA3* |
| 6 | 47425000 | 47475000 | 0.0406 | *IRF2BP1* |
| 6 | 47425000 | 47475000 | 0.0406 | *MYPOP* |
| 6 | 47425000 | 47475000 | 0.0406 | *SYMPK* |
| 6 | 47450000 | 47500000 | 0.0351 | *FOXA3* |
| 6 | 47450000 | 47500000 | 0.0351 | *IRF2BP1* |
| 6 | 47450000 | 47500000 | 0.0351 | *MYPOP* |
| 6 | 47450000 | 47500000 | 0.0351 | *NANOS2* |
| 6 | 48275000 | 48325000 | 0.0309 | *ZC3H4* |
| 6 | 48350000 | 48400000 | 0.0338 | *SAE1* |
| 6 | 48375000 | 48425000 | 0.0294 | *BBC3* |
| 6 | 48375000 | 48425000 | 0.0294 | *SAE1* |
| 6 | 48400000 | 48450000 | 0.0313 | *BBC3* |
| 6 | 48400000 | 48450000 | 0.0313 | *CCDC9* |
| 6 | 48400000 | 48450000 | 0.0313 | *SAE1* |
| 6 | 48425000 | 48475000 | 0.0354 | *CCDC9* |
| 6 | 48425000 | 48475000 | 0.0354 | *PRR24* |
| 6 | 49225000 | 49275000 | 0.0284 | *LIG1* |
| 6 | 49400000 | 49450000 | 0.0290 | *SYNGR4* |
| 6 | 49400000 | 49450000 | 0.0290 | *TMEM143* |
| 6 | 50025000 | 50075000 | 0.0339 | *CGB* |
| 6 | 50025000 | 50075000 | 0.0339 | *CGB1* |
| 6 | 50025000 | 50075000 | 0.0339 | *CGB2* |
| 6 | 50025000 | 50075000 | 0.0339 | *CGB5* |
| 6 | 50025000 | 50075000 | 0.0339 | *CGB7* |
| 6 | 50025000 | 50075000 | 0.0339 | *CGB8* |
| 6 | 50025000 | 50075000 | 0.0339 | *KCNA7* |
| 6 | 50025000 | 50075000 | 0.0339 | *LHB* |
| 6 | 50025000 | 50075000 | 0.0339 | *NTF4* |
| 6 | 50025000 | 50075000 | 0.0339 | *RUVBL2* |
| 6 | 50025000 | 50075000 | 0.0339 | *SNRNP70* |
| 6 | 50050000 | 50100000 | 0.0311 | *CGB* |
| 6 | 50050000 | 50100000 | 0.0311 | *CGB1* |
| 6 | 50050000 | 50100000 | 0.0311 | *CGB2* |
| 6 | 50050000 | 50100000 | 0.0311 | *CGB5* |
| 6 | 50050000 | 50100000 | 0.0311 | *CGB7* |
| 6 | 50050000 | 50100000 | 0.0311 | *CGB8* |
| 6 | 50050000 | 50100000 | 0.0311 | *FTL* |
| 6 | 50050000 | 50100000 | 0.0311 | *FTLP2* |
| 6 | 50050000 | 50100000 | 0.0311 | *GYS1* |
| 6 | 50050000 | 50100000 | 0.0311 | *LHB* |
| 6 | 50050000 | 50100000 | 0.0311 | *NTF4* |
| 6 | 50050000 | 50100000 | 0.0311 | *RUVBL2* |
| 6 | 57625000 | 57675000 | 0.0347 | *A1BG* |
| 6 | 57625000 | 57675000 | 0.0347 | *F8* |
| 6 | 57625000 | 57675000 | 0.0347 | *ZNF837* |
| 6 | 57625000 | 57675000 | 0.0347 | *ZSCAN22* |
| 7 | 1650000 | 1700000 | 0.0360 | *MYLK4* |
| 7 | 91775000 | 91825000 | 0.0293 | *RGMA* |
| 7 | 91800000 | 91850000 | 0.0426 | *RGMA* |
| 7 | 91825000 | 91875000 | 0.0357 | *RGMA* |
| 7 | 103400000 | 103450000 | 0.0295 | *ABCD4* |
| 7 | 104100000 | 104150000 | 0.0306 | *ACYP1* |
| 7 | 104100000 | 104150000 | 0.0306 | *FAM164C* |
| 7 | 104100000 | 104150000 | 0.0306 | *NEK9* |
| 7 | 104750000 | 104800000 | 0.0291 | *TTLL5* |
| 7 | 104775000 | 104825000 | 0.0403 | *TTLL5* |
| 7 | 122425000 | 122475000 | 0.0323 | *PPP4R4* |
| 8 | 3750000 | 3800000 | 0.0424 | *SORCS2* |
| 8 | 3775000 | 3825000 | 0.0311 | *SORCS2* |
| 8 | 3925000 | 3975000 | 0.0300 | *SORCS2* |
| 8 | 5650000 | 5700000 | 0.0300 | *DRD5* |
| 8 | 5675000 | 5725000 | 0.0295 | *DRD5* |
| 8 | 5675000 | 5725000 | 0.0295 | *OTOP1* |
| 8 | 5725000 | 5775000 | 0.0317 | *OTOP1* |
| 8 | 5725000 | 5775000 | 0.0317 | *TMEM128* |
| 8 | 5750000 | 5800000 | 0.0286 | *LYAR* |
| 8 | 5750000 | 5800000 | 0.0286 | *TMEM128* |
| 8 | 5800000 | 5850000 | 0.0306 | *ZBTB49* |
| 8 | 5925000 | 5975000 | 0.0328 | *ZNF518B* |
| 8 | 6125000 | 6175000 | 0.0338 | *CLNK* |
| 8 | 12550000 | 12600000 | 0.0490 | *NCAPG* |
| 8 | 12575000 | 12625000 | 0.0409 | *LCORL* |
| 8 | 12575000 | 12625000 | 0.0409 | *NCAPG* |
| 8 | 12600000 | 12650000 | 0.0301 | *LCORL* |
| 8 | 12600000 | 12650000 | 0.0301 | *NCAPG* |
| 8 | 12625000 | 12675000 | 0.0326 | *LCORL* |
| 8 | 32375000 | 32425000 | 0.0314 | *C4ORF34* |
| 8 | 50025000 | 50075000 | 0.0766 | *ETFDH* |
| 8 | 50025000 | 50075000 | 0.0766 | *PPID* |
| 8 | 50050000 | 50100000 | 0.0542 | *ETFDH* |
| 8 | 50050000 | 50100000 | 0.0542 | *PPID* |
| 8 | 53750000 | 53800000 | 0.0428 | *FSTL5* |
| 8 | 53775000 | 53825000 | 0.0377 | *FSTL5* |
| 8 | 53850000 | 53900000 | 0.0382 | *FSTL5* |
| 8 | 53875000 | 53925000 | 0.0378 | *FSTL5* |
| 8 | 58300000 | 58350000 | 0.0303 | *CEP135* |
| 8 | 58300000 | 58350000 | 0.0303 | *EXOC1* |
| 8 | 74200000 | 74250000 | 0.0286 | *CXCL1* |
| 8 | 74250000 | 74300000 | 0.0293 | *CXCL3* |
| 8 | 83400000 | 83450000 | 0.0393 | *LRBA* |
| 8 | 83425000 | 83475000 | 0.0291 | *LRBA* |
| 8 | 89500000 | 89550000 | 0.0372 | *GAB1* |
| 8 | 89775000 | 89825000 | 0.0327 | *USP38* |
| 8 | 110275000 | 110325000 | 0.0288 | *TNIP3* |
| 8 | 110625000 | 110675000 | 0.0342 | *PRDM5* |
| 8 | 110700000 | 110750000 | 0.0286 | *PRDM5* |
| 8 | 116050000 | 116100000 | 0.0324 | *NDST4* |
| 8 | 116075000 | 116125000 | 0.0386 | *NDST4* |
| 9 | 22500000 | 22550000 | 0.0348 | *CCDC81* |
| 9 | 22525000 | 22575000 | 0.0554 | *CCDC81* |
| 9 | 22550000 | 22600000 | 0.0412 | *CCDC81* |
| 9 | 22550000 | 22600000 | 0.0412 | *ME3* |
| 9 | 50775000 | 50825000 | 0.0403 | *ARCN1* |
| 9 | 50800000 | 50850000 | 0.0385 | *ARCN1* |
| 9 | 50800000 | 50850000 | 0.0385 | *C11ORF60* |
| 9 | 50900000 | 50950000 | 0.0287 | *MLL* |
| 9 | 50900000 | 50950000 | 0.0287 | *PHLDB1* |
| 9 | 70150000 | 70200000 | 0.0289 | *FMO1* |
| 9 | 82375000 | 82425000 | 0.0349 | *PON2* |
| 9 | 82375000 | 82425000 | 0.0349 | *PON3* |
| 9 | 83050000 | 83100000 | 0.0491 | *DYNC1I1* |
| 9 | 83075000 | 83125000 | 0.0349 | *DYNC1I1* |
| 9 | 93425000 | 93475000 | 0.0365 | *TMEM195* |
| 9 | 97000000 | 97050000 | 0.0284 | *HDAC9* |
| 9 | 97025000 | 97075000 | 0.0505 | *HDAC9* |
| 9 | 106375000 | 106425000 | 0.0361 | *SEMA3A* |
| 9 | 106400000 | 106450000 | 0.0320 | *SEMA3A* |
| 9 | 113700000 | 113750000 | 0.0402 | *FBXL13* |
| 10 | 19725000 | 19775000 | 0.0305 | *C1ORF101* |
| 10 | 19725000 | 19775000 | 0.0305 | *PPPDE1* |
| 10 | 19750000 | 19800000 | 0.0307 | *C1ORF101* |
| 10 | 26200000 | 26250000 | 0.0395 | *PTPRC* |
| 10 | 29850000 | 29900000 | 0.0300 | *SLC35D2* |
| 10 | 31350000 | 31400000 | 0.0303 | *C9ORF3* |
| 11 | 86225000 | 86275000 | 0.0340 | *MCF2L* |
| 11 | 86250000 | 86300000 | 0.0425 | *MCF2L* |
| 12 | 18250000 | 18300000 | 0.0357 | *ARL17A* |
| 12 | 18250000 | 18300000 | 0.0357 | *ARL17B* |
| 12 | 18250000 | 18300000 | 0.0357 | *C6* |
| 12 | 18250000 | 18300000 | 0.0357 | *LOC646030* |
| 12 | 18250000 | 18300000 | 0.0357 | *LRRC37A* |
| 12 | 18250000 | 18300000 | 0.0357 | *LRRC37B* |
| 12 | 32075000 | 32125000 | 0.0374 | *STXBP4* |
| 12 | 53225000 | 53275000 | 0.0316 | *WSCD1* |
| 12 | 53250000 | 53300000 | 0.0300 | *WSCD1* |
| 12 | 53875000 | 53925000 | 0.0438 | *ZFP3* |
| 12 | 53875000 | 53925000 | 0.0438 | *ZNF232* |
| 12 | 54025000 | 54075000 | 0.0299 | *ENO3* |
| 12 | 54025000 | 54075000 | 0.0299 | *GP1BA* |
| 12 | 54025000 | 54075000 | 0.0299 | *PFN1* |
| 12 | 54025000 | 54075000 | 0.0299 | *RNF167* |
| 12 | 54025000 | 54075000 | 0.0299 | *SLC25A11* |
| 12 | 54650000 | 54700000 | 0.0316 | *SLC16A11* |
| 12 | 54650000 | 54700000 | 0.0316 | *SLC16A13* |
| 13 | 16550000 | 16600000 | 0.0352 | *ZCWPW2* |
| 13 | 16575000 | 16625000 | 0.0292 | *ZCWPW2* |
| 13 | 17650000 | 17700000 | 0.0337 | *RBMS3* |
| 13 | 23375000 | 23425000 | 0.0363 | *STAC* |
| 13 | 23575000 | 23625000 | 0.0353 | *MLH1* |
| 13 | 24950000 | 25000000 | 0.0344 | *CTDSPL* |
| 13 | 24975000 | 25025000 | 0.0327 | *CTDSPL* |
| 13 | 27825000 | 27875000 | 0.0405 | *ULK4* |
| 13 | 28725000 | 28775000 | 0.0421 | *SEC22C* |
| 13 | 28725000 | 28775000 | 0.0421 | *VIPR1* |
| 13 | 28750000 | 28800000 | 0.0410 | *NKTR* |
| 13 | 28750000 | 28800000 | 0.0410 | *SEC22C* |
| 13 | 28750000 | 28800000 | 0.0410 | *SS18L2* |
| 13 | 29050000 | 29100000 | 0.0302 | *FAM198A* |
| 13 | 29050000 | 29100000 | 0.0302 | *LOC26172* |
| 13 | 32050000 | 32100000 | 0.0332 | *LZTFL1* |
| 13 | 33375000 | 33425000 | 0.0406 | *PTPN23* |
| 13 | 33800000 | 33850000 | 0.0359 | *SMARCC1* |
| 13 | 33825000 | 33875000 | 0.0372 | *SMARCC1* |
| 13 | 33850000 | 33900000 | 0.0452 | *DHX30* |
| 13 | 33850000 | 33900000 | 0.0452 | *SMARCC1* |
| 13 | 33875000 | 33925000 | 0.0387 | *DHX30* |
| 13 | 33875000 | 33925000 | 0.0387 | *MAP4* |
| 13 | 34650000 | 34700000 | 0.0290 | *IP6K2* |
| 13 | 34650000 | 34700000 | 0.0290 | *PRKAR2A* |
| 13 | 34675000 | 34725000 | 0.0302 | *PRKAR2A* |
| 13 | 36275000 | 36325000 | 0.0290 | *DOCK3* |
| 13 | 36275000 | 36325000 | 0.0290 | *MAPKAPK3* |
| 13 | 43250000 | 43300000 | 0.0335 | *DNAH12* |
| 13 | 70150000 | 70200000 | 0.0387 | *GRM7* |
| 13 | 106450000 | 106500000 | 0.0323 | *RSRC1* |
| 13 | 106550000 | 106600000 | 0.0412 | *RSRC1* |
| 13 | 137175000 | 137225000 | 0.0444 | *TMEM207* |
| 13 | 149675000 | 149725000 | 0.0288 | *GSK3B* |
| 13 | 149675000 | 149725000 | 0.0288 | *NR1I2* |
| 13 | 149925000 | 149975000 | 0.0410 | *PLA1A* |
| 13 | 149925000 | 149975000 | 0.0410 | *POPDC2* |
| 13 | 149950000 | 150000000 | 0.0305 | *PLA1A* |
| 13 | 149950000 | 150000000 | 0.0305 | *POPDC2* |
| 14 | 22625000 | 22675000 | 0.0360 | *ANXA10* |
| 14 | 23925000 | 23975000 | 0.0580 | *F8* |
| 14 | 23925000 | 23975000 | 0.0580 | *ZNF84* |
| 14 | 23950000 | 24000000 | 0.0315 | *F8* |
| 14 | 23950000 | 24000000 | 0.0315 | *ZNF26* |
| 14 | 23950000 | 24000000 | 0.0315 | *ZNF84* |
| 14 | 24025000 | 24075000 | 0.0314 | *CHFR* |
| 14 | 24025000 | 24075000 | 0.0314 | *ZNF605* |
| 14 | 25575000 | 25625000 | 0.0290 | *GPR133* |
| 14 | 25675000 | 25725000 | 0.0389 | *GPR133* |
| 14 | 25775000 | 25825000 | 0.0317 | *GPR133* |
| 14 | 27925000 | 27975000 | 0.0355 | *TMEM132C* |
| 14 | 27950000 | 28000000 | 0.0309 | *TMEM132C* |
| 14 | 28275000 | 28325000 | 0.0329 | *TMEM132C* |
| 14 | 31500000 | 31550000 | 0.0386 | *PITPNM2* |
| 14 | 31525000 | 31575000 | 0.0394 | *ARL6IP4* |
| 14 | 31525000 | 31575000 | 0.0394 | *PITPNM2* |
| 14 | 31625000 | 31675000 | 0.0316 | *VPS37B* |
| 14 | 31650000 | 31700000 | 0.0313 | *HIP1R* |
| 14 | 31650000 | 31700000 | 0.0313 | *VPS37B* |
| 14 | 34475000 | 34525000 | 0.0289 | *CUX2* |
| 14 | 36375000 | 36425000 | 0.0423 | *SUDS3* |
| 14 | 36400000 | 36450000 | 0.0451 | *SUDS3* |
| 14 | 36425000 | 36475000 | 0.0385 | *SUDS3* |
| 14 | 36800000 | 36850000 | 0.0318 | *KSR2* |
| 14 | 36800000 | 36850000 | 0.0318 | *RFC5* |
| 14 | 36825000 | 36875000 | 0.0493 | *KSR2* |
| 14 | 38225000 | 38275000 | 0.0335 | *MED13L* |
| 14 | 38250000 | 38300000 | 0.0385 | *MED13L* |
| 14 | 38300000 | 38350000 | 0.0312 | *MED13L* |
| 14 | 98475000 | 98525000 | 0.0332 | *LOC10431* |
| 14 | 98475000 | 98525000 | 0.0332 | *PARG* |
| 14 | 98475000 | 98525000 | 0.0332 | *TIMM23B* |
| 14 | 124150000 | 124200000 | 0.0341 | *INA* |
| 14 | 124150000 | 124200000 | 0.0341 | *PCGF6* |
| 14 | 124175000 | 124225000 | 0.0364 | *INA* |
| 14 | 124175000 | 124225000 | 0.0364 | *PCGF6* |
| 14 | 127975000 | 128025000 | 0.0335 | *SORCS1* |
| 14 | 143625000 | 143675000 | 0.0285 | *PLEKHG2* |
| 14 | 143625000 | 143675000 | 0.0285 | *RPS16* |
| 14 | 143625000 | 143675000 | 0.0285 | *RPS16P1* |
| 14 | 143625000 | 143675000 | 0.0285 | *RPS16P10* |
| 14 | 143650000 | 143700000 | 0.0312 | *MED29* |
| 14 | 143650000 | 143700000 | 0.0312 | *PAF1* |
| 14 | 143650000 | 143700000 | 0.0312 | *PLEKHG2* |
| 14 | 143650000 | 143700000 | 0.0312 | *RPS16* |
| 14 | 143650000 | 143700000 | 0.0312 | *RPS16P1* |
| 14 | 143650000 | 143700000 | 0.0312 | *RPS16P10* |
| 14 | 143650000 | 143700000 | 0.0312 | *SAMD4B* |
| 14 | 143650000 | 143700000 | 0.0312 | *ZFP3* |
| 14 | 143650000 | 143700000 | 0.0312 | *ZFP36* |
| 14 | 143775000 | 143825000 | 0.0343 | *IL28A* |
| 14 | 143775000 | 143825000 | 0.0343 | *IL29* |
| 14 | 143775000 | 143825000 | 0.0343 | *LRFN1* |
| 15 | 54500000 | 54550000 | 0.0313 | *ADAM32* |
| 15 | 72400000 | 72450000 | 0.0293 | *CCDC148* |
| 15 | 72425000 | 72475000 | 0.0343 | *CCDC148* |
| 15 | 104475000 | 104525000 | 0.0615 | *WDR75* |
| 15 | 104500000 | 104550000 | 0.0624 | *WDR75* |
| 15 | 104525000 | 104575000 | 0.0657 | *WDR75* |
| 15 | 111750000 | 111800000 | 0.0313 | *C2ORF66* |
| 15 | 111750000 | 111800000 | 0.0313 | *CCDC150* |
| 15 | 111750000 | 111800000 | 0.0313 | *GTF3C3* |
| 15 | 111775000 | 111825000 | 0.0423 | *CCDC150* |
| 15 | 111800000 | 111850000 | 0.0322 | *CCDC150* |
| 15 | 111850000 | 111900000 | 0.0331 | *PGAP1* |
| 15 | 115350000 | 115400000 | 0.0298 | *KCTD18* |
| 15 | 115350000 | 115400000 | 0.0298 | *SGOL2* |
| 15 | 118250000 | 118300000 | 0.0370 | *ABI2* |
| 15 | 118250000 | 118300000 | 0.0370 | *RAPH1* |
| 15 | 124750000 | 124800000 | 0.0287 | *ACADL* |
| 16 | 27150000 | 27200000 | 0.0301 | *C6* |
| 16 | 27150000 | 27200000 | 0.0301 | *HEATR7B2* |
| 16 | 28975000 | 29025000 | 0.0303 | *CCDC152* |
| 16 | 28975000 | 29025000 | 0.0303 | *SEPP1* |
| 16 | 29600000 | 29650000 | 0.0287 | *C5ORF34* |
| 16 | 29600000 | 29650000 | 0.0287 | *C6* |
| 16 | 29600000 | 29650000 | 0.0287 | *LOC645139* |
| 16 | 29600000 | 29650000 | 0.0287 | *NNT* |
| 16 | 29600000 | 29650000 | 0.0287 | *PAIP1* |
| 16 | 47875000 | 47925000 | 0.0296 | *ERBB2IP* |
| 16 | 47875000 | 47925000 | 0.0296 | *SFRS12* |
| 16 | 47900000 | 47950000 | 0.0444 | *ERBB2IP* |
| 16 | 47900000 | 47950000 | 0.0444 | *SFRS12* |
| 16 | 47925000 | 47975000 | 0.0351 | *SFRS12* |
| 16 | 51150000 | 51200000 | 0.0321 | *CCNB1* |
| 16 | 51150000 | 51200000 | 0.0321 | *SLC30A5* |
| 16 | 51175000 | 51225000 | 0.0330 | *SLC30A5* |
| 16 | 59075000 | 59125000 | 0.0287 | *SLIT3* |
| 16 | 65075000 | 65125000 | 0.0326 | *MAT2B* |
| 16 | 65100000 | 65150000 | 0.0290 | *HMMR* |
| 16 | 65100000 | 65150000 | 0.0290 | *MAT2B* |
| 16 | 67050000 | 67100000 | 0.0344 | *GABRA6* |
| 16 | 72500000 | 72550000 | 0.0323 | *SGCD* |
| 16 | 72525000 | 72575000 | 0.0298 | *SGCD* |
| 17 | 12875000 | 12925000 | 0.0410 | *SLC20A2* |
| 17 | 36100000 | 36150000 | 0.0332 | *PANK2* |
| 17 | 36100000 | 36150000 | 0.0332 | *RNF24* |
| 17 | 36125000 | 36175000 | 0.0345 | *PANK2* |
| 17 | 36125000 | 36175000 | 0.0345 | *RNF24* |
| 17 | 43625000 | 43675000 | 0.0332 | *TRPC4AP* |
| 17 | 43850000 | 43900000 | 0.0397 | *UQCC* |
| 17 | 44375000 | 44425000 | 0.0297 | *C6* |
| 17 | 44375000 | 44425000 | 0.0297 | *LOC643167* |
| 17 | 44375000 | 44425000 | 0.0297 | *NFS1* |
| 17 | 44375000 | 44425000 | 0.0297 | *RBM39* |
| 17 | 44375000 | 44425000 | 0.0297 | *ROMO1* |
| 17 | 45200000 | 45250000 | 0.0301 | *DLGAP4* |
| 17 | 46400000 | 46450000 | 0.0314 | *CTNNBL1* |
| 17 | 46400000 | 46450000 | 0.0314 | *VSTM2L* |
| 17 | 46425000 | 46475000 | 0.0326 | *KIAA0406* |
| 17 | 46425000 | 46475000 | 0.0326 | *VSTM2L* |
| 17 | 51600000 | 51650000 | 0.0323 | *GK* |
| 17 | 51600000 | 51650000 | 0.0323 | *SGK2* |
| 17 | 53100000 | 53150000 | 0.0307 | *KCNS1* |
| 17 | 53100000 | 53150000 | 0.0307 | *PI3* |
| 17 | 53100000 | 53150000 | 0.0307 | *WFDC12* |
| 17 | 53100000 | 53150000 | 0.0307 | *WFDC5* |
| 17 | 57100000 | 57150000 | 0.0323 | *CSE1L* |
| 17 | 57100000 | 57150000 | 0.0323 | *DDX27* |
| 18 | 1225000 | 1275000 | 0.0298 | *PTPRN2* |
| 18 | 5550000 | 5600000 | 0.0306 | *MLL* |
| 18 | 5550000 | 5600000 | 0.0306 | *MLL3* |
| 18 | 5575000 | 5625000 | 0.0302 | *GALNT11* |
| 18 | 5575000 | 5625000 | 0.0302 | *MLL* |
| 18 | 5575000 | 5625000 | 0.0302 | *MLL3* |
| 18 | 7125000 | 7175000 | 0.0295 | *CLC* |
| 18 | 7125000 | 7175000 | 0.0295 | *CLCN1* |
| 18 | 7125000 | 7175000 | 0.0295 | *FAM131B* |
| 18 | 7150000 | 7200000 | 0.0295 | *CASP2* |
| 18 | 7150000 | 7200000 | 0.0295 | *CLC* |
| 18 | 7150000 | 7200000 | 0.0295 | *CLCN1* |
| X | 14225000 | 14275000 | 0.0315 | *CXORF15* |
| X | 14250000 | 14300000 | 0.0291 | *CXORF15* |
| X | 14250000 | 14300000 | 0.0291 | *RBBP7* |
| X | 28625000 | 28675000 | 0.0332 | *CXORF21* |
| X | 28825000 | 28875000 | 0.0459 | *GK* |
| X | 28825000 | 28875000 | 0.0459 | *GK3P* |
| X | 28850000 | 28900000 | 0.0597 | *GK* |
| X | 28850000 | 28900000 | 0.0597 | *GK3P* |
| X | 28925000 | 28975000 | 0.0406 | *TAB3* |
| X | 28950000 | 29000000 | 0.0476 | *TAB3* |
| X | 28975000 | 29025000 | 0.0505 | *TAB3* |
| X | 51775000 | 51825000 | 0.0330 | *HUWE1* |
| X | 80000000 | 80050000 | 0.0305 | *DACH2* |
| X | 92700000 | 92750000 | 0.0727 | *FAM133A* |
| X | 106750000 | 106800000 | 0.0318 | *LHFPL1* |
| X | 114950000 | 115000000 | 0.0343 | *KLHL13* |
| X | 141000000 | 141050000 | 0.0378 | *GABRA3* |
| X | 141025000 | 141075000 | 0.0297 | *GABRA3* |
| X | 141050000 | 141100000 | 0.0287 | *GABRA3* |
| X | 141275000 | 141325000 | 0.0343 | *GABRQ* |
| X | 141300000 | 141350000 | 0.0341 | *GABRQ* |
| X | 141300000 | 141350000 | 0.0341 | *MAG* |
| X | 141300000 | 141350000 | 0.0341 | *MAGEA6* |
| X | 141325000 | 141375000 | 0.0358 | *CETN2* |
| X | 141325000 | 141375000 | 0.0358 | *MAG* |
| X | 141325000 | 141375000 | 0.0358 | *MAGEA6* |
| X | 141350000 | 141400000 | 0.0444 | *CETN2* |
| X | 141350000 | 141400000 | 0.0444 | *NSDHL* |
| X | 141375000 | 141425000 | 0.0397 | *CETN2* |
| X | 141375000 | 141425000 | 0.0397 | *NSDHL* |
| X | 142250000 | 142300000 | 0.0561 | *HCFC1* |
| X | 142250000 | 142300000 | 0.0561 | *IRAK1* |
| X | 142250000 | 142300000 | 0.0561 | *TMEM187* |
| X | 142275000 | 142325000 | 0.0738 | *IRAK1* |
| X | 142275000 | 142325000 | 0.0738 | *MECP2* |
| X | 142300000 | 142350000 | 0.0753 | *MECP2* |
| X | 143200000 | 143250000 | 0.0317 | *F8* |
| X | 143200000 | 143250000 | 0.0317 | *F8A1* |
| X | 143200000 | 143250000 | 0.0317 | *F8A2* |
| X | 143200000 | 143250000 | 0.0317 | *F8A3* |
| X | 143200000 | 143250000 | 0.0317 | *H2AFB1* |
| X | 143200000 | 143250000 | 0.0317 | *H2AFB2* |
| X | 143200000 | 143250000 | 0.0317 | *H2AFB3* |
| X | 143200000 | 143250000 | 0.0317 | *MPP1* |
| X | 143225000 | 143275000 | 0.0523 | *DKC1* |
| X | 143225000 | 143275000 | 0.0523 | *GAB3* |
| X | 143225000 | 143275000 | 0.0523 | *MPP1* |
| X | 143250000 | 143300000 | 0.0407 | *DKC1* |
| X | 143250000 | 143300000 | 0.0407 | *GAB3* |
| X | 143250000 | 143300000 | 0.0407 | *MPP1* |
| **LL level** | | | | |
| 1 | 35200000 | 35250000 | 0.0106 | *ENPP1* |
| 1 | 61975000 | 62025000 | 0.0101 | *HTR1E* |
| 1 | 82025000 | 82075000 | 0.0121 | *AIM1* |
| 1 | 82025000 | 82075000 | 0.0121 | *RTN4IP1* |
| 1 | 82050000 | 82100000 | 0.0099 | *AIM1* |
| 1 | 82050000 | 82100000 | 0.0099 | *RTN4IP1* |
| 1 | 82900000 | 82950000 | 0.0124 | *SOBP* |
| 1 | 82925000 | 82975000 | 0.0121 | *SOBP* |
| 1 | 85200000 | 85250000 | 0.0102 | *GPR6* |
| 1 | 86450000 | 86500000 | 0.0109 | *CDK19* |
| 1 | 86875000 | 86925000 | 0.0111 | *REV3L* |
| 1 | 86925000 | 86975000 | 0.0133 | *REV3L* |
| 1 | 86950000 | 87000000 | 0.0163 | *REV3L* |
| 1 | 103250000 | 103300000 | 0.0141 | *CD109* |
| 1 | 104475000 | 104525000 | 0.0098 | *SETBP1* |
| 1 | 104500000 | 104550000 | 0.0098 | *SETBP1* |
| 1 | 110150000 | 110200000 | 0.0116 | *MAPK4* |
| 1 | 110175000 | 110225000 | 0.0099 | *MRO* |
| 1 | 112850000 | 112900000 | 0.0115 | *DCC* |
| 1 | 155250000 | 155300000 | 0.0161 | *CERS3* |
| 1 | 155275000 | 155325000 | 0.0122 | *CERS3* |
| 1 | 233675000 | 233725000 | 0.0161 | *TYRP1* |
| 1 | 280900000 | 280950000 | 0.0120 | *PTPN3* |
| 1 | 280925000 | 280975000 | 0.0120 | *PTPN3* |
| 1 | 299075000 | 299125000 | 0.0102 | *NR5A1* |
| 1 | 299075000 | 299125000 | 0.0102 | *NR6A1* |
| 1 | 299100000 | 299150000 | 0.0103 | *NR5A1* |
| 1 | 299100000 | 299150000 | 0.0103 | *NR6A1* |
| 1 | 300950000 | 301000000 | 0.0103 | *FAM125B* |
| 1 | 312375000 | 312425000 | 0.0102 | *OR8H1* |
| 2 | 26675000 | 26725000 | 0.0174 | *RAG2* |
| 2 | 26700000 | 26750000 | 0.0132 | *RAG1* |
| 2 | 26700000 | 26750000 | 0.0132 | *RAG2* |
| 2 | 26725000 | 26775000 | 0.0157 | *RAG1* |
| 2 | 26725000 | 26775000 | 0.0157 | *TRAF6* |
| 2 | 26750000 | 26800000 | 0.0166 | *TRAF6* |
| 2 | 26850000 | 26900000 | 0.0109 | *PRR5L* |
| 2 | 26875000 | 26925000 | 0.0099 | *PRR5L* |
| 2 | 27075000 | 27125000 | 0.0138 | *COMMD9* |
| 2 | 27450000 | 27500000 | 0.0169 | *LDLRAD3* |
| 2 | 27475000 | 27525000 | 0.0111 | *LDLRAD3* |
| 2 | 27675000 | 27725000 | 0.0140 | *TRIM44* |
| 2 | 27700000 | 27750000 | 0.0120 | *TRIM44* |
| 2 | 28025000 | 28075000 | 0.0106 | *SLC1A2* |
| 2 | 28075000 | 28125000 | 0.0117 | *SLC1A2* |
| 2 | 28100000 | 28150000 | 0.0111 | *SLC1A2* |
| 2 | 28125000 | 28175000 | 0.0096 | *CD44* |
| 2 | 28125000 | 28175000 | 0.0096 | *SLC1A2* |
| 2 | 28150000 | 28200000 | 0.0116 | *CD44* |
| 2 | 28625000 | 28675000 | 0.0114 | *EHF* |
| 2 | 28650000 | 28700000 | 0.0106 | *EHF* |
| 2 | 55475000 | 55525000 | 0.0103 | *OR2M4* |
| 2 | 55500000 | 55550000 | 0.0147 | *OR2M4* |
| 2 | 55525000 | 55575000 | 0.0125 | *OR2M2* |
| 2 | 55525000 | 55575000 | 0.0125 | *OR2M3* |
| 2 | 55525000 | 55575000 | 0.0125 | *OR2M7* |
| 2 | 56475000 | 56525000 | 0.0103 | *TRIM58* |
| 2 | 57525000 | 57575000 | 0.0125 | *OR2G2* |
| 2 | 57525000 | 57575000 | 0.0125 | *OR2G3* |
| 2 | 57600000 | 57650000 | 0.0099 | *C1ORF150* |
| 2 | 67725000 | 67775000 | 0.0177 | *OR7D2* |
| 3 | 625000 | 675000 | 0.0165 | *ADAP1* |
| 3 | 625000 | 675000 | 0.0165 | *DAP* |
| 3 | 625000 | 675000 | 0.0165 | *GET4* |
| 3 | 625000 | 675000 | 0.0165 | *SUN1* |
| 3 | 38350000 | 38400000 | 0.0130 | *C16ORF89* |
| 3 | 38350000 | 38400000 | 0.0130 | *NAGPA* |
| 3 | 38375000 | 38425000 | 0.0127 | *NAGPA* |
| 3 | 38375000 | 38425000 | 0.0127 | *SEC14L5* |
| 3 | 38400000 | 38450000 | 0.0122 | *SEC14L5* |
| 3 | 38450000 | 38500000 | 0.0123 | *PPL* |
| 3 | 38475000 | 38525000 | 0.0157 | *PPL* |
| 3 | 38475000 | 38525000 | 0.0157 | *UBN1* |
| 3 | 38500000 | 38550000 | 0.0173 | *PPL* |
| 3 | 38500000 | 38550000 | 0.0173 | *UBN1* |
| 3 | 38625000 | 38675000 | 0.0148 | *GLYR1* |
| 3 | 38625000 | 38675000 | 0.0148 | *ROGDI* |
| 3 | 38650000 | 38700000 | 0.0175 | *GLYR1* |
| 3 | 38650000 | 38700000 | 0.0175 | *ROGDI* |
| 3 | 38775000 | 38825000 | 0.0096 | *FAM100A* |
| 3 | 38775000 | 38825000 | 0.0096 | *LOC342346* |
| 3 | 38775000 | 38825000 | 0.0096 | *MGRN1* |
| 3 | 38800000 | 38850000 | 0.0114 | *FAM100A* |
| 3 | 38800000 | 38850000 | 0.0114 | *LOC342346* |
| 3 | 38800000 | 38850000 | 0.0114 | *MGRN1* |
| 3 | 38825000 | 38875000 | 0.0140 | *LOC342346* |
| 3 | 38850000 | 38900000 | 0.0137 | *C16ORF5* |
| 3 | 38850000 | 38900000 | 0.0137 | *HMOX2* |
| 3 | 38850000 | 38900000 | 0.0137 | *LOC342346* |
| 3 | 38875000 | 38925000 | 0.0160 | *C16ORF5* |
| 3 | 38875000 | 38925000 | 0.0160 | *HMOX2* |
| 3 | 38900000 | 38950000 | 0.0189 | *DNAJA3* |
| 3 | 38900000 | 38950000 | 0.0189 | *NMRAL1* |
| 3 | 38925000 | 38975000 | 0.0159 | *DNAJA3* |
| 3 | 38925000 | 38975000 | 0.0159 | *NMRAL1* |
| 3 | 38950000 | 39000000 | 0.0203 | *CORO7* |
| 3 | 38950000 | 39000000 | 0.0203 | *DNAJA3* |
| 3 | 38975000 | 39025000 | 0.0191 | *CORO7* |
| 3 | 38975000 | 39025000 | 0.0191 | *VASN* |
| 3 | 39000000 | 39050000 | 0.0176 | *CORO7* |
| 3 | 39000000 | 39050000 | 0.0176 | *TIMM16* |
| 3 | 39000000 | 39050000 | 0.0176 | *VASN* |
| 3 | 39025000 | 39075000 | 0.0192 | *CORO7* |
| 3 | 39025000 | 39075000 | 0.0192 | *GLIS2* |
| 3 | 39025000 | 39075000 | 0.0192 | *TIMM16* |
| 3 | 39050000 | 39100000 | 0.0257 | *GLIS2* |
| 3 | 39050000 | 39100000 | 0.0257 | *TIMM16* |
| 3 | 39100000 | 39150000 | 0.0127 | *ADCY9* |
| 3 | 39125000 | 39175000 | 0.0110 | *ADCY9* |
| 3 | 39200000 | 39250000 | 0.0109 | *ADCY9* |
| 3 | 39200000 | 39250000 | 0.0109 | *SRL* |
| 3 | 39225000 | 39275000 | 0.0201 | *ADCY9* |
| 3 | 39225000 | 39275000 | 0.0201 | *SRL* |
| 3 | 39250000 | 39300000 | 0.0198 | *SRL* |
| 3 | 39400000 | 39450000 | 0.0135 | *ADCY9* |
| 3 | 39500000 | 39550000 | 0.0131 | *CREBBP* |
| 3 | 39525000 | 39575000 | 0.0144 | *CREBBP* |
| 3 | 39550000 | 39600000 | 0.0125 | *CREBBP* |
| 3 | 39575000 | 39625000 | 0.0134 | *CREBBP* |
| 3 | 39600000 | 39650000 | 0.0169 | *CREBBP* |
| 3 | 39725000 | 39775000 | 0.0176 | *CREBBP* |
| 3 | 39775000 | 39825000 | 0.0169 | *CREBBP* |
| 3 | 39775000 | 39825000 | 0.0169 | *TRAP1* |
| 3 | 39800000 | 39850000 | 0.0176 | *TRAP1* |
| 3 | 39825000 | 39875000 | 0.0164 | *DNASE1* |
| 3 | 39825000 | 39875000 | 0.0164 | *TRAP1* |
| 3 | 39850000 | 39900000 | 0.0136 | *DNASE1* |
| 3 | 39850000 | 39900000 | 0.0136 | *TRAP1* |
| 3 | 39875000 | 39925000 | 0.0108 | *BTBD12* |
| 3 | 39950000 | 40000000 | 0.0146 | *BTBD12* |
| 3 | 39975000 | 40025000 | 0.0106 | *BTBD12* |
| 3 | 39975000 | 40025000 | 0.0106 | *CLUAP1* |
| 3 | 40025000 | 40075000 | 0.0155 | *CLUAP1* |
| 3 | 40025000 | 40075000 | 0.0155 | *NLRC3* |
| 3 | 40100000 | 40150000 | 0.0203 | *C16ORF90* |
| 3 | 40100000 | 40150000 | 0.0203 | *OR2C1* |
| 3 | 40100000 | 40150000 | 0.0203 | *ZNF434* |
| 3 | 40150000 | 40200000 | 0.0160 | *NAT15* |
| 3 | 40150000 | 40200000 | 0.0160 | *ZNF174* |
| 3 | 40150000 | 40200000 | 0.0160 | *ZNF597* |
| 3 | 40850000 | 40900000 | 0.0118 | *MPG* |
| 3 | 40850000 | 40900000 | 0.0118 | *NPRL3* |
| 3 | 40850000 | 40900000 | 0.0118 | *POLR3K* |
| 3 | 40850000 | 40900000 | 0.0118 | *RHBDF1* |
| 3 | 40850000 | 40900000 | 0.0118 | *SNRNP25* |
| 3 | 40875000 | 40925000 | 0.0108 | *MPG* |
| 3 | 40875000 | 40925000 | 0.0108 | *NPRL3* |
| 3 | 40875000 | 40925000 | 0.0108 | *RHBDF1* |
| 3 | 41325000 | 41375000 | 0.0115 | *FAM195A* |
| 3 | 41325000 | 41375000 | 0.0115 | *RHOT2* |
| 3 | 41325000 | 41375000 | 0.0115 | *WDR90* |
| 3 | 41350000 | 41400000 | 0.0097 | *FAM195A* |
| 3 | 41350000 | 41400000 | 0.0097 | *FBXL16* |
| 3 | 41350000 | 41400000 | 0.0097 | *JMJD8* |
| 3 | 41350000 | 41400000 | 0.0097 | *RHOT2* |
| 3 | 41350000 | 41400000 | 0.0097 | *STUB1* |
| 3 | 41350000 | 41400000 | 0.0097 | *WDR24* |
| 3 | 41350000 | 41400000 | 0.0097 | *WDR90* |
| 3 | 44200000 | 44250000 | 0.0125 | *FAM120A* |
| 3 | 44225000 | 44275000 | 0.0114 | *C9ORF129* |
| 3 | 44225000 | 44275000 | 0.0114 | *FAM120A* |
| 3 | 44250000 | 44300000 | 0.0105 | *C9ORF129* |
| 3 | 44250000 | 44300000 | 0.0105 | *FAM120A* |
| 3 | 44375000 | 44425000 | 0.0110 | *PHF2* |
| 3 | 44400000 | 44450000 | 0.0110 | *PHF2* |
| 3 | 44425000 | 44475000 | 0.0129 | *PHF2* |
| 3 | 44575000 | 44625000 | 0.0096 | *AR* |
| 3 | 44575000 | 44625000 | 0.0096 | *BARX1* |
| 3 | 45525000 | 45575000 | 0.0114 | *POLR1B* |
| 3 | 45525000 | 45575000 | 0.0114 | *TTL* |
| 3 | 45550000 | 45600000 | 0.0101 | *POLR1B* |
| 3 | 45550000 | 45600000 | 0.0101 | *TTL* |
| 3 | 45925000 | 45975000 | 0.0102 | *MERTK* |
| 3 | 57750000 | 57800000 | 0.0119 | *TSGA10* |
| 3 | 57775000 | 57825000 | 0.0134 | *TSGA10* |
| 3 | 57800000 | 57850000 | 0.0127 | *TSGA10* |
| 3 | 76425000 | 76475000 | 0.0101 | *GFPT1* |
| 3 | 76425000 | 76475000 | 0.0101 | *NFU1* |
| 3 | 84875000 | 84925000 | 0.0122 | *PEX13* |
| 3 | 84875000 | 84925000 | 0.0122 | *PUS10* |
| 3 | 92175000 | 92225000 | 0.0198 | *PSME4* |
| 3 | 92400000 | 92450000 | 0.0110 | *PSME4* |
| 3 | 92425000 | 92475000 | 0.0103 | *PSME4* |
| 3 | 100100000 | 100150000 | 0.0197 | *ATP6V1E2* |
| 3 | 100100000 | 100150000 | 0.0197 | *LOC388946* |
| 3 | 100125000 | 100175000 | 0.0203 | *EPAS1* |
| 3 | 100125000 | 100175000 | 0.0203 | *LOC388946* |
| 3 | 100175000 | 100225000 | 0.0111 | *EPAS1* |
| 3 | 102400000 | 102450000 | 0.0099 | *PPM1B* |
| 3 | 103750000 | 103800000 | 0.0136 | *MTA3* |
| 3 | 104150000 | 104200000 | 0.0112 | *EML4* |
| 3 | 113950000 | 114000000 | 0.0138 | *BIRC6* |
| 3 | 113975000 | 114025000 | 0.0138 | *BIRC6* |
| 3 | 114000000 | 114050000 | 0.0113 | *BIRC6* |
| 3 | 115200000 | 115250000 | 0.0123 | *CAPN13* |
| 3 | 115400000 | 115450000 | 0.0153 | *LCLAT1* |
| 3 | 115425000 | 115475000 | 0.0177 | *LCLAT1* |
| 3 | 115450000 | 115500000 | 0.0133 | *LCLAT1* |
| 4 | 49775000 | 49825000 | 0.0113 | *NECAB1* |
| 4 | 49825000 | 49875000 | 0.0126 | *NECAB1* |
| 4 | 49850000 | 49900000 | 0.0152 | *NECAB1* |
| 4 | 54675000 | 54725000 | 0.0097 | *CNBD1* |
| 4 | 90350000 | 90400000 | 0.0105 | *SFT2D2* |
| 4 | 90350000 | 90400000 | 0.0105 | *TBX19* |
| 4 | 97400000 | 97450000 | 0.0134 | *ITLN1* |
| 4 | 121550000 | 121600000 | 0.0107 | *CLC* |
| 4 | 121550000 | 121600000 | 0.0107 | *CLCC1* |
| 4 | 121550000 | 121600000 | 0.0107 | *GPSM2* |
| 4 | 121550000 | 121600000 | 0.0107 | *WDR47* |
| 4 | 139625000 | 139675000 | 0.0115 | *GBP1* |
| 4 | 139625000 | 139675000 | 0.0115 | *GBP2* |
| 4 | 139625000 | 139675000 | 0.0115 | *GBP3* |
| 4 | 139650000 | 139700000 | 0.0109 | *CCBL2* |
| 4 | 139650000 | 139700000 | 0.0109 | *GBP1* |
| 4 | 139650000 | 139700000 | 0.0109 | *GBP3* |
| 4 | 139750000 | 139800000 | 0.0116 | *GTF2B* |
| 4 | 139775000 | 139825000 | 0.0111 | *GTF2B* |
| 4 | 139775000 | 139825000 | 0.0111 | *PKN2* |
| 4 | 139825000 | 139875000 | 0.0097 | *PKN2* |
| 5 | 1200000 | 1250000 | 0.0136 | *RIBC2* |
| 5 | 1200000 | 1250000 | 0.0136 | *SMC1B* |
| 5 | 1400000 | 1450000 | 0.0098 | *SMC1B* |
| 5 | 50875000 | 50925000 | 0.0123 | *ITPR2* |
| 5 | 51900000 | 51950000 | 0.0104 | *IFLTD1* |
| 5 | 59975000 | 60025000 | 0.0107 | *DERA* |
| 5 | 59975000 | 60025000 | 0.0107 | *STRAP* |
| 5 | 61500000 | 61550000 | 0.0099 | *ATF7IP* |
| 6 | 38800000 | 38850000 | 0.0098 | *PEPD* |
| 6 | 38850000 | 38900000 | 0.0114 | *PEPD* |
| 6 | 40325000 | 40375000 | 0.0120 | *AR* |
| 6 | 40325000 | 40375000 | 0.0120 | *CD22* |
| 6 | 40325000 | 40375000 | 0.0120 | *FFAR1* |
| 6 | 40325000 | 40375000 | 0.0120 | *FFAR3* |
| 6 | 40325000 | 40375000 | 0.0120 | *GAPDHS* |
| 6 | 40325000 | 40375000 | 0.0120 | *MAG* |
| 6 | 43175000 | 43225000 | 0.0099 | *ECH1* |
| 6 | 43175000 | 43225000 | 0.0099 | *HNRNPL* |
| 6 | 43175000 | 43225000 | 0.0099 | *LOC644390* |
| 6 | 43175000 | 43225000 | 0.0099 | *RINL* |
| 6 | 43175000 | 43225000 | 0.0099 | *SI* |
| 6 | 43175000 | 43225000 | 0.0099 | *SIRT2* |
| 6 | 43775000 | 43825000 | 0.0118 | *DLL3* |
| 6 | 43800000 | 43850000 | 0.0122 | *DLL3* |
| 6 | 43800000 | 43850000 | 0.0122 | *SUPT5H* |
| 6 | 43800000 | 43850000 | 0.0122 | *TIMM50* |
| 6 | 43825000 | 43875000 | 0.0122 | *SUPT5H* |
| 6 | 43825000 | 43875000 | 0.0122 | *TIMM50* |
| 6 | 43850000 | 43900000 | 0.0157 | *SUPT5H* |
| 6 | 43900000 | 43950000 | 0.0136 | *CLC* |
| 6 | 43900000 | 43950000 | 0.0136 | *LGALS13* |
| 6 | 43900000 | 43950000 | 0.0136 | *LGALS14* |
| 6 | 43925000 | 43975000 | 0.0125 | *CLC* |
| 6 | 43925000 | 43975000 | 0.0125 | *EID2* |
| 6 | 43925000 | 43975000 | 0.0125 | *EID2B* |
| 6 | 43925000 | 43975000 | 0.0125 | *LGALS13* |
| 6 | 43925000 | 43975000 | 0.0125 | *LGALS14* |
| 6 | 43950000 | 44000000 | 0.0105 | *EID2* |
| 6 | 43950000 | 44000000 | 0.0105 | *EID2B* |
| 6 | 43950000 | 44000000 | 0.0105 | *SELV* |
| 6 | 43975000 | 44025000 | 0.0101 | *LEUTX* |
| 6 | 43975000 | 44025000 | 0.0101 | *SELV* |
| 6 | 44000000 | 44050000 | 0.0105 | *LEUTX* |
| 6 | 44075000 | 44125000 | 0.0114 | *CGB* |
| 6 | 44075000 | 44125000 | 0.0114 | *FCGBP* |
| 6 | 44100000 | 44150000 | 0.0120 | *CGB* |
| 6 | 44100000 | 44150000 | 0.0120 | *FCGBP* |
| 6 | 44550000 | 44600000 | 0.0101 | *LTBP4* |
| 6 | 44575000 | 44625000 | 0.0155 | *ADCK4* |
| 6 | 44575000 | 44625000 | 0.0155 | *LTBP4* |
| 6 | 44600000 | 44650000 | 0.0158 | *ADCK4* |
| 6 | 44600000 | 44650000 | 0.0158 | *C19ORF54* |
| 6 | 44600000 | 44650000 | 0.0158 | *ITPKC* |
| 6 | 44600000 | 44650000 | 0.0158 | *LTBP4* |
| 6 | 44625000 | 44675000 | 0.0139 | *C19ORF54* |
| 6 | 44625000 | 44675000 | 0.0139 | *ITPKC* |
| 6 | 44625000 | 44675000 | 0.0139 | *MIA* |
| 6 | 44625000 | 44675000 | 0.0139 | *RAB4B* |
| 6 | 44625000 | 44675000 | 0.0139 | *SNRPA* |
| 6 | 44650000 | 44700000 | 0.0140 | *C19ORF54* |
| 6 | 44650000 | 44700000 | 0.0140 | *EGLN2* |
| 6 | 44650000 | 44700000 | 0.0140 | *MIA* |
| 6 | 44650000 | 44700000 | 0.0140 | *RAB4B* |
| 6 | 44650000 | 44700000 | 0.0140 | *SNRPA* |
| 6 | 44675000 | 44725000 | 0.0139 | *ADCK4* |
| 6 | 44675000 | 44725000 | 0.0139 | *CYP2F1* |
| 6 | 44675000 | 44725000 | 0.0139 | *EGLN2* |
| 6 | 44675000 | 44725000 | 0.0139 | *NUMBL* |
| 6 | 44675000 | 44725000 | 0.0139 | *RAB4B* |
| 6 | 44700000 | 44750000 | 0.0153 | *ADCK4* |
| 6 | 44700000 | 44750000 | 0.0153 | *CYP2F1* |
| 6 | 44700000 | 44750000 | 0.0153 | *NUMBL* |
| 6 | 44725000 | 44775000 | 0.0170 | *NUMBL* |
| 6 | 44750000 | 44800000 | 0.0160 | *LTBP4* |
| 6 | 44750000 | 44800000 | 0.0160 | *SHKBP1* |
| 6 | 44875000 | 44925000 | 0.0121 | *CYP2A13* |
| 6 | 44875000 | 44925000 | 0.0121 | *CYP2A6* |
| 6 | 44875000 | 44925000 | 0.0121 | *CYP2A7* |
| 6 | 44875000 | 44925000 | 0.0121 | *CYP2F1* |
| 6 | 44900000 | 44950000 | 0.0146 | *CYP2B6* |
| 6 | 44900000 | 44950000 | 0.0146 | *CYP2F1* |
| 6 | 44900000 | 44950000 | 0.0146 | *CYP2S1* |
| 6 | 45050000 | 45100000 | 0.0167 | *HNRNPUL1* |
| 6 | 45050000 | 45100000 | 0.0167 | *TGFB1* |
| 6 | 45075000 | 45125000 | 0.0173 | *CCDC97* |
| 6 | 45075000 | 45125000 | 0.0173 | *HNRNPUL1* |
| 6 | 45475000 | 45525000 | 0.0102 | *MEGF8* |
| 6 | 45475000 | 45525000 | 0.0102 | *TMEM145* |
| 6 | 45500000 | 45550000 | 0.0158 | *CIC* |
| 6 | 45500000 | 45550000 | 0.0158 | *MEGF8* |
| 6 | 45500000 | 45550000 | 0.0158 | *PAFAH1B3* |
| 6 | 45500000 | 45550000 | 0.0158 | *PRR19* |
| 6 | 45500000 | 45550000 | 0.0158 | *TMEM145* |
| 6 | 45525000 | 45575000 | 0.0207 | *CIC* |
| 6 | 45525000 | 45575000 | 0.0207 | *PAFAH1B3* |
| 6 | 45525000 | 45575000 | 0.0207 | *PRR19* |
| 6 | 45550000 | 45600000 | 0.0139 | *ERF* |
| 6 | 45575000 | 45625000 | 0.0179 | *ERF* |
| 6 | 45575000 | 45625000 | 0.0179 | *GSK3A* |
| 6 | 45575000 | 45625000 | 0.0179 | *ZNF526* |
| 6 | 45600000 | 45650000 | 0.0203 | *DEDD2* |
| 6 | 45600000 | 45650000 | 0.0203 | *ERF* |
| 6 | 45600000 | 45650000 | 0.0203 | *GSK3A* |
| 6 | 45600000 | 45650000 | 0.0203 | *ZNF526* |
| 6 | 45625000 | 45675000 | 0.0170 | *DEDD2* |
| 6 | 45750000 | 45800000 | 0.0097 | *AR* |
| 6 | 45750000 | 45800000 | 0.0097 | *ARHGEF1* |
| 6 | 45750000 | 45800000 | 0.0097 | *GRIK5* |
| 6 | 45850000 | 45900000 | 0.0101 | *GRIK5* |
| 6 | 45850000 | 45900000 | 0.0101 | *POU2F2* |
| 6 | 45900000 | 45950000 | 0.0106 | *ATP1A3* |
| 6 | 45900000 | 45950000 | 0.0106 | *GRIK5* |
| 6 | 45975000 | 46025000 | 0.0110 | *CXCL17* |
| 6 | 45975000 | 46025000 | 0.0110 | *LIPE* |
| 6 | 46000000 | 46050000 | 0.0099 | *CD79A* |
| 6 | 46125000 | 46175000 | 0.0143 | *ETHE1* |
| 6 | 46125000 | 46175000 | 0.0143 | *TEX101* |
| 6 | 46125000 | 46175000 | 0.0143 | *XRCC1* |
| 6 | 46125000 | 46175000 | 0.0143 | *ZNF575* |
| 6 | 46150000 | 46200000 | 0.0176 | *IRGQ* |
| 6 | 46150000 | 46200000 | 0.0176 | *XRCC1* |
| 6 | 46150000 | 46200000 | 0.0176 | *ZNF575* |
| 6 | 46150000 | 46200000 | 0.0176 | *ZNF576* |
| 6 | 46175000 | 46225000 | 0.0156 | *CADM4* |
| 6 | 46175000 | 46225000 | 0.0156 | *IRGQ* |
| 6 | 46175000 | 46225000 | 0.0156 | *SRRM5* |
| 6 | 46175000 | 46225000 | 0.0156 | *XRCC1* |
| 6 | 46175000 | 46225000 | 0.0156 | *ZNF428* |
| 6 | 46175000 | 46225000 | 0.0156 | *ZNF576* |
| 6 | 46200000 | 46250000 | 0.0102 | *CADM4* |
| 6 | 46200000 | 46250000 | 0.0102 | *ETHE1* |
| 6 | 46200000 | 46250000 | 0.0102 | *PHLDB3* |
| 6 | 46200000 | 46250000 | 0.0102 | *SRRM5* |
| 6 | 46200000 | 46250000 | 0.0102 | *ZNF428* |
| 6 | 46225000 | 46275000 | 0.0127 | *ETHE1* |
| 6 | 46225000 | 46275000 | 0.0127 | *LYPD3* |
| 6 | 46225000 | 46275000 | 0.0127 | *PHLDB3* |
| 6 | 46250000 | 46300000 | 0.0181 | *LYPD3* |
| 6 | 46250000 | 46300000 | 0.0181 | *PHLDB3* |
| 6 | 46325000 | 46375000 | 0.0151 | *PLAUR* |
| 6 | 46350000 | 46400000 | 0.0138 | *PLAUR* |
| 6 | 46625000 | 46675000 | 0.0100 | *ZNF155* |
| 6 | 46625000 | 46675000 | 0.0100 | *ZNF221* |
| 6 | 46625000 | 46675000 | 0.0100 | *ZNF223* |
| 6 | 46625000 | 46675000 | 0.0100 | *ZNF224* |
| 6 | 46625000 | 46675000 | 0.0100 | *ZNF225* |
| 6 | 46625000 | 46675000 | 0.0100 | *ZNF226* |
| 6 | 46625000 | 46675000 | 0.0100 | *ZNF230* |
| 6 | 46625000 | 46675000 | 0.0100 | *ZNF234* |
| 6 | 46625000 | 46675000 | 0.0100 | *ZNF284* |
| 6 | 46625000 | 46675000 | 0.0100 | *ZNF285* |
| 6 | 46625000 | 46675000 | 0.0100 | *ZNF285B* |
| 6 | 46625000 | 46675000 | 0.0100 | *ZNF45* |
| 6 | 46625000 | 46675000 | 0.0100 | *ZNF806* |
| 6 | 47200000 | 47250000 | 0.0105 | *BCAM* |
| 6 | 47200000 | 47250000 | 0.0105 | *PVRL2* |
| 6 | 47275000 | 47325000 | 0.0120 | *APOC1* |
| 6 | 47275000 | 47325000 | 0.0120 | *APOC2* |
| 6 | 47275000 | 47325000 | 0.0120 | *APOC4* |
| 6 | 47275000 | 47325000 | 0.0120 | *CLPTM1* |
| 6 | 47275000 | 47325000 | 0.0120 | *TOMM40* |
| 6 | 47300000 | 47350000 | 0.0123 | *CLPTM1* |
| 6 | 47300000 | 47350000 | 0.0123 | *PVRL2* |
| 6 | 47300000 | 47350000 | 0.0123 | *RELB* |
| 6 | 47300000 | 47350000 | 0.0123 | *TOMM40* |
| 6 | 49075000 | 49125000 | 0.0116 | *CRX* |
| 6 | 49075000 | 49125000 | 0.0116 | *SULT2A1* |
| 6 | 49075000 | 49125000 | 0.0116 | *TPRX1* |
| 6 | 50050000 | 50100000 | 0.0117 | *CGB* |
| 6 | 50050000 | 50100000 | 0.0117 | *CGB1* |
| 6 | 50050000 | 50100000 | 0.0117 | *CGB2* |
| 6 | 50050000 | 50100000 | 0.0117 | *CGB5* |
| 6 | 50050000 | 50100000 | 0.0117 | *CGB7* |
| 6 | 50050000 | 50100000 | 0.0117 | *CGB8* |
| 6 | 50050000 | 50100000 | 0.0117 | *FTL* |
| 6 | 50050000 | 50100000 | 0.0117 | *FTLP2* |
| 6 | 50050000 | 50100000 | 0.0117 | *GYS1* |
| 6 | 50050000 | 50100000 | 0.0117 | *LHB* |
| 6 | 50050000 | 50100000 | 0.0117 | *NTF4* |
| 6 | 50050000 | 50100000 | 0.0117 | *RUVBL2* |
| 6 | 145325000 | 145375000 | 0.0120 | *USP24* |
| 6 | 145350000 | 145400000 | 0.0173 | *USP24* |
| 6 | 145375000 | 145425000 | 0.0156 | *USP24* |
| 6 | 145400000 | 145450000 | 0.0102 | *USP24* |
| 7 | 20625000 | 20675000 | 0.0121 | *ACOT13* |
| 7 | 20625000 | 20675000 | 0.0121 | *C6ORF62* |
| 7 | 20625000 | 20675000 | 0.0121 | *TTRAP* |
| 7 | 20650000 | 20700000 | 0.0152 | *ACOT13* |
| 7 | 20650000 | 20700000 | 0.0152 | *C6ORF62* |
| 7 | 20650000 | 20700000 | 0.0152 | *TTRAP* |
| 7 | 61725000 | 61775000 | 0.0104 | *SCAPER* |
| 7 | 128400000 | 128450000 | 0.0104 | *EML1* |
| 7 | 128625000 | 128675000 | 0.0103 | *C14ORF68* |
| 7 | 128625000 | 128675000 | 0.0103 | *SLC25A29* |
| 7 | 128625000 | 128675000 | 0.0103 | *YY1* |
| 7 | 128650000 | 128700000 | 0.0098 | *AR* |
| 7 | 128650000 | 128700000 | 0.0098 | *C14ORF68* |
| 7 | 128650000 | 128700000 | 0.0098 | *SLC25A29* |
| 7 | 128650000 | 128700000 | 0.0098 | *WARS* |
| 8 | 48225000 | 48275000 | 0.0100 | *GRIA2* |
| 8 | 53125000 | 53175000 | 0.0116 | *FSTL5* |
| 8 | 55075000 | 55125000 | 0.0115 | *NAF1* |
| 8 | 55100000 | 55150000 | 0.0156 | *NAF1* |
| 8 | 55125000 | 55175000 | 0.0170 | *NAF1* |
| 8 | 57725000 | 57775000 | 0.0169 | *AR* |
| 8 | 57725000 | 57775000 | 0.0169 | *ARL9* |
| 8 | 57750000 | 57800000 | 0.0164 | *AR* |
| 8 | 57750000 | 57800000 | 0.0164 | *ARL9* |
| 8 | 57750000 | 57800000 | 0.0164 | *SRP72* |
| 8 | 57775000 | 57825000 | 0.0161 | *AR* |
| 8 | 57775000 | 57825000 | 0.0161 | *ARL9* |
| 8 | 57775000 | 57825000 | 0.0161 | *SRP72* |
| 8 | 57800000 | 57850000 | 0.0152 | *PAICS* |
| 8 | 57800000 | 57850000 | 0.0152 | *PPAT* |
| 8 | 57800000 | 57850000 | 0.0152 | *SRP72* |
| 8 | 57825000 | 57875000 | 0.0161 | *PAICS* |
| 8 | 57825000 | 57875000 | 0.0161 | *PPAT* |
| 8 | 57850000 | 57900000 | 0.0165 | *PPAT* |
| 8 | 57875000 | 57925000 | 0.0156 | *AASDH* |
| 8 | 57875000 | 57925000 | 0.0156 | *KIAA1211* |
| 8 | 57875000 | 57925000 | 0.0156 | *PPAT* |
| 8 | 57900000 | 57950000 | 0.0149 | *AASDH* |
| 8 | 57900000 | 57950000 | 0.0149 | *KIAA1211* |
| 8 | 57925000 | 57975000 | 0.0149 | *KIAA1211* |
| 8 | 57950000 | 58000000 | 0.0151 | *AASDH* |
| 8 | 57975000 | 58025000 | 0.0154 | *AASDH* |
| 8 | 57975000 | 58025000 | 0.0154 | *KIAA1211* |
| 8 | 58000000 | 58050000 | 0.0160 | *KIAA1211* |
| 8 | 67425000 | 67475000 | 0.0103 | *EPHA5* |
| 8 | 67450000 | 67500000 | 0.0096 | *EPHA5* |
| 8 | 69675000 | 69725000 | 0.0098 | *CENPC1* |
| 8 | 69700000 | 69750000 | 0.0097 | *CENPC1* |
| 8 | 69850000 | 69900000 | 0.0104 | *UBA6* |
| 8 | 69875000 | 69925000 | 0.0144 | *GNRHR* |
| 8 | 69875000 | 69925000 | 0.0144 | *UBA6* |
| 8 | 69900000 | 69950000 | 0.0133 | *GNRHR* |
| 8 | 69950000 | 70000000 | 0.0136 | *TMPRSS11D* |
| 8 | 79925000 | 79975000 | 0.0105 | *KIAA0922* |
| 8 | 82950000 | 83000000 | 0.0105 | *LRBA* |
| 8 | 82975000 | 83025000 | 0.0122 | *LRBA* |
| 8 | 83025000 | 83075000 | 0.0103 | *LRBA* |
| 8 | 83050000 | 83100000 | 0.0096 | *LRBA* |
| 8 | 85900000 | 85950000 | 0.0099 | *AR* |
| 8 | 85900000 | 85950000 | 0.0099 | *ARHGAP10* |
| 8 | 148275000 | 148325000 | 0.0108 | *HTN3* |
| 8 | 148300000 | 148350000 | 0.0122 | *C4ORF40* |
| 8 | 148325000 | 148375000 | 0.0098 | *C4ORF40* |
| 8 | 148400000 | 148450000 | 0.0112 | *CSN3* |
| 8 | 148425000 | 148475000 | 0.0098 | *CSN3* |
| 8 | 148425000 | 148475000 | 0.0098 | *ODAM* |
| 9 | 58025000 | 58075000 | 0.0110 | *FEZ1* |
| 9 | 80125000 | 80175000 | 0.0098 | *CALCR* |
| 9 | 88700000 | 88750000 | 0.0098 | *PHF14* |
| 9 | 88875000 | 88925000 | 0.0099 | *THSD7A* |
| 9 | 93400000 | 93450000 | 0.0155 | *TMEM195* |
| 9 | 138950000 | 139000000 | 0.0095 | *IVNS1ABP* |
| 10 | 14375000 | 14425000 | 0.0102 | *RGS7* |
| 10 | 15025000 | 15075000 | 0.0133 | *DEGS1* |
| 10 | 15025000 | 15075000 | 0.0133 | *NVL* |
| 10 | 15075000 | 15125000 | 0.0169 | *CNIH4* |
| 10 | 15375000 | 15425000 | 0.0097 | *CNIH3* |
| 10 | 15475000 | 15525000 | 0.0100 | *DNAH14* |
| 10 | 16950000 | 17000000 | 0.0110 | *CDC42BPA* |
| 10 | 16975000 | 17025000 | 0.0127 | *CDC42BPA* |
| 10 | 17000000 | 17050000 | 0.0184 | *CDC42BPA* |
| 10 | 17025000 | 17075000 | 0.0127 | *CDC42BPA* |
| 10 | 17425000 | 17475000 | 0.0106 | *AHCTF1* |
| 10 | 17425000 | 17475000 | 0.0106 | *AHCTF1P1* |
| 10 | 17475000 | 17525000 | 0.0164 | *SCCPDH* |
| 10 | 17500000 | 17550000 | 0.0135 | *SCCPDH* |
| 10 | 17525000 | 17575000 | 0.0100 | *SCCPDH* |
| 10 | 17550000 | 17600000 | 0.0097 | *SCCPDH* |
| 10 | 17600000 | 17650000 | 0.0110 | *EXO1* |
| 10 | 17625000 | 17675000 | 0.0110 | *EXO1* |
| 10 | 17650000 | 17700000 | 0.0127 | *EXO1* |
| 10 | 17650000 | 17700000 | 0.0127 | *MAP1LC3C* |
| 10 | 17675000 | 17725000 | 0.0101 | *MAP1LC3C* |
| 10 | 17725000 | 17775000 | 0.0132 | *PLD5* |
| 10 | 18275000 | 18325000 | 0.0101 | *DCC* |
| 10 | 18275000 | 18325000 | 0.0101 | *SDCCAG8* |
| 10 | 29250000 | 29300000 | 0.0102 | *ADIPOR1* |
| 10 | 29250000 | 29300000 | 0.0102 | *CYB5R1* |
| 10 | 29250000 | 29300000 | 0.0102 | *KDM5B* |
| 10 | 29250000 | 29300000 | 0.0102 | *KLHL12* |
| 10 | 29250000 | 29300000 | 0.0102 | *LOC100133760* |
| 10 | 30875000 | 30925000 | 0.0116 | *PTCH1* |
| 10 | 63650000 | 63700000 | 0.0096 | *CCNY* |
| 10 | 63650000 | 63700000 | 0.0096 | *FZD8* |
| 10 | 63650000 | 63700000 | 0.0096 | *GJD4* |
| 11 | 76700000 | 76750000 | 0.0102 | *TMTC4* |
| 12 | 2100000 | 2150000 | 0.0170 | *RNF213* |
| 12 | 2150000 | 2200000 | 0.0265 | *AR* |
| 12 | 2150000 | 2200000 | 0.0265 | *CARD14* |
| 12 | 2150000 | 2200000 | 0.0265 | *RNF213* |
| 12 | 2150000 | 2200000 | 0.0265 | *SGSH* |
| 12 | 2150000 | 2200000 | 0.0265 | *SLC26A11* |
| 12 | 2175000 | 2225000 | 0.0252 | *AR* |
| 12 | 2175000 | 2225000 | 0.0252 | *CARD14* |
| 12 | 2175000 | 2225000 | 0.0252 | *SGSH* |
| 12 | 2175000 | 2225000 | 0.0252 | *SLC26A11* |
| 12 | 2200000 | 2250000 | 0.0271 | *AR* |
| 12 | 2200000 | 2250000 | 0.0271 | *CARD14* |
| 12 | 2225000 | 2275000 | 0.0258 | *AR* |
| 12 | 2225000 | 2275000 | 0.0258 | *CARD14* |
| 12 | 2225000 | 2275000 | 0.0258 | *EIF4A3* |
| 12 | 2250000 | 2300000 | 0.0228 | *AR* |
| 12 | 2250000 | 2300000 | 0.0228 | *CARD14* |
| 12 | 2250000 | 2300000 | 0.0228 | *EIF4A3* |
| 12 | 2250000 | 2300000 | 0.0228 | *GAA* |
| 12 | 2350000 | 2400000 | 0.0307 | *GAA* |
| 12 | 2375000 | 2425000 | 0.0301 | *CCDC40* |
| 12 | 2375000 | 2425000 | 0.0301 | *GAA* |
| 12 | 2400000 | 2450000 | 0.0153 | *CCDC40* |
| 12 | 2400000 | 2450000 | 0.0153 | *GAA* |
| 12 | 2525000 | 2575000 | 0.0101 | *TBC1D16* |
| 12 | 2575000 | 2625000 | 0.0096 | *CBX2* |
| 12 | 2575000 | 2625000 | 0.0096 | *CBX4* |
| 12 | 2600000 | 2650000 | 0.0128 | *CBX2* |
| 12 | 2600000 | 2650000 | 0.0128 | *CBX8* |
| 12 | 17500000 | 17550000 | 0.0099 | *NSF* |
| 12 | 31650000 | 31700000 | 0.0116 | *TOM1L1* |
| 12 | 31675000 | 31725000 | 0.0129 | *TOM1L1* |
| 12 | 31700000 | 31750000 | 0.0160 | *COX11* |
| 12 | 31700000 | 31750000 | 0.0160 | *TOM1L1* |
| 12 | 31775000 | 31825000 | 0.0157 | *STXBP4* |
| 12 | 31875000 | 31925000 | 0.0152 | *STXBP4* |
| 13 | 2775000 | 2825000 | 0.0103 | *BTD* |
| 13 | 2775000 | 2825000 | 0.0103 | *HACL1* |
| 13 | 2800000 | 2850000 | 0.0110 | *BTD* |
| 13 | 2800000 | 2850000 | 0.0110 | *HACL1* |
| 13 | 24600000 | 24650000 | 0.0194 | *ITGA9* |
| 13 | 57975000 | 58025000 | 0.0098 | *FOXP1* |
| 13 | 62975000 | 63025000 | 0.0124 | *CHL1* |
| 13 | 65950000 | 66000000 | 0.0119 | *IL5RA* |
| 13 | 84925000 | 84975000 | 0.0115 | *STAG1* |
| 13 | 106100000 | 106150000 | 0.0111 | *RSRC1* |
| 13 | 112675000 | 112725000 | 0.0100 | *SI* |
| 13 | 112725000 | 112775000 | 0.0123 | *SI* |
| 13 | 112750000 | 112800000 | 0.0120 | *SI* |
| 13 | 120000000 | 120050000 | 0.0208 | *NCEH1* |
| 13 | 120025000 | 120075000 | 0.0195 | *NCEH1* |
| 13 | 130000000 | 130050000 | 0.0105 | *ATP11B* |
| 13 | 132125000 | 132175000 | 0.0128 | *MAG* |
| 13 | 132125000 | 132175000 | 0.0128 | *MAGEF1* |
| 13 | 132425000 | 132475000 | 0.0276 | *C3ORF70* |
| 13 | 132450000 | 132500000 | 0.0218 | *C3ORF70* |
| 13 | 132575000 | 132625000 | 0.0115 | *C3ORF70* |
| 13 | 132600000 | 132650000 | 0.0121 | *EHHADH* |
| 13 | 135100000 | 135150000 | 0.0116 | *LPP* |
| 13 | 136025000 | 136075000 | 0.0125 | *TPRG1* |
| 13 | 136050000 | 136100000 | 0.0194 | *TPRG1* |
| 13 | 146750000 | 146800000 | 0.0101 | *SEC22A* |
| 13 | 159125000 | 159175000 | 0.0110 | *PHLDB2* |
| 13 | 159125000 | 159175000 | 0.0110 | *PLCXD2* |
| 13 | 160500000 | 160550000 | 0.0105 | *CD47* |
| 14 | 33700000 | 33750000 | 0.0105 | *ANAPC7* |
| 14 | 33725000 | 33775000 | 0.0102 | *C12ORF24* |
| 14 | 33725000 | 33775000 | 0.0102 | *GPN3* |
| 14 | 53800000 | 53850000 | 0.0103 | *HIC2* |
| 14 | 53800000 | 53850000 | 0.0103 | *PI4KA* |
| 14 | 53825000 | 53875000 | 0.0106 | *PI4KA* |
| 14 | 53850000 | 53900000 | 0.0115 | *PI4KA* |
| 14 | 53850000 | 53900000 | 0.0115 | *SERPIND1* |
| 14 | 53875000 | 53925000 | 0.0117 | *PI4KA* |
| 14 | 53875000 | 53925000 | 0.0117 | *SERPIND1* |
| 14 | 53900000 | 53950000 | 0.0127 | *PI4KA* |
| 14 | 53900000 | 53950000 | 0.0127 | *SNAP29* |
| 14 | 53925000 | 53975000 | 0.0154 | *PI4KA* |
| 14 | 53925000 | 53975000 | 0.0154 | *SNAP29* |
| 14 | 53950000 | 54000000 | 0.0135 | *CRKL* |
| 14 | 53950000 | 54000000 | 0.0135 | *SNAP29* |
| 14 | 53975000 | 54025000 | 0.0114 | *AIFM3* |
| 14 | 53975000 | 54025000 | 0.0114 | *CRKL* |
| 14 | 98225000 | 98275000 | 0.0133 | *AR* |
| 14 | 98225000 | 98275000 | 0.0133 | *PARG* |
| 14 | 98250000 | 98300000 | 0.0148 | *AR* |
| 14 | 98250000 | 98300000 | 0.0148 | *PARG* |
| 14 | 143625000 | 143675000 | 0.0126 | *PLEKHG2* |
| 14 | 143625000 | 143675000 | 0.0126 | *RPS16* |
| 14 | 143625000 | 143675000 | 0.0126 | *RPS16P1* |
| 14 | 143625000 | 143675000 | 0.0126 | *RPS16P10* |
| 14 | 143650000 | 143700000 | 0.0106 | *MED29* |
| 14 | 143650000 | 143700000 | 0.0106 | *PAF1* |
| 14 | 143650000 | 143700000 | 0.0106 | *PLEKHG2* |
| 14 | 143650000 | 143700000 | 0.0106 | *RPS16* |
| 14 | 143650000 | 143700000 | 0.0106 | *RPS16P1* |
| 14 | 143650000 | 143700000 | 0.0106 | *RPS16P10* |
| 14 | 143650000 | 143700000 | 0.0106 | *SAMD4B* |
| 14 | 143650000 | 143700000 | 0.0106 | *ZFP36* |
| 14 | 143725000 | 143775000 | 0.0110 | *GMFG* |
| 14 | 143725000 | 143775000 | 0.0110 | *LRFN1* |
| 14 | 143750000 | 143800000 | 0.0116 | *GMFG* |
| 14 | 143750000 | 143800000 | 0.0116 | *IL28A* |
| 14 | 143750000 | 143800000 | 0.0116 | *IL29* |
| 14 | 143750000 | 143800000 | 0.0116 | *LRFN1* |
| 14 | 143775000 | 143825000 | 0.0140 | *IL28A* |
| 14 | 143775000 | 143825000 | 0.0140 | *IL29* |
| 14 | 143775000 | 143825000 | 0.0140 | *LRFN1* |
| 15 | 21675000 | 21725000 | 0.0109 | *NCKAP5* |
| 15 | 97225000 | 97275000 | 0.0101 | *PDE1A* |
| 15 | 101475000 | 101525000 | 0.0096 | *ZC3H15* |
| 15 | 101500000 | 101550000 | 0.0150 | *ZC3H15* |
| 15 | 101525000 | 101575000 | 0.0105 | *ITGAV* |
| 15 | 101525000 | 101575000 | 0.0105 | *ZC3H15* |
| 15 | 115675000 | 115725000 | 0.0099 | *CLK1* |
| 15 | 115675000 | 115725000 | 0.0099 | *NIF3L1* |
| 15 | 115675000 | 115725000 | 0.0099 | *PPIL3* |
| 15 | 116150000 | 116200000 | 0.0097 | *AR* |
| 15 | 116150000 | 116200000 | 0.0097 | *CASP10* |
| 15 | 116150000 | 116200000 | 0.0097 | *CFLAR* |
| 15 | 117200000 | 117250000 | 0.0119 | *SUMO1* |
| 15 | 117200000 | 117250000 | 0.0119 | *SUMO1P3* |
| 15 | 117225000 | 117275000 | 0.0114 | *SUMO1* |
| 15 | 117225000 | 117275000 | 0.0114 | *SUMO1P3* |
| 15 | 117300000 | 117350000 | 0.0109 | *SUMO1* |
| 15 | 117300000 | 117350000 | 0.0109 | *SUMO1P3* |
| 15 | 117900000 | 117950000 | 0.0096 | *ALS2CR8* |
| 15 | 136825000 | 136875000 | 0.0101 | *EPHA4* |
| 15 | 137825000 | 137875000 | 0.0104 | *AR* |
| 15 | 137825000 | 137875000 | 0.0104 | *FARSB* |
| 15 | 137825000 | 137875000 | 0.0104 | *SGPP2* |
| 15 | 137850000 | 137900000 | 0.0121 | *AR* |
| 15 | 137850000 | 137900000 | 0.0121 | *FARSB* |
| 15 | 137850000 | 137900000 | 0.0121 | *SGPP2* |
| 16 | 350000 | 400000 | 0.0124 | *CTNND2* |
| 16 | 800000 | 850000 | 0.0138 | *CTNND2* |
| 16 | 46350000 | 46400000 | 0.0114 | *RGS7* |
| 16 | 46350000 | 46400000 | 0.0114 | *RGS7BP* |
| 16 | 46600000 | 46650000 | 0.0113 | *CWC27* |
| 16 | 47775000 | 47825000 | 0.0121 | *ERBB2IP* |
| 16 | 55650000 | 55700000 | 0.0123 | *ERGIC1* |
| 16 | 60375000 | 60425000 | 0.0104 | *ODZ2* |
| 16 | 74075000 | 74125000 | 0.0100 | *MRPL22* |
| 16 | 74100000 | 74150000 | 0.0118 | *MRPL22* |
| 16 | 74300000 | 74350000 | 0.0097 | *AR* |
| 16 | 74300000 | 74350000 | 0.0097 | *C5ORF4* |
| 16 | 74300000 | 74350000 | 0.0097 | *LARP1* |
| 16 | 74375000 | 74425000 | 0.0113 | *AR* |
| 16 | 74375000 | 74425000 | 0.0113 | *LARP1* |
| 16 | 78050000 | 78100000 | 0.0102 | *ANXA6* |
| 16 | 78050000 | 78100000 | 0.0102 | *CCDC69* |
| 16 | 85775000 | 85825000 | 0.0117 | *LPCAT1* |
| 16 | 85800000 | 85850000 | 0.0161 | *LPCAT1* |
| 16 | 86675000 | 86725000 | 0.0101 | *DAP* |
| 16 | 86700000 | 86750000 | 0.0108 | *DAP* |
| 17 | 38200000 | 38250000 | 0.0100 | *SI* |
| 17 | 38200000 | 38250000 | 0.0100 | *SIRPB2* |
| 17 | 41050000 | 41100000 | 0.0169 | *COMMD7* |
| 17 | 41075000 | 41125000 | 0.0167 | *COMMD7* |
| 17 | 41250000 | 41300000 | 0.0099 | *COMMD7* |
| 17 | 41250000 | 41300000 | 0.0099 | *DNMT3B* |
| 17 | 46500000 | 46550000 | 0.0100 | *RPRD1B* |
| 17 | 47050000 | 47100000 | 0.0104 | *ADIG* |
| 17 | 47050000 | 47100000 | 0.0104 | *AR* |
| 17 | 47050000 | 47100000 | 0.0104 | *ARHGAP40* |
| 17 | 47050000 | 47100000 | 0.0104 | *RALGAPB* |
| 17 | 61850000 | 61900000 | 0.0130 | *CYP24A1* |
| 18 | 725000 | 775000 | 0.0100 | *VIPR2* |
| 18 | 725000 | 775000 | 0.0100 | *WDR60* |
| 18 | 750000 | 800000 | 0.0104 | *WDR60* |
| 18 | 775000 | 825000 | 0.0117 | *ESYT2* |
| 18 | 775000 | 825000 | 0.0117 | *WDR60* |
| 18 | 10550000 | 10600000 | 0.0122 | *HIPK2* |
| X | 17500000 | 17550000 | 0.0107 | *CXORF23* |
| X | 31950000 | 32000000 | 0.0096 | *DMD* |
| X | 38000000 | 38050000 | 0.0098 | *RPGR* |
| X | 38000000 | 38050000 | 0.0098 | *SRPX* |
| X | 40400000 | 40450000 | 0.0137 | *CXORF38* |
| X | 40425000 | 40475000 | 0.0116 | *CXORF38* |
| X | 40425000 | 40475000 | 0.0116 | *MED14* |
| X | 40975000 | 41025000 | 0.0105 | *USP9X* |
| X | 41000000 | 41050000 | 0.0106 | *USP9X* |
| X | 41025000 | 41075000 | 0.0162 | *USP9X* |
| X | 41050000 | 41100000 | 0.0144 | *USP9X* |
| X | 43325000 | 43375000 | 0.0096 | *MAOB* |
| X | 43400000 | 43450000 | 0.0111 | *MAOB* |
| X | 44100000 | 44150000 | 0.0135 | *FUNDC1* |
| X | 44725000 | 44775000 | 0.0159 | *CXORF36* |
| X | 44750000 | 44800000 | 0.0140 | *CXORF36* |
| X | 44775000 | 44825000 | 0.0113 | *CXORF36* |
| X | 44800000 | 44850000 | 0.0114 | *CXORF36* |
| X | 60425000 | 60475000 | 0.0131 | *AR* |
| X | 65850000 | 65900000 | 0.0118 | *CITED1* |
| X | 65850000 | 65900000 | 0.0118 | *PIN4* |
| X | 65850000 | 65900000 | 0.0118 | *RPS4X* |
| X | 65850000 | 65900000 | 0.0118 | *RPS4XP13* |
| X | 65850000 | 65900000 | 0.0118 | *RPS4XP6* |
| X | 65900000 | 65950000 | 0.0118 | *HDAC8* |
| X | 66800000 | 66850000 | 0.0171 | *PABPC1L2A* |
| X | 66800000 | 66850000 | 0.0171 | *PABPC1L2B* |
| X | 67025000 | 67075000 | 0.0171 | *CHIC1* |
| X | 74000000 | 74050000 | 0.0097 | *BRWD3* |
| X | 74025000 | 74075000 | 0.0097 | *BRWD3* |
| X | 78000000 | 78050000 | 0.0139 | *APOOL* |
| X | 78325000 | 78375000 | 0.0168 | *POF1B* |
| X | 78750000 | 78800000 | 0.0101 | *CHM* |
| X | 91000000 | 91050000 | 0.0171 | *BTK* |
| X | 91000000 | 91050000 | 0.0171 | *TIMM8A* |
| X | 92700000 | 92750000 | 0.0111 | *FAM133A* |
| X | 97425000 | 97475000 | 0.0098 | *BEX5* |
| X | 97425000 | 97475000 | 0.0098 | *TCEAL2* |
| X | 97425000 | 97475000 | 0.0098 | *TCEAL6* |
| X | 97450000 | 97500000 | 0.0098 | *BEX5* |
| X | 97450000 | 97500000 | 0.0098 | *TCEAL6* |
| X | 102625000 | 102675000 | 0.0122 | *GRAMD4* |
| X | 102625000 | 102675000 | 0.0122 | *TSC22D3* |
| X | 103150000 | 103200000 | 0.0140 | *COL4A6* |
| X | 104100000 | 104150000 | 0.0163 | *RGAG1* |
| X | 104350000 | 104400000 | 0.0098 | *CHRDL1* |
| X | 104375000 | 104425000 | 0.0138 | *CHRDL1* |
| X | 113375000 | 113425000 | 0.0115 | *NDUFA1* |
| X | 113375000 | 113425000 | 0.0115 | *RNF113A* |
| X | 113375000 | 113425000 | 0.0115 | *UPF3B* |
| X | 113400000 | 113450000 | 0.0105 | *RPL39* |
| X | 113400000 | 113450000 | 0.0105 | *RPL39P10* |
| X | 113400000 | 113450000 | 0.0105 | *RPL39P13* |
| X | 113400000 | 113450000 | 0.0105 | *RPL39P20* |
| X | 113400000 | 113450000 | 0.0105 | *RPL39P27* |
| X | 113400000 | 113450000 | 0.0105 | *RPL39P32* |
| X | 113400000 | 113450000 | 0.0105 | *UPF3B* |
| X | 114825000 | 114875000 | 0.0119 | *KLHL13* |
| X | 114900000 | 114950000 | 0.0121 | *KLHL13* |
| X | 116025000 | 116075000 | 0.0104 | *THOC2* |
| X | 118775000 | 118825000 | 0.0219 | *CXORF64* |
| X | 118800000 | 118850000 | 0.0104 | *CXORF64* |
| X | 120275000 | 120325000 | 0.0097 | *ACTRT1* |
| X | 122450000 | 122500000 | 0.0141 | *AIFM1* |
| X | 122450000 | 122500000 | 0.0141 | *RAB33A* |
| X | 122475000 | 122525000 | 0.0215 | *AIFM1* |
| X | 122475000 | 122525000 | 0.0215 | *RAB33A* |
| X | 122475000 | 122525000 | 0.0215 | *ZNF280C* |
| X | 122500000 | 122550000 | 0.0108 | *ZNF280C* |
| X | 124800000 | 124850000 | 0.0140 | *HS6ST2* |
| X | 124825000 | 124875000 | 0.0230 | *HS6ST2* |
| X | 125050000 | 125100000 | 0.0219 | *HS6ST2* |
| X | 125075000 | 125125000 | 0.0354 | *HS6ST2* |
| X | 125100000 | 125150000 | 0.0302 | *USP26* |
| X | 142725000 | 142775000 | 0.0105 | *TMLHE* |
| X | 142825000 | 142875000 | 0.0128 | *CLIC2* |
| X | 142850000 | 142900000 | 0.0097 | *CLIC2* |
| X | 142850000 | 142900000 | 0.0097 | *F8A1* |
| X | 142850000 | 142900000 | 0.0097 | *F8A2* |
| X | 142850000 | 142900000 | 0.0097 | *F8A3* |
| X | 142850000 | 142900000 | 0.0097 | *H2AFB1* |
| X | 142850000 | 142900000 | 0.0097 | *H2AFB2* |
| X | 142850000 | 142900000 | 0.0097 | *H2AFB3* |
| X | 142925000 | 142975000 | 0.0109 | *VBP1* |
| X | 142950000 | 143000000 | 0.0155 | *BRCC3* |
| X | 142975000 | 143025000 | 0.0141 | *BRCC3* |
| X | 143000000 | 143050000 | 0.0187 | *BRCC3* |
| X | 143050000 | 143100000 | 0.0237 | *BRCC3* |
| **EPL_LL** | | | | |
| 1 | 20525000 | 20575000 | 0.0518 | *STXBP5* |
| 1 | 20550000 | 20600000 | 0.0681 | *STXBP5* |
| 1 | 48700000 | 48750000 | 0.0641 | *C6ORF204* |
| 1 | 48725000 | 48775000 | 0.0533 | *C6ORF204* |
| 1 | 49775000 | 49825000 | 0.0522 | *ROS1* |
| 1 | 49800000 | 49850000 | 0.0735 | *ROS1* |
| 1 | 50475000 | 50525000 | 0.1039 | *PTP4A1* |
| 1 | 50500000 | 50550000 | 0.1085 | *PTP4A1* |
| 1 | 50850000 | 50900000 | 0.0659 | *EYS* |
| 1 | 50875000 | 50925000 | 0.0687 | *EYS* |
| 1 | 56850000 | 56900000 | 0.0578 | *FAM135A* |
| 1 | 57025000 | 57075000 | 0.0546 | *SMAP1* |
| 1 | 59650000 | 59700000 | 0.0798 | *KIAA1009* |
| 1 | 59675000 | 59725000 | 0.0841 | *KIAA1009* |
| 1 | 59700000 | 59750000 | 0.0518 | *KIAA1009* |
| 1 | 60950000 | 61000000 | 0.0517 | *SNX14* |
| 1 | 60950000 | 61000000 | 0.0517 | *SYNCRIP* |
| 1 | 61975000 | 62025000 | 0.0684 | *HTR1E* |
| 1 | 62750000 | 62800000 | 0.1086 | *AKIRIN2* |
| 1 | 62750000 | 62800000 | 0.1086 | *ORC3L* |
| 1 | 62775000 | 62825000 | 0.1000 | *AKIRIN2* |
| 1 | 62775000 | 62825000 | 0.1000 | *ORC3L* |
| 1 | 74925000 | 74975000 | 0.0583 | *C6ORF168* |
| 1 | 80700000 | 80750000 | 0.0666 | *POPDC3* |
| 1 | 82900000 | 82950000 | 0.0773 | *SOBP* |
| 1 | 82925000 | 82975000 | 0.0597 | *SOBP* |
| 1 | 83075000 | 83125000 | 0.0521 | *SCML4* |
| 1 | 86375000 | 86425000 | 0.0645 | *AMD1* |
| 1 | 86400000 | 86450000 | 0.0599 | *AMD1* |
| 1 | 86425000 | 86475000 | 0.0753 | *CDK19* |
| 1 | 86450000 | 86500000 | 0.0934 | *CDK19* |
| 1 | 94525000 | 94575000 | 0.0665 | *BTK* |
| 1 | 94525000 | 94575000 | 0.0665 | *IBTK* |
| 1 | 94550000 | 94600000 | 0.0683 | *BTK* |
| 1 | 94550000 | 94600000 | 0.0683 | *IBTK* |
| 1 | 101800000 | 101850000 | 0.0637 | *COL12A1* |
| 1 | 101800000 | 101850000 | 0.0637 | *COX7A2* |
| 1 | 101800000 | 101850000 | 0.0637 | *TMEM30A* |
| 1 | 101825000 | 101875000 | 0.0589 | *COL12A1* |
| 1 | 101825000 | 101875000 | 0.0589 | *TMEM30A* |
| 1 | 101850000 | 101900000 | 0.0512 | *COL12A1* |
| 1 | 101875000 | 101925000 | 0.0517 | *COL12A1* |
| 1 | 103250000 | 103300000 | 0.0942 | *CD109* |
| 1 | 103275000 | 103325000 | 0.0549 | *CD109* |
| 1 | 104225000 | 104275000 | 0.0715 | *SETBP1* |
| 1 | 117675000 | 117725000 | 0.0703 | *SPG21* |
| 1 | 141800000 | 141850000 | 0.0515 | *CASC4* |
| 1 | 141850000 | 141900000 | 0.0618 | *CTDSPL2* |
| 1 | 141950000 | 142000000 | 0.0598 | *CASC4* |
| 1 | 155250000 | 155300000 | 0.1481 | *CERS3* |
| 1 | 155275000 | 155325000 | 0.1197 | *CERS3* |
| 1 | 155300000 | 155350000 | 0.1250 | *CERS3* |
| 1 | 155325000 | 155375000 | 0.0914 | *LINS1* |
| 1 | 155375000 | 155425000 | 0.1006 | *ASB7* |
| 1 | 155375000 | 155425000 | 0.1006 | *LINS1* |
| 1 | 155400000 | 155450000 | 0.0872 | *ASB7* |
| 1 | 155425000 | 155475000 | 0.0731 | *ASB7* |
| 1 | 166050000 | 166100000 | 0.0579 | *FBXO15* |
| 1 | 180025000 | 180075000 | 0.0636 | *NEDD4L* |
| 1 | 180750000 | 180800000 | 0.0537 | *CILP* |
| 1 | 185925000 | 185975000 | 0.0631 | *TLE3* |
| 1 | 185950000 | 186000000 | 0.0665 | *TLE3* |
| 1 | 216250000 | 216300000 | 0.0824 | *SGPP1* |
| 1 | 216275000 | 216325000 | 0.0668 | *SGPP1* |
| 1 | 216550000 | 216600000 | 0.1291 | *PPP2R5E* |
| 1 | 216575000 | 216625000 | 0.1014 | *PPP2R5E* |
| 1 | 216600000 | 216650000 | 0.0678 | *PPP2R5E* |
| 1 | 216625000 | 216675000 | 0.0620 | *PPP2R5E* |
| 1 | 216650000 | 216700000 | 0.0509 | *PPP2R5E* |
| 1 | 216750000 | 216800000 | 0.0912 | *RHOJ* |
| 1 | 216775000 | 216825000 | 0.0691 | *GPHB5* |
| 1 | 216775000 | 216825000 | 0.0691 | *RHOJ* |
| 1 | 233000000 | 233050000 | 0.0542 | *MPDZ* |
| 1 | 233675000 | 233725000 | 0.0686 | *TYRP1* |
| 2 | 7625000 | 7675000 | 0.0519 | *ATL3* |
| 2 | 7625000 | 7675000 | 0.0519 | *HRASLS2* |
| 2 | 7625000 | 7675000 | 0.0519 | *PLA2G16* |
| 2 | 26675000 | 26725000 | 0.0860 | *RAG2* |
| 2 | 26700000 | 26750000 | 0.0673 | *RAG1* |
| 2 | 26700000 | 26750000 | 0.0673 | *RAG2* |
| 2 | 26725000 | 26775000 | 0.0801 | *RAG1* |
| 2 | 26725000 | 26775000 | 0.0801 | *TRAF6* |
| 2 | 26750000 | 26800000 | 0.0871 | *TRAF6* |
| 2 | 26775000 | 26825000 | 0.0532 | *PRR5L* |
| 2 | 26775000 | 26825000 | 0.0532 | *TRAF6* |
| 2 | 26800000 | 26850000 | 0.0588 | *PRR5L* |
| 2 | 26825000 | 26875000 | 0.0521 | *PRR5L* |
| 2 | 26850000 | 26900000 | 0.0579 | *PRR5L* |
| 2 | 26875000 | 26925000 | 0.0509 | *PRR5L* |
| 2 | 27075000 | 27125000 | 0.0812 | *COMMD9* |
| 2 | 27100000 | 27150000 | 0.0507 | *COMMD9* |
| 2 | 27150000 | 27200000 | 0.0588 | *LDLRAD3* |
| 2 | 27300000 | 27350000 | 0.0521 | *LDLRAD3* |
| 2 | 27325000 | 27375000 | 0.0499 | *LDLRAD3* |
| 2 | 27450000 | 27500000 | 0.0951 | *LDLRAD3* |
| 2 | 27475000 | 27525000 | 0.0654 | *LDLRAD3* |
| 2 | 27675000 | 27725000 | 0.0641 | *TRIM44* |
| 2 | 27700000 | 27750000 | 0.0669 | *TRIM44* |
| 2 | 27950000 | 28000000 | 0.0501 | *PAMR1* |
| 2 | 27950000 | 28000000 | 0.0501 | *SLC1A2* |
| 2 | 28025000 | 28075000 | 0.0737 | *SLC1A2* |
| 2 | 28050000 | 28100000 | 0.0662 | *SLC1A2* |
| 2 | 28075000 | 28125000 | 0.0776 | *SLC1A2* |
| 2 | 28100000 | 28150000 | 0.0861 | *SLC1A2* |
| 2 | 28125000 | 28175000 | 0.0701 | *CD44* |
| 2 | 28125000 | 28175000 | 0.0701 | *SLC1A2* |
| 2 | 28150000 | 28200000 | 0.0637 | *CD44* |
| 2 | 28175000 | 28225000 | 0.0620 | *CD44* |
| 2 | 28200000 | 28250000 | 0.0504 | *CD44* |
| 2 | 28350000 | 28400000 | 0.0803 | *PDHX* |
| 2 | 28375000 | 28425000 | 0.0658 | *PDHX* |
| 2 | 28400000 | 28450000 | 0.0569 | *PDHX* |
| 2 | 28625000 | 28675000 | 0.0622 | *EHF* |
| 2 | 28650000 | 28700000 | 0.0655 | *EHF* |
| 2 | 30000000 | 30050000 | 0.0687 | *HIPK3* |
| 2 | 30025000 | 30075000 | 0.0521 | *HIPK3* |
| 2 | 30325000 | 30375000 | 0.0573 | *CSTF3* |
| 2 | 30325000 | 30375000 | 0.0573 | *DEPDC7* |
| 2 | 30325000 | 30375000 | 0.0573 | *TCP11L1* |
| 2 | 30350000 | 30400000 | 0.0542 | *TCP11L1* |
| 2 | 30475000 | 30525000 | 0.0733 | *QSER1* |
| 2 | 30525000 | 30575000 | 0.0720 | *PRRG4* |
| 2 | 30550000 | 30600000 | 0.0794 | *PRRG4* |
| 2 | 30575000 | 30625000 | 0.0995 | *CCDC73* |
| 2 | 30600000 | 30650000 | 0.0933 | *CCDC73* |
| 2 | 30625000 | 30675000 | 0.1031 | *CCDC73* |
| 2 | 30650000 | 30700000 | 0.0983 | *CCDC73* |
| 2 | 30675000 | 30725000 | 0.0858 | *CCDC73* |
| 2 | 30700000 | 30750000 | 0.1166 | *CCDC73* |
| 2 | 30700000 | 30750000 | 0.1166 | *EIF3M* |
| 2 | 30725000 | 30775000 | 0.0974 | *CCDC73* |
| 2 | 30725000 | 30775000 | 0.0974 | *EIF3M* |
| 2 | 30750000 | 30800000 | 0.0742 | *EIF3M* |
| 2 | 30875000 | 30925000 | 0.0644 | *WT1* |
| 2 | 32600000 | 32650000 | 0.0513 | *MPPED2* |
| 2 | 32825000 | 32875000 | 0.1206 | *FSHB* |
| 2 | 32850000 | 32900000 | 0.1034 | *FSHB* |
| 2 | 33025000 | 33075000 | 0.0561 | *KCNA4* |
| 2 | 34625000 | 34675000 | 0.0660 | *METT5D1* |
| 2 | 34650000 | 34700000 | 0.0657 | *METT5D1* |
| 2 | 34775000 | 34825000 | 0.0537 | *METT5D1* |
| 2 | 34850000 | 34900000 | 0.0652 | *KIF18A* |
| 2 | 34875000 | 34925000 | 0.0631 | *KIF18A* |
| 2 | 35175000 | 35225000 | 0.0684 | *BDNF* |
| 2 | 35200000 | 35250000 | 0.0777 | *BDNF* |
| 2 | 35400000 | 35450000 | 0.0620 | *LIN7C* |
| 2 | 35425000 | 35475000 | 0.1013 | *LGR4* |
| 2 | 35425000 | 35475000 | 0.1013 | *LIN7C* |
| 2 | 35450000 | 35500000 | 0.0973 | *LGR4* |
| 2 | 35475000 | 35525000 | 0.0581 | *LGR4* |
| 2 | 35550000 | 35600000 | 0.0715 | *LGR4* |
| 2 | 35575000 | 35625000 | 0.0771 | *CCDC34* |
| 2 | 35575000 | 35625000 | 0.0771 | *LGR4* |
| 2 | 35600000 | 35650000 | 0.0585 | *CCDC34* |
| 2 | 35600000 | 35650000 | 0.0585 | *LGR4* |
| 2 | 35625000 | 35675000 | 0.0545 | *CCDC34* |
| 2 | 35850000 | 35900000 | 0.0712 | *BBOX1* |
| 2 | 36300000 | 36350000 | 0.0688 | *ANO3* |
| 2 | 36300000 | 36350000 | 0.0688 | *MUC15* |
| 2 | 37600000 | 37650000 | 0.0690 | *LUZP2* |
| 2 | 37625000 | 37675000 | 0.0536 | *LUZP2* |
| 2 | 37700000 | 37750000 | 0.0563 | *LUZP2* |
| 2 | 37875000 | 37925000 | 0.0518 | *LUZP2* |
| 2 | 37900000 | 37950000 | 0.0539 | *LUZP2* |
| 2 | 38250000 | 38300000 | 0.0566 | *LUZP2* |
| 2 | 39825000 | 39875000 | 0.0576 | *GAS2* |
| 2 | 39850000 | 39900000 | 0.0549 | *GAS2* |
| 2 | 39875000 | 39925000 | 0.0543 | *GAS2* |
| 2 | 40000000 | 40050000 | 0.0623 | *GAS2* |
| 2 | 40025000 | 40075000 | 0.0506 | *GAS2* |
| 2 | 40050000 | 40100000 | 0.0647 | *FANCF* |
| 2 | 40050000 | 40100000 | 0.0647 | *GAS2* |
| 2 | 40075000 | 40125000 | 0.0651 | *FANCF* |
| 2 | 40350000 | 40400000 | 0.0546 | *ANO5* |
| 2 | 44200000 | 44250000 | 0.0547 | *SERGEF* |
| 2 | 44225000 | 44275000 | 0.0684 | *SERGEF* |
| 2 | 44275000 | 44325000 | 0.0753 | *SERGEF* |
| 2 | 44300000 | 44350000 | 0.0566 | *SERGEF* |
| 2 | 44375000 | 44425000 | 0.0590 | *SERGEF* |
| 2 | 44400000 | 44450000 | 0.0659 | *KCNC1* |
| 2 | 44400000 | 44450000 | 0.0659 | *SERGEF* |
| 2 | 44425000 | 44475000 | 0.0506 | *KCNC1* |
| 2 | 44600000 | 44650000 | 0.0601 | *USH1C* |
| 2 | 44850000 | 44900000 | 0.0528 | *NUCB2* |
| 2 | 45225000 | 45275000 | 0.0659 | *PLEKHA7* |
| 2 | 45250000 | 45300000 | 0.0579 | *PLEKHA7* |
| 2 | 45400000 | 45450000 | 0.0540 | *C11ORF58* |
| 2 | 45400000 | 45450000 | 0.0540 | *LOC645086* |
| 2 | 45400000 | 45450000 | 0.0540 | *PLEKHA7* |
| 2 | 45425000 | 45475000 | 0.0538 | *C11ORF58* |
| 2 | 45425000 | 45475000 | 0.0538 | *LOC645086* |
| 2 | 45600000 | 45650000 | 0.0878 | *SOX6* |
| 2 | 45625000 | 45675000 | 0.0785 | *SOX6* |
| 2 | 46200000 | 46250000 | 0.0519 | *SOX6* |
| 2 | 57425000 | 57475000 | 0.0499 | *C1ORF150* |
| 2 | 67725000 | 67775000 | 0.0616 | *OR7D2* |
| 2 | 72825000 | 72875000 | 0.0554 | *VAV1* |
| 2 | 72850000 | 72900000 | 0.0550 | *VAV1* |
| 2 | 74650000 | 74700000 | 0.0608 | *UHRF1* |
| 2 | 79250000 | 79300000 | 0.0602 | *MIER2* |
| 2 | 79250000 | 79300000 | 0.0602 | *PPAP2C* |
| 2 | 86950000 | 87000000 | 0.0545 | *IQGAP2* |
| 2 | 90500000 | 90550000 | 0.0506 | *THBS4* |
| 2 | 90525000 | 90575000 | 0.0615 | *THBS4* |
| 2 | 93400000 | 93450000 | 0.0578 | *LOC100129118* |
| 2 | 93400000 | 93450000 | 0.0578 | *TMEM167A* |
| 3 | 38350000 | 38400000 | 0.0626 | *C16ORF89* |
| 3 | 38350000 | 38400000 | 0.0626 | *NAGPA* |
| 3 | 38375000 | 38425000 | 0.0685 | *NAGPA* |
| 3 | 38375000 | 38425000 | 0.0685 | *SEC14L5* |
| 3 | 38400000 | 38450000 | 0.0620 | *SEC14L5* |
| 3 | 38475000 | 38525000 | 0.0515 | *PPL* |
| 3 | 38475000 | 38525000 | 0.0515 | *UBN1* |
| 3 | 38500000 | 38550000 | 0.0585 | *PPL* |
| 3 | 38500000 | 38550000 | 0.0585 | *UBN1* |
| 3 | 44325000 | 44375000 | 0.0534 | *FAM120A* |
| 3 | 44325000 | 44375000 | 0.0534 | *PHF2* |
| 3 | 44375000 | 44425000 | 0.0533 | *PHF2* |
| 3 | 44425000 | 44475000 | 0.0581 | *PHF2* |
| 3 | 57750000 | 57800000 | 0.0606 | *TSGA10* |
| 3 | 57775000 | 57825000 | 0.0579 | *TSGA10* |
| 3 | 57800000 | 57850000 | 0.0532 | *TSGA10* |
| 3 | 84875000 | 84925000 | 0.0669 | *PEX13* |
| 3 | 84875000 | 84925000 | 0.0669 | *PUS10* |
| 3 | 92175000 | 92225000 | 0.1093 | *PSME4* |
| 3 | 92350000 | 92400000 | 0.0587 | *PSME4* |
| 3 | 92400000 | 92450000 | 0.0762 | *PSME4* |
| 3 | 92425000 | 92475000 | 0.0643 | *PSME4* |
| 3 | 97800000 | 97850000 | 0.0523 | *GTF2A1L* |
| 3 | 97800000 | 97850000 | 0.0523 | *LHCGR* |
| 3 | 97800000 | 97850000 | 0.0523 | *STON1* |
| 3 | 97800000 | 97850000 | 0.0523 | *STON1* |
| 3 | 97825000 | 97875000 | 0.0517 | *GTF2A1L* |
| 3 | 97825000 | 97875000 | 0.0517 | *LHCGR* |
| 3 | 97825000 | 97875000 | 0.0517 | *STON1* |
| 3 | 97825000 | 97875000 | 0.0517 | *STON1* |
| 3 | 126225000 | 126275000 | 0.0730 | *MATN3* |
| 3 | 126225000 | 126275000 | 0.0730 | *WDR35* |
| 3 | 126250000 | 126300000 | 0.0548 | *WDR35* |
| 4 | 97400000 | 97450000 | 0.0665 | *ITLN1* |
| 4 | 107575000 | 107625000 | 0.0534 | *ARNT* |
| 4 | 120975000 | 121025000 | 0.0524 | *GNAI3* |
| 4 | 120975000 | 121025000 | 0.0524 | *GNAT2* |
| 4 | 121000000 | 121050000 | 0.0499 | *GNAI3* |
| 4 | 121000000 | 121050000 | 0.0499 | *GPR61* |
| 4 | 121075000 | 121125000 | 0.0524 | *AMIGO1* |
| 4 | 121075000 | 121125000 | 0.0524 | *ATXN7L2* |
| 4 | 121075000 | 121125000 | 0.0524 | *CYB561D1* |
| 4 | 121075000 | 121125000 | 0.0524 | *SYPL2* |
| 4 | 121200000 | 121250000 | 0.0686 | *PSMA5* |
| 4 | 121200000 | 121250000 | 0.0686 | *SORT1* |
| 4 | 121225000 | 121275000 | 0.0688 | *PSMA5* |
| 4 | 121225000 | 121275000 | 0.0688 | *SORT1* |
| 4 | 121250000 | 121300000 | 0.0633 | *CELSR2* |
| 4 | 121250000 | 121300000 | 0.0633 | *PSRC1* |
| 4 | 121250000 | 121300000 | 0.0633 | *SORT1* |
| 4 | 122375000 | 122425000 | 0.0511 | *VAV3* |
| 5 | 1025000 | 1075000 | 0.0681 | *ATXN10* |
| 5 | 1025000 | 1075000 | 0.0681 | *FBLN1* |
| 5 | 1050000 | 1100000 | 0.0678 | *FBLN1* |
| 5 | 1100000 | 1150000 | 0.0590 | *FBLN1* |
| 5 | 18325000 | 18375000 | 0.0618 | *KRT5* |
| 5 | 18325000 | 18375000 | 0.0618 | *KRT6B* |
| 6 | 32175000 | 32225000 | 0.0605 | *PHKB* |
| 6 | 32825000 | 32875000 | 0.0513 | *GPT2* |
| 6 | 33025000 | 33075000 | 0.0572 | *C16ORF87* |
| 6 | 33050000 | 33100000 | 0.0615 | *C16ORF87* |
| 6 | 33075000 | 33125000 | 0.0633 | *C16ORF87* |
| 6 | 36750000 | 36800000 | 0.0605 | *TSHZ3* |
| 6 | 36775000 | 36825000 | 0.0551 | *TSHZ3* |
| 6 | 37725000 | 37775000 | 0.0500 | *ZNF507* |
| 6 | 38025000 | 38075000 | 0.0657 | *ANKRD27* |
| 6 | 38025000 | 38075000 | 0.0657 | *TDRD12* |
| 6 | 38325000 | 38375000 | 0.0594 | *C19ORF40* |
| 6 | 38325000 | 38375000 | 0.0594 | *RHPN2* |
| 6 | 38325000 | 38375000 | 0.0594 | *RHPN2P1* |
| 6 | 38800000 | 38850000 | 0.0618 | *PEPD* |
| 6 | 38850000 | 38900000 | 0.0824 | *PEPD* |
| 6 | 39450000 | 39500000 | 0.0719 | *KIAA0355* |
| 6 | 39450000 | 39500000 | 0.0719 | *PDCD2L* |
| 6 | 39475000 | 39525000 | 0.0670 | *GPI* |
| 6 | 39475000 | 39525000 | 0.0670 | *PDCD2L* |
| 6 | 39925000 | 39975000 | 0.0503 | *ZNF599* |
| 6 | 39925000 | 39975000 | 0.0503 | *ZNF792* |
| 6 | 39950000 | 40000000 | 0.0509 | *AMD1* |
| 6 | 39950000 | 40000000 | 0.0509 | *GRAMD1A* |
| 6 | 39950000 | 40000000 | 0.0509 | *ZNF792* |
| 6 | 40225000 | 40275000 | 0.0508 | *DAP* |
| 6 | 40225000 | 40275000 | 0.0508 | *DMKN* |
| 6 | 40225000 | 40275000 | 0.0508 | *KRTDAP* |
| 6 | 40300000 | 40350000 | 0.0756 | *CD22* |
| 6 | 40300000 | 40350000 | 0.0756 | *FFAR1* |
| 6 | 40300000 | 40350000 | 0.0756 | *FFAR3* |
| 6 | 40325000 | 40375000 | 0.0817 | *CD22* |
| 6 | 40325000 | 40375000 | 0.0817 | *FFAR1* |
| 6 | 40325000 | 40375000 | 0.0817 | *FFAR3* |
| 6 | 40325000 | 40375000 | 0.0817 | *GAPDHS* |
| 6 | 40325000 | 40375000 | 0.0817 | *MAG* |
| 6 | 40350000 | 40400000 | 0.0663 | *ATP4A* |
| 6 | 40350000 | 40400000 | 0.0663 | *CD22* |
| 6 | 40350000 | 40400000 | 0.0663 | *GAPDHS* |
| 6 | 40350000 | 40400000 | 0.0663 | *MAG* |
| 6 | 40350000 | 40400000 | 0.0663 | *SBSN* |
| 6 | 40400000 | 40450000 | 0.0646 | *ATP4A* |
| 6 | 40425000 | 40475000 | 0.0706 | *ATP4A* |
| 6 | 40450000 | 40500000 | 0.0646 | *ATP4A* |
| 6 | 40600000 | 40650000 | 0.0717 | *ARHGAP33* |
| 6 | 40600000 | 40650000 | 0.0717 | *C19ORF55* |
| 6 | 40600000 | 40650000 | 0.0717 | *HSPB6* |
| 6 | 40600000 | 40650000 | 0.0717 | *LIN37* |
| 6 | 40600000 | 40650000 | 0.0717 | *MLL4* |
| 6 | 40600000 | 40650000 | 0.0717 | *NPHS1* |
| 6 | 40600000 | 40650000 | 0.0717 | *PSENEN* |
| 6 | 40600000 | 40650000 | 0.0717 | *TMEM149* |
| 6 | 40600000 | 40650000 | 0.0717 | *U2AF1L4* |
| 6 | 40600000 | 40650000 | 0.0717 | *ZBTB32* |
| 6 | 40625000 | 40675000 | 0.0653 | *APLP1* |
| 6 | 40625000 | 40675000 | 0.0653 | *ARHGAP33* |
| 6 | 40625000 | 40675000 | 0.0653 | *C19ORF55* |
| 6 | 40625000 | 40675000 | 0.0653 | *HSPB6* |
| 6 | 40625000 | 40675000 | 0.0653 | *KIRREL2* |
| 6 | 40625000 | 40675000 | 0.0653 | *LIN37* |
| 6 | 40625000 | 40675000 | 0.0653 | *NPHS1* |
| 6 | 40625000 | 40675000 | 0.0653 | *PRODH2* |
| 6 | 40625000 | 40675000 | 0.0653 | *PSENEN* |
| 6 | 40625000 | 40675000 | 0.0653 | *U2AF1L4* |
| 6 | 40725000 | 40775000 | 0.0562 | *LRFN3* |
| 6 | 40750000 | 40800000 | 0.0498 | *ALKBH6* |
| 6 | 40750000 | 40800000 | 0.0498 | *C19ORF46* |
| 6 | 40750000 | 40800000 | 0.0498 | *CLIP3* |
| 6 | 40750000 | 40800000 | 0.0498 | *LRFN3* |
| 6 | 40750000 | 40800000 | 0.0498 | *SDHAF1* |
| 6 | 40975000 | 41025000 | 0.0738 | *ZNF146* |
| 6 | 40975000 | 41025000 | 0.0738 | *ZNF260* |
| 6 | 41125000 | 41175000 | 0.0505 | *ZNF567* |
| 6 | 41150000 | 41200000 | 0.0521 | *ZNF461* |
| 6 | 41150000 | 41200000 | 0.0521 | *ZNF567* |
| 6 | 41175000 | 41225000 | 0.0574 | *ZNF345* |
| 6 | 41175000 | 41225000 | 0.0574 | *ZNF382* |
| 6 | 41175000 | 41225000 | 0.0574 | *ZNF461* |
| 6 | 41175000 | 41225000 | 0.0574 | *ZNF829* |
| 6 | 42000000 | 42050000 | 0.0728 | *ZNF571* |
| 6 | 42000000 | 42050000 | 0.0728 | *ZNF790* |
| 6 | 42125000 | 42175000 | 0.0550 | *ZFP3* |
| 6 | 42125000 | 42175000 | 0.0550 | *ZFP30* |
| 6 | 42150000 | 42200000 | 0.0673 | *ZFP3* |
| 6 | 42150000 | 42200000 | 0.0673 | *ZFP30* |
| 6 | 42150000 | 42200000 | 0.0673 | *ZNF790* |
| 6 | 42175000 | 42225000 | 0.0805 | *ZFP3* |
| 6 | 42175000 | 42225000 | 0.0805 | *ZFP30* |
| 6 | 42175000 | 42225000 | 0.0805 | *ZNF790* |
| 6 | 42225000 | 42275000 | 0.0630 | *ZNF607* |
| 6 | 42500000 | 42550000 | 0.0607 | *SIPA1L3* |
| 6 | 42525000 | 42575000 | 0.0659 | *SIPA1L3* |
| 6 | 42575000 | 42625000 | 0.0554 | *DPF1* |
| 6 | 42725000 | 42775000 | 0.0505 | *CATSPERG* |
| 6 | 42725000 | 42775000 | 0.0505 | *KCNK6* |
| 6 | 42725000 | 42775000 | 0.0505 | *YIF1B* |
| 6 | 42750000 | 42800000 | 0.0603 | *CATSPERG* |
| 6 | 42750000 | 42800000 | 0.0603 | *GGN* |
| 6 | 42750000 | 42800000 | 0.0603 | *PSMD8* |
| 6 | 42775000 | 42825000 | 0.0570 | *CATSPERG* |
| 6 | 42775000 | 42825000 | 0.0570 | *FAM98C* |
| 6 | 42775000 | 42825000 | 0.0570 | *GGN* |
| 6 | 42775000 | 42825000 | 0.0570 | *PSMD8* |
| 6 | 42775000 | 42825000 | 0.0570 | *RASGRP4* |
| 6 | 42775000 | 42825000 | 0.0570 | *SPRED3* |
| 6 | 42825000 | 42875000 | 0.0509 | *RASGRP4* |
| 6 | 42825000 | 42875000 | 0.0509 | *RYR1* |
| 6 | 43125000 | 43175000 | 0.0600 | *CAPN12* |
| 6 | 43125000 | 43175000 | 0.0600 | *ECH1* |
| 6 | 43125000 | 43175000 | 0.0600 | *LGALS7* |
| 6 | 43125000 | 43175000 | 0.0600 | *LGALS7B* |
| 6 | 43150000 | 43200000 | 0.0670 | *ECH1* |
| 6 | 43150000 | 43200000 | 0.0670 | *HNRNPL* |
| 6 | 43150000 | 43200000 | 0.0670 | *LGALS7* |
| 6 | 43150000 | 43200000 | 0.0670 | *LGALS7B* |
| 6 | 43150000 | 43200000 | 0.0670 | *LOC644390* |
| 6 | 43175000 | 43225000 | 0.0723 | *ECH1* |
| 6 | 43175000 | 43225000 | 0.0723 | *HNRNPL* |
| 6 | 43175000 | 43225000 | 0.0723 | *LOC644390* |
| 6 | 43175000 | 43225000 | 0.0723 | *RINL* |
| 6 | 43175000 | 43225000 | 0.0723 | *SIRT2* |
| 6 | 43200000 | 43250000 | 0.0555 | *LGALS4* |
| 6 | 43200000 | 43250000 | 0.0555 | *NFKBIB* |
| 6 | 43200000 | 43250000 | 0.0555 | *RINL* |
| 6 | 43200000 | 43250000 | 0.0555 | *SIRT2* |
| 6 | 43375000 | 43425000 | 0.0505 | *PAPL* |
| 6 | 43775000 | 43825000 | 0.0892 | *DLL3* |
| 6 | 43800000 | 43850000 | 0.0877 | *DLL3* |
| 6 | 43800000 | 43850000 | 0.0877 | *SUPT5H* |
| 6 | 43800000 | 43850000 | 0.0877 | *TIMM50* |
| 6 | 43825000 | 43875000 | 0.0889 | *SUPT5H* |
| 6 | 43825000 | 43875000 | 0.0889 | *TIMM50* |
| 6 | 43850000 | 43900000 | 0.1038 | *SUPT5H* |
| 6 | 43900000 | 43950000 | 0.1004 | *CLC* |
| 6 | 43900000 | 43950000 | 0.1004 | *LGALS13* |
| 6 | 43900000 | 43950000 | 0.1004 | *LGALS14* |
| 6 | 43925000 | 43975000 | 0.0887 | *CLC* |
| 6 | 43925000 | 43975000 | 0.0887 | *EID2* |
| 6 | 43925000 | 43975000 | 0.0887 | *EID2B* |
| 6 | 43925000 | 43975000 | 0.0887 | *LGALS13* |
| 6 | 43925000 | 43975000 | 0.0887 | *LGALS14* |
| 6 | 43950000 | 44000000 | 0.0753 | *EID2* |
| 6 | 43950000 | 44000000 | 0.0753 | *EID2B* |
| 6 | 43950000 | 44000000 | 0.0753 | *SELV* |
| 6 | 43975000 | 44025000 | 0.0741 | *LEUTX* |
| 6 | 43975000 | 44025000 | 0.0741 | *SELV* |
| 6 | 44000000 | 44050000 | 0.0762 | *LEUTX* |
| 6 | 44075000 | 44125000 | 0.1010 | *CGB* |
| 6 | 44075000 | 44125000 | 0.1010 | *FCGBP* |
| 6 | 44100000 | 44150000 | 0.0834 | *CGB* |
| 6 | 44100000 | 44150000 | 0.0834 | *FCGBP* |
| 6 | 44175000 | 44225000 | 0.0502 | *CGB* |
| 6 | 44175000 | 44225000 | 0.0502 | *DYRK1B* |
| 6 | 44175000 | 44225000 | 0.0502 | *FCGBP* |
| 6 | 44175000 | 44225000 | 0.0502 | *ZNF546* |
| 6 | 44175000 | 44225000 | 0.0502 | *ZNF780A* |
| 6 | 44175000 | 44225000 | 0.0502 | *ZNF780B* |
| 6 | 44200000 | 44250000 | 0.0578 | *ZNF780A* |
| 6 | 44200000 | 44250000 | 0.0578 | *ZNF780B* |
| 6 | 44225000 | 44275000 | 0.0515 | *ZNF780B* |
| 6 | 44475000 | 44525000 | 0.0561 | *BLVRB* |
| 6 | 44475000 | 44525000 | 0.0561 | *PLD3* |
| 6 | 44475000 | 44525000 | 0.0561 | *SPTBN4* |
| 6 | 44500000 | 44550000 | 0.0597 | *PLD3* |
| 6 | 44500000 | 44550000 | 0.0597 | *SPTBN4* |
| 6 | 44550000 | 44600000 | 0.0525 | *LTBP4* |
| 6 | 44575000 | 44625000 | 0.1011 | *ADCK4* |
| 6 | 44575000 | 44625000 | 0.1011 | *LTBP4* |
| 6 | 44600000 | 44650000 | 0.1078 | *ADCK4* |
| 6 | 44600000 | 44650000 | 0.1078 | *C19ORF54* |
| 6 | 44600000 | 44650000 | 0.1078 | *ITPKC* |
| 6 | 44600000 | 44650000 | 0.1078 | *LTBP4* |
| 6 | 44625000 | 44675000 | 0.0991 | *C19ORF54* |
| 6 | 44625000 | 44675000 | 0.0991 | *ITPKC* |
| 6 | 44625000 | 44675000 | 0.0991 | *MIA* |
| 6 | 44625000 | 44675000 | 0.0991 | *RAB4B* |
| 6 | 44625000 | 44675000 | 0.0991 | *SNRPA* |
| 6 | 44650000 | 44700000 | 0.1072 | *C19ORF54* |
| 6 | 44650000 | 44700000 | 0.1072 | *EGLN2* |
| 6 | 44650000 | 44700000 | 0.1072 | *MIA* |
| 6 | 44650000 | 44700000 | 0.1072 | *RAB4B* |
| 6 | 44650000 | 44700000 | 0.1072 | *SNRPA* |
| 6 | 44675000 | 44725000 | 0.0984 | *ADCK4* |
| 6 | 44675000 | 44725000 | 0.0984 | *CYP2F1* |
| 6 | 44675000 | 44725000 | 0.0984 | *EGLN2* |
| 6 | 44675000 | 44725000 | 0.0984 | *NUMBL* |
| 6 | 44675000 | 44725000 | 0.0984 | *RAB4B* |
| 6 | 44700000 | 44750000 | 0.1035 | *ADCK4* |
| 6 | 44700000 | 44750000 | 0.1035 | *CYP2F1* |
| 6 | 44700000 | 44750000 | 0.1035 | *NUMBL* |
| 6 | 44725000 | 44775000 | 0.1210 | *NUMBL* |
| 6 | 44750000 | 44800000 | 0.1141 | *LTBP4* |
| 6 | 44750000 | 44800000 | 0.1141 | *SHKBP1* |
| 6 | 44875000 | 44925000 | 0.0906 | *CYP2A13* |
| 6 | 44875000 | 44925000 | 0.0906 | *CYP2A6* |
| 6 | 44875000 | 44925000 | 0.0906 | *CYP2A7* |
| 6 | 44875000 | 44925000 | 0.0906 | *CYP2F1* |
| 6 | 44900000 | 44950000 | 0.1181 | *CYP2B6* |
| 6 | 44900000 | 44950000 | 0.1181 | *CYP2F1* |
| 6 | 44900000 | 44950000 | 0.1181 | *CYP2S1* |
| 6 | 44925000 | 44975000 | 0.0829 | *CYP2B6* |
| 6 | 44925000 | 44975000 | 0.0829 | *CYP2F1* |
| 6 | 44925000 | 44975000 | 0.0829 | *CYP2S1* |
| 6 | 44950000 | 45000000 | 0.0610 | *AXL* |
| 6 | 44950000 | 45000000 | 0.0610 | *CYP2B6* |
| 6 | 44975000 | 45025000 | 0.0623 | *AXL* |
| 6 | 44975000 | 45025000 | 0.0623 | *CYP2F1* |
| 6 | 45025000 | 45075000 | 0.0714 | *TGFB1* |
| 6 | 45050000 | 45100000 | 0.1009 | *HNRNPUL1* |
| 6 | 45050000 | 45100000 | 0.1009 | *TGFB1* |
| 6 | 45075000 | 45125000 | 0.1077 | *CCDC9* |
| 6 | 45075000 | 45125000 | 0.1077 | *CCDC97* |
| 6 | 45075000 | 45125000 | 0.1077 | *HNRNPUL1* |
| 6 | 45100000 | 45150000 | 0.0669 | *B9D2* |
| 6 | 45100000 | 45150000 | 0.0669 | *CCDC9* |
| 6 | 45100000 | 45150000 | 0.0669 | *CCDC97* |
| 6 | 45100000 | 45150000 | 0.0669 | *HNRNPUL1* |
| 6 | 45100000 | 45150000 | 0.0669 | *TGFB1* |
| 6 | 45425000 | 45475000 | 0.0659 | *CNFN* |
| 6 | 45425000 | 45475000 | 0.0659 | *LIPE* |
| 6 | 45425000 | 45475000 | 0.0659 | *MEGF8* |
| 6 | 45450000 | 45500000 | 0.0667 | *CNFN* |
| 6 | 45450000 | 45500000 | 0.0667 | *LIPE* |
| 6 | 45450000 | 45500000 | 0.0667 | *MEGF8* |
| 6 | 45475000 | 45525000 | 0.0788 | *MEGF8* |
| 6 | 45475000 | 45525000 | 0.0788 | *TMEM145* |
| 6 | 45500000 | 45550000 | 0.1080 | *CIC* |
| 6 | 45500000 | 45550000 | 0.1080 | *MEGF8* |
| 6 | 45500000 | 45550000 | 0.1080 | *PAFAH1B3* |
| 6 | 45500000 | 45550000 | 0.1080 | *PRR19* |
| 6 | 45500000 | 45550000 | 0.1080 | *TMEM145* |
| 6 | 45525000 | 45575000 | 0.1294 | *CIC* |
| 6 | 45525000 | 45575000 | 0.1294 | *PAFAH1B3* |
| 6 | 45525000 | 45575000 | 0.1294 | *PRR19* |
| 6 | 45550000 | 45600000 | 0.1075 | *ERF* |
| 6 | 45575000 | 45625000 | 0.1224 | *ERF* |
| 6 | 45575000 | 45625000 | 0.1224 | *GSK3A* |
| 6 | 45575000 | 45625000 | 0.1224 | *ZNF526* |
| 6 | 45600000 | 45650000 | 0.1271 | *DEDD2* |
| 6 | 45600000 | 45650000 | 0.1271 | *ERF* |
| 6 | 45600000 | 45650000 | 0.1271 | *GSK3A* |
| 6 | 45600000 | 45650000 | 0.1271 | *ZNF526* |
| 6 | 45625000 | 45675000 | 0.1114 | *DEDD2* |
| 6 | 45850000 | 45900000 | 0.0659 | *GRIK5* |
| 6 | 45850000 | 45900000 | 0.0659 | *POU2F2* |
| 6 | 45975000 | 46025000 | 0.0748 | *CXCL1* |
| 6 | 45975000 | 46025000 | 0.0748 | *CXCL17* |
| 6 | 45975000 | 46025000 | 0.0748 | *LIPE* |
| 6 | 46000000 | 46050000 | 0.0685 | *CD79A* |
| 6 | 46025000 | 46075000 | 0.0561 | *CD79A* |
| 6 | 46025000 | 46075000 | 0.0561 | *DMRTC2* |
| 6 | 46025000 | 46075000 | 0.0561 | *RPS19* |
| 6 | 46025000 | 46075000 | 0.0561 | *RPS19P3* |
| 6 | 46100000 | 46150000 | 0.0553 | *CD177* |
| 6 | 46100000 | 46150000 | 0.0553 | *ETHE1* |
| 6 | 46100000 | 46150000 | 0.0553 | *TEX101* |
| 6 | 46125000 | 46175000 | 0.0947 | *ETHE1* |
| 6 | 46125000 | 46175000 | 0.0947 | *TEX101* |
| 6 | 46125000 | 46175000 | 0.0947 | *XRCC1* |
| 6 | 46125000 | 46175000 | 0.0947 | *ZNF575* |
| 6 | 46150000 | 46200000 | 0.1185 | *IRGQ* |
| 6 | 46150000 | 46200000 | 0.1185 | *XRCC1* |
| 6 | 46150000 | 46200000 | 0.1185 | *ZNF575* |
| 6 | 46150000 | 46200000 | 0.1185 | *ZNF576* |
| 6 | 46175000 | 46225000 | 0.1113 | *CADM4* |
| 6 | 46175000 | 46225000 | 0.1113 | *IRGQ* |
| 6 | 46175000 | 46225000 | 0.1113 | *SRRM5* |
| 6 | 46175000 | 46225000 | 0.1113 | *XRCC1* |
| 6 | 46175000 | 46225000 | 0.1113 | *ZNF428* |
| 6 | 46175000 | 46225000 | 0.1113 | *ZNF576* |
| 6 | 46200000 | 46250000 | 0.0770 | *CADM4* |
| 6 | 46200000 | 46250000 | 0.0770 | *ETHE1* |
| 6 | 46200000 | 46250000 | 0.0770 | *PHLDB3* |
| 6 | 46200000 | 46250000 | 0.0770 | *SRRM5* |
| 6 | 46200000 | 46250000 | 0.0770 | *ZNF428* |
| 6 | 46225000 | 46275000 | 0.0879 | *ETHE1* |
| 6 | 46225000 | 46275000 | 0.0879 | *LYPD3* |
| 6 | 46225000 | 46275000 | 0.0879 | *PHLDB3* |
| 6 | 46250000 | 46300000 | 0.1181 | *LYPD3* |
| 6 | 46250000 | 46300000 | 0.1181 | *PHLDB3* |
| 6 | 46325000 | 46375000 | 0.1077 | *PLAUR* |
| 6 | 46350000 | 46400000 | 0.1023 | *PLAUR* |
| 6 | 46375000 | 46425000 | 0.0678 | *C19ORF61* |
| 6 | 46375000 | 46425000 | 0.0678 | *IRGC* |
| 6 | 46400000 | 46450000 | 0.0549 | *C19ORF61* |
| 6 | 46400000 | 46450000 | 0.0549 | *IRGC* |
| 6 | 46400000 | 46450000 | 0.0549 | *KCNN4* |
| 6 | 46600000 | 46650000 | 0.0618 | *ZNF221* |
| 6 | 46600000 | 46650000 | 0.0618 | *ZNF222* |
| 6 | 46600000 | 46650000 | 0.0618 | *ZNF224* |
| 6 | 46600000 | 46650000 | 0.0618 | *ZNF227* |
| 6 | 46600000 | 46650000 | 0.0618 | *ZNF234* |
| 6 | 46600000 | 46650000 | 0.0618 | *ZNF284* |
| 6 | 46600000 | 46650000 | 0.0618 | *ZNF285* |
| 6 | 46600000 | 46650000 | 0.0618 | *ZNF285B* |
| 6 | 46600000 | 46650000 | 0.0618 | *ZNF45* |
| 6 | 46600000 | 46650000 | 0.0618 | *ZNF806* |
| 6 | 46625000 | 46675000 | 0.0661 | *ZNF155* |
| 6 | 46625000 | 46675000 | 0.0661 | *ZNF221* |
| 6 | 46625000 | 46675000 | 0.0661 | *ZNF223* |
| 6 | 46625000 | 46675000 | 0.0661 | *ZNF224* |
| 6 | 46625000 | 46675000 | 0.0661 | *ZNF225* |
| 6 | 46625000 | 46675000 | 0.0661 | *ZNF226* |
| 6 | 46625000 | 46675000 | 0.0661 | *ZNF230* |
| 6 | 46625000 | 46675000 | 0.0661 | *ZNF234* |
| 6 | 46625000 | 46675000 | 0.0661 | *ZNF284* |
| 6 | 46625000 | 46675000 | 0.0661 | *ZNF285* |
| 6 | 46625000 | 46675000 | 0.0661 | *ZNF285B* |
| 6 | 46625000 | 46675000 | 0.0661 | *ZNF45* |
| 6 | 46625000 | 46675000 | 0.0661 | *ZNF806* |
| 6 | 46775000 | 46825000 | 0.0505 | *ZFP112* |
| 6 | 46775000 | 46825000 | 0.0505 | *ZNF229* |
| 6 | 46775000 | 46825000 | 0.0505 | *ZNF230* |
| 6 | 46800000 | 46850000 | 0.0554 | *ZNF180* |
| 6 | 46800000 | 46850000 | 0.0554 | *ZNF229* |
| 6 | 46825000 | 46875000 | 0.0516 | *CEACAM20* |
| 6 | 46825000 | 46875000 | 0.0516 | *ZNF180* |
| 6 | 46825000 | 46875000 | 0.0516 | *ZNF229* |
| 6 | 47075000 | 47125000 | 0.0568 | *CEACAM16* |
| 6 | 47200000 | 47250000 | 0.0744 | *BCAM* |
| 6 | 47200000 | 47250000 | 0.0744 | *PVRL2* |
| 6 | 47225000 | 47275000 | 0.0721 | *APOE* |
| 6 | 47225000 | 47275000 | 0.0721 | *LOC100129500* |
| 6 | 47225000 | 47275000 | 0.0721 | *PVRL2* |
| 6 | 47225000 | 47275000 | 0.0721 | *TOMM40* |
| 6 | 47250000 | 47300000 | 0.0532 | *APOC1* |
| 6 | 47250000 | 47300000 | 0.0532 | *APOC2* |
| 6 | 47250000 | 47300000 | 0.0532 | *APOC4* |
| 6 | 47250000 | 47300000 | 0.0532 | *APOE* |
| 6 | 47250000 | 47300000 | 0.0532 | *CLPTM1* |
| 6 | 47250000 | 47300000 | 0.0532 | *LOC100129500* |
| 6 | 47250000 | 47300000 | 0.0532 | *PVRL2* |
| 6 | 47250000 | 47300000 | 0.0532 | *TOMM40* |
| 6 | 47275000 | 47325000 | 0.0967 | *APOC1* |
| 6 | 47275000 | 47325000 | 0.0967 | *APOC2* |
| 6 | 47275000 | 47325000 | 0.0967 | *APOC4* |
| 6 | 47275000 | 47325000 | 0.0967 | *CLPTM1* |
| 6 | 47275000 | 47325000 | 0.0967 | *TOMM40* |
| 6 | 47300000 | 47350000 | 0.0961 | *CLPTM1* |
| 6 | 47300000 | 47350000 | 0.0961 | *PVRL2* |
| 6 | 47300000 | 47350000 | 0.0961 | *RELB* |
| 6 | 47300000 | 47350000 | 0.0961 | *TOMM40* |
| 6 | 47375000 | 47425000 | 0.0725 | *RSPH6A* |
| 6 | 47375000 | 47425000 | 0.0725 | *SYMPK* |
| 6 | 47400000 | 47450000 | 0.0904 | *FOXA3* |
| 6 | 47400000 | 47450000 | 0.0904 | *RSPH6A* |
| 6 | 47400000 | 47450000 | 0.0904 | *SYMPK* |
| 6 | 47425000 | 47475000 | 0.0790 | *FOXA3* |
| 6 | 47425000 | 47475000 | 0.0790 | *IRF2BP1* |
| 6 | 47425000 | 47475000 | 0.0790 | *MYPOP* |
| 6 | 47425000 | 47475000 | 0.0790 | *SYMPK* |
| 6 | 47450000 | 47500000 | 0.0663 | *FOXA3* |
| 6 | 47450000 | 47500000 | 0.0663 | *IRF2BP1* |
| 6 | 47450000 | 47500000 | 0.0663 | *MYPOP* |
| 6 | 47450000 | 47500000 | 0.0663 | *NANOS2* |
| 6 | 47475000 | 47525000 | 0.0549 | *NANOS2* |
| 6 | 47475000 | 47525000 | 0.0549 | *NOVA2* |
| 6 | 48275000 | 48325000 | 0.0529 | *ZC3H4* |
| 6 | 48300000 | 48350000 | 0.0504 | *SAE1* |
| 6 | 48300000 | 48350000 | 0.0504 | *ZC3H4* |
| 6 | 48325000 | 48375000 | 0.0502 | *SAE1* |
| 6 | 48350000 | 48400000 | 0.0698 | *SAE1* |
| 6 | 48375000 | 48425000 | 0.0682 | *BBC3* |
| 6 | 48375000 | 48425000 | 0.0682 | *SAE1* |
| 6 | 48400000 | 48450000 | 0.0669 | *BBC3* |
| 6 | 48400000 | 48450000 | 0.0669 | *CCDC9* |
| 6 | 48400000 | 48450000 | 0.0669 | *SAE1* |
| 6 | 48425000 | 48475000 | 0.0629 | *CCDC9* |
| 6 | 48425000 | 48475000 | 0.0629 | *PRR24* |
| 6 | 48700000 | 48750000 | 0.0565 | *NAPA* |
| 6 | 48725000 | 48775000 | 0.0531 | *KPTN* |
| 6 | 48725000 | 48775000 | 0.0531 | *NAPA* |
| 6 | 48775000 | 48825000 | 0.0533 | *KPTN* |
| 6 | 48775000 | 48825000 | 0.0533 | *SLC8A2* |
| 6 | 48925000 | 48975000 | 0.0532 | *EHD2* |
| 6 | 48925000 | 48975000 | 0.0532 | *GLTSCR1* |
| 6 | 48925000 | 48975000 | 0.0532 | *GLTSCR2* |
| 6 | 48925000 | 48975000 | 0.0532 | *LOC440311* |
| 6 | 49050000 | 49100000 | 0.0707 | *CRX* |
| 6 | 49050000 | 49100000 | 0.0707 | *TPRX1* |
| 6 | 49075000 | 49125000 | 0.0796 | *CRX* |
| 6 | 49075000 | 49125000 | 0.0796 | *SULT2A1* |
| 6 | 49075000 | 49125000 | 0.0796 | *TPRX1* |
| 6 | 49100000 | 49150000 | 0.0579 | *SULT2A1* |
| 6 | 49125000 | 49175000 | 0.0589 | *BSPH1* |
| 6 | 49125000 | 49175000 | 0.0589 | *ELSPBP1* |
| 6 | 49200000 | 49250000 | 0.0581 | *CABP5* |
| 6 | 49200000 | 49250000 | 0.0581 | *LIG1* |
| 6 | 49225000 | 49275000 | 0.0699 | *LIG1* |
| 6 | 49400000 | 49450000 | 0.0525 | *SYNGR4* |
| 6 | 49400000 | 49450000 | 0.0525 | *TMEM143* |
| 6 | 49700000 | 49750000 | 0.0583 | *CA11* |
| 6 | 49700000 | 49750000 | 0.0583 | *DBP* |
| 6 | 49700000 | 49750000 | 0.0583 | *FAM83E* |
| 6 | 49700000 | 49750000 | 0.0583 | *RPL18* |
| 6 | 49700000 | 49750000 | 0.0583 | *SPACA4* |
| 6 | 49700000 | 49750000 | 0.0583 | *SPHK2* |
| 6 | 49700000 | 49750000 | 0.0583 | *SULT2B1* |
| 6 | 49775000 | 49825000 | 0.0545 | *FUT2* |
| 6 | 49775000 | 49825000 | 0.0545 | *IZUMO1* |
| 6 | 49775000 | 49825000 | 0.0545 | *MAMSTR* |
| 6 | 49775000 | 49825000 | 0.0545 | *RASIP1* |
| 6 | 49800000 | 49850000 | 0.0635 | *FGF21* |
| 6 | 49800000 | 49850000 | 0.0635 | *FUT1* |
| 6 | 49800000 | 49850000 | 0.0635 | *IZUMO1* |
| 6 | 49800000 | 49850000 | 0.0635 | *MAMSTR* |
| 6 | 49800000 | 49850000 | 0.0635 | *RASIP1* |
| 6 | 49825000 | 49875000 | 0.0547 | *BCAT2* |
| 6 | 49825000 | 49875000 | 0.0547 | *FGF21* |
| 6 | 49825000 | 49875000 | 0.0547 | *FUT1* |
| 6 | 49825000 | 49875000 | 0.0547 | *HSD17B14* |
| 6 | 50000000 | 50050000 | 0.0524 | *C19ORF73* |
| 6 | 50000000 | 50050000 | 0.0524 | *KCNA7* |
| 6 | 50000000 | 50050000 | 0.0524 | *LIN7B* |
| 6 | 50000000 | 50050000 | 0.0524 | *PPFIA3* |
| 6 | 50000000 | 50050000 | 0.0524 | *SNRNP70* |
| 6 | 50025000 | 50075000 | 0.0822 | *CGB* |
| 6 | 50025000 | 50075000 | 0.0822 | *CGB1* |
| 6 | 50025000 | 50075000 | 0.0822 | *CGB2* |
| 6 | 50025000 | 50075000 | 0.0822 | *CGB5* |
| 6 | 50025000 | 50075000 | 0.0822 | *CGB7* |
| 6 | 50025000 | 50075000 | 0.0822 | *CGB8* |
| 6 | 50025000 | 50075000 | 0.0822 | *KCNA7* |
| 6 | 50025000 | 50075000 | 0.0822 | *LHB* |
| 6 | 50025000 | 50075000 | 0.0822 | *NTF4* |
| 6 | 50025000 | 50075000 | 0.0822 | *RUVBL2* |
| 6 | 50025000 | 50075000 | 0.0822 | *SNRNP70* |
| 6 | 50050000 | 50100000 | 0.0883 | *CGB* |
| 6 | 50050000 | 50100000 | 0.0883 | *CGB1* |
| 6 | 50050000 | 50100000 | 0.0883 | *CGB2* |
| 6 | 50050000 | 50100000 | 0.0883 | *CGB5* |
| 6 | 50050000 | 50100000 | 0.0883 | *CGB7* |
| 6 | 50050000 | 50100000 | 0.0883 | *CGB8* |
| 6 | 50050000 | 50100000 | 0.0883 | *FTL* |
| 6 | 50050000 | 50100000 | 0.0883 | *FTLP2* |
| 6 | 50050000 | 50100000 | 0.0883 | *GYS1* |
| 6 | 50050000 | 50100000 | 0.0883 | *LHB* |
| 6 | 50050000 | 50100000 | 0.0883 | *NTF4* |
| 6 | 50050000 | 50100000 | 0.0883 | *RUVBL2* |
| 6 | 50075000 | 50125000 | 0.0653 | *BAX* |
| 6 | 50075000 | 50125000 | 0.0653 | *DHDH* |
| 6 | 50075000 | 50125000 | 0.0653 | *FTL* |
| 6 | 50075000 | 50125000 | 0.0653 | *FTLP2* |
| 6 | 50075000 | 50125000 | 0.0653 | *GYS1* |
| 6 | 50075000 | 50125000 | 0.0653 | *NUCB1* |
| 6 | 50075000 | 50125000 | 0.0653 | *RUVBL2* |
| 6 | 50100000 | 50150000 | 0.0528 | *BAX* |
| 6 | 50100000 | 50150000 | 0.0528 | *DHDH* |
| 6 | 50100000 | 50150000 | 0.0528 | *NUCB1* |
| 6 | 50100000 | 50150000 | 0.0528 | *TULP2* |
| 6 | 57625000 | 57675000 | 0.0579 | *A1BG* |
| 6 | 57625000 | 57675000 | 0.0579 | *ZNF837* |
| 6 | 57625000 | 57675000 | 0.0579 | *ZSCAN22* |
| 7 | 1650000 | 1700000 | 0.0610 | *MYLK4* |
| 7 | 91800000 | 91850000 | 0.0732 | *RGMA* |
| 7 | 91825000 | 91875000 | 0.0596 | *RGMA* |
| 7 | 103400000 | 103450000 | 0.0521 | *ABCD4* |
| 7 | 104775000 | 104825000 | 0.0519 | *TTLL5* |
| 8 | 3750000 | 3800000 | 0.0783 | *SORCS2* |
| 8 | 3775000 | 3825000 | 0.0576 | *SORCS2* |
| 8 | 3925000 | 3975000 | 0.0574 | *SORCS2* |
| 8 | 4050000 | 4100000 | 0.0515 | *AFAP1* |
| 8 | 4050000 | 4100000 | 0.0515 | *SORCS2* |
| 8 | 5725000 | 5775000 | 0.0588 | *OTOP1* |
| 8 | 5725000 | 5775000 | 0.0588 | *TMEM128* |
| 8 | 5750000 | 5800000 | 0.0570 | *LYAR* |
| 8 | 5750000 | 5800000 | 0.0570 | *TMEM128* |
| 8 | 5800000 | 5850000 | 0.0625 | *ZBTB49* |
| 8 | 5925000 | 5975000 | 0.0583 | *ZNF518B* |
| 8 | 6125000 | 6175000 | 0.0545 | *CLNK* |
| 8 | 12550000 | 12600000 | 0.0840 | *NCAPG* |
| 8 | 12575000 | 12625000 | 0.0688 | *LCORL* |
| 8 | 12575000 | 12625000 | 0.0688 | *NCAPG* |
| 8 | 12600000 | 12650000 | 0.0584 | *LCORL* |
| 8 | 12600000 | 12650000 | 0.0584 | *NCAPG* |
| 8 | 12625000 | 12675000 | 0.0632 | *LCORL* |
| 8 | 50025000 | 50075000 | 0.1315 | *ETFDH* |
| 8 | 50025000 | 50075000 | 0.1315 | *PPID* |
| 8 | 50050000 | 50100000 | 0.0980 | *ETFDH* |
| 8 | 50050000 | 50100000 | 0.0980 | *PPID* |
| 8 | 53750000 | 53800000 | 0.0549 | *FSTL5* |
| 8 | 74200000 | 74250000 | 0.0526 | *CXCL1* |
| 8 | 79950000 | 80000000 | 0.0517 | *KIAA0922* |
| 8 | 83400000 | 83450000 | 0.0553 | *LRBA* |
| 8 | 89500000 | 89550000 | 0.0629 | *GAB1* |
| 8 | 89525000 | 89575000 | 0.0553 | *GAB1* |
| 8 | 89750000 | 89800000 | 0.0510 | *USP38* |
| 8 | 89775000 | 89825000 | 0.0698 | *USP38* |
| 8 | 146025000 | 146075000 | 0.0513 | *PRKG2* |
| 9 | 22500000 | 22550000 | 0.0534 | *CCDC81* |
| 9 | 22525000 | 22575000 | 0.0886 | *CCDC81* |
| 9 | 22550000 | 22600000 | 0.0617 | *CCDC81* |
| 9 | 22550000 | 22600000 | 0.0617 | *ME3* |
| 9 | 83050000 | 83100000 | 0.0694 | *DYNC1I1* |
| 9 | 97000000 | 97050000 | 0.0514 | *HDAC9* |
| 9 | 97025000 | 97075000 | 0.0986 | *HDAC9* |
| 9 | 106375000 | 106425000 | 0.0549 | *SEMA3A* |
| 9 | 113700000 | 113750000 | 0.0610 | *FBXL13* |
| 10 | 26325000 | 26375000 | 0.0503 | *PTPRC* |
| 11 | 86225000 | 86275000 | 0.0547 | *MCF2L* |
| 11 | 86250000 | 86300000 | 0.0625 | *MCF2L* |
| 12 | 6200000 | 6250000 | 0.0523 | *KCTD2* |
| 12 | 11225000 | 11275000 | 0.0521 | *MAP2K6* |
| 12 | 18250000 | 18300000 | 0.0522 | *ARL17A* |
| 12 | 18250000 | 18300000 | 0.0522 | *ARL17B* |
| 12 | 18250000 | 18300000 | 0.0522 | *LOC646030* |
| 12 | 18250000 | 18300000 | 0.0522 | *LRRC37A* |
| 12 | 18250000 | 18300000 | 0.0522 | *LRRC37B* |
| 12 | 50175000 | 50225000 | 0.0545 | *SMG6* |
| 12 | 53200000 | 53250000 | 0.0503 | *WSCD1* |
| 12 | 53225000 | 53275000 | 0.0528 | *WSCD1* |
| 12 | 53875000 | 53925000 | 0.0574 | *ZFP3* |
| 12 | 53875000 | 53925000 | 0.0574 | *ZNF232* |
| 12 | 54175000 | 54225000 | 0.0505 | *MINK1* |
| 12 | 54175000 | 54225000 | 0.0505 | *PLD2* |
| 12 | 54400000 | 54450000 | 0.0566 | *ARRB2* |
| 12 | 54400000 | 54450000 | 0.0566 | *PELP1* |
| 12 | 54425000 | 54475000 | 0.0564 | *PELP1* |
| 13 | 16550000 | 16600000 | 0.0577 | *ZCWPW2* |
| 13 | 17650000 | 17700000 | 0.0520 | *RBMS3* |
| 13 | 23375000 | 23425000 | 0.0517 | *STAC* |
| 13 | 23575000 | 23625000 | 0.0569 | *MLH1* |
| 13 | 24600000 | 24650000 | 0.0573 | *ITGA9* |
| 13 | 27825000 | 27875000 | 0.0608 | *ULK4* |
| 13 | 28725000 | 28775000 | 0.0599 | *SEC22C* |
| 13 | 28725000 | 28775000 | 0.0599 | *VIPR1* |
| 13 | 28750000 | 28800000 | 0.0622 | *NKTR* |
| 13 | 28750000 | 28800000 | 0.0622 | *SEC22C* |
| 13 | 28750000 | 28800000 | 0.0622 | *SS18L2* |
| 13 | 29050000 | 29100000 | 0.0620 | *FAM198A* |
| 13 | 29050000 | 29100000 | 0.0620 | *LOC26172* |
| 13 | 33375000 | 33425000 | 0.0621 | *PTPN23* |
| 13 | 34650000 | 34700000 | 0.0506 | *IP6K2* |
| 13 | 34650000 | 34700000 | 0.0506 | *PRKAR2A* |
| 13 | 43250000 | 43300000 | 0.0578 | *DNAH12* |
| 13 | 57975000 | 58025000 | 0.0505 | *FOXP1* |
| 13 | 65925000 | 65975000 | 0.0665 | *CNTN4* |
| 13 | 65925000 | 65975000 | 0.0665 | *IL5RA* |
| 13 | 65950000 | 66000000 | 0.0949 | *IL5RA* |
| 13 | 65975000 | 66025000 | 0.0704 | *IL5RA* |
| 13 | 65975000 | 66025000 | 0.0704 | *TRNT1* |
| 13 | 66050000 | 66100000 | 0.0733 | *CRBN* |
| 13 | 66050000 | 66100000 | 0.0733 | *TRNT1* |
| 13 | 66075000 | 66125000 | 0.0628 | *CRBN* |
| 13 | 66075000 | 66125000 | 0.0628 | *TRNT1* |
| 13 | 70150000 | 70200000 | 0.0958 | *GRM7* |
| 13 | 106125000 | 106175000 | 0.0519 | *RSRC1* |
| 13 | 106425000 | 106475000 | 0.0562 | *RSRC1* |
| 13 | 106450000 | 106500000 | 0.0619 | *RSRC1* |
| 13 | 106550000 | 106600000 | 0.0621 | *RSRC1* |
| 13 | 134325000 | 134375000 | 0.0521 | *MASP1* |
| 13 | 136350000 | 136400000 | 0.0547 | *TP63* |
| 13 | 136375000 | 136425000 | 0.0547 | *TP63* |
| 13 | 143825000 | 143875000 | 0.0521 | *MUC4* |
| 13 | 143850000 | 143900000 | 0.0531 | *KIAA0226* |
| 13 | 149925000 | 149975000 | 0.0635 | *PLA1A* |
| 13 | 149925000 | 149975000 | 0.0635 | *POPDC2* |
| 14 | 21950000 | 22000000 | 0.0506 | *PALLD* |
| 14 | 23925000 | 23975000 | 0.0687 | *ZNF84* |
| 14 | 25675000 | 25725000 | 0.0676 | *GPR133* |
| 14 | 27325000 | 27375000 | 0.0570 | *TMEM132D* |
| 14 | 31500000 | 31550000 | 0.0662 | *PITPNM2* |
| 14 | 31525000 | 31575000 | 0.0651 | *ARL6IP4* |
| 14 | 31525000 | 31575000 | 0.0651 | *PITPNM2* |
| 14 | 34650000 | 34700000 | 0.0538 | *ATXN2* |
| 14 | 34650000 | 34700000 | 0.0538 | *SH2B3* |
| 14 | 36400000 | 36450000 | 0.0574 | *SUDS3* |
| 14 | 36425000 | 36475000 | 0.0507 | *SUDS3* |
| 14 | 98475000 | 98525000 | 0.0523 | *LOC10431* |
| 14 | 98475000 | 98525000 | 0.0523 | *PARG* |
| 14 | 98475000 | 98525000 | 0.0523 | *TIMM23B* |
| 14 | 143625000 | 143675000 | 0.0922 | *PLEKHG2* |
| 14 | 143625000 | 143675000 | 0.0922 | *RPS16* |
| 14 | 143625000 | 143675000 | 0.0922 | *RPS16P1* |
| 14 | 143625000 | 143675000 | 0.0922 | *RPS16P10* |
| 14 | 143650000 | 143700000 | 0.0861 | *MED29* |
| 14 | 143650000 | 143700000 | 0.0861 | *PAF1* |
| 14 | 143650000 | 143700000 | 0.0861 | *PLEKHG2* |
| 14 | 143650000 | 143700000 | 0.0861 | *RPS16* |
| 14 | 143650000 | 143700000 | 0.0861 | *RPS16P1* |
| 14 | 143650000 | 143700000 | 0.0861 | *RPS16P10* |
| 14 | 143650000 | 143700000 | 0.0861 | *SAMD4B* |
| 14 | 143650000 | 143700000 | 0.0861 | *ZFP3* |
| 14 | 143650000 | 143700000 | 0.0861 | *ZFP36* |
| 14 | 143675000 | 143725000 | 0.0553 | *MED29* |
| 14 | 143675000 | 143725000 | 0.0553 | *PAF1* |
| 14 | 143675000 | 143725000 | 0.0553 | *SAMD4B* |
| 14 | 143675000 | 143725000 | 0.0553 | *ZFP3* |
| 14 | 143675000 | 143725000 | 0.0553 | *ZFP36* |
| 14 | 143700000 | 143750000 | 0.0639 | *GMFG* |
| 14 | 143700000 | 143750000 | 0.0639 | *SAMD4B* |
| 14 | 143725000 | 143775000 | 0.0729 | *GMFG* |
| 14 | 143725000 | 143775000 | 0.0729 | *LRFN1* |
| 14 | 143750000 | 143800000 | 0.0715 | *GMFG* |
| 14 | 143750000 | 143800000 | 0.0715 | *IL28A* |
| 14 | 143750000 | 143800000 | 0.0715 | *IL29* |
| 14 | 143750000 | 143800000 | 0.0715 | *LRFN1* |
| 14 | 143775000 | 143825000 | 0.0917 | *IL28A* |
| 14 | 143775000 | 143825000 | 0.0917 | *IL29* |
| 14 | 143775000 | 143825000 | 0.0917 | *LRFN1* |
| 15 | 101500000 | 101550000 | 0.0527 | *ZC3H15* |
| 15 | 104475000 | 104525000 | 0.0940 | *WDR75* |
| 15 | 104500000 | 104550000 | 0.0960 | *WDR75* |
| 15 | 104525000 | 104575000 | 0.1000 | *WDR75* |
| 15 | 115700000 | 115750000 | 0.0590 | *BZW1* |
| 15 | 115700000 | 115750000 | 0.0590 | *BZW1L1* |
| 15 | 115700000 | 115750000 | 0.0590 | *BZW1P1* |
| 15 | 115700000 | 115750000 | 0.0590 | *CLK1* |
| 15 | 115700000 | 115750000 | 0.0590 | *PPIL3* |
| 15 | 117275000 | 117325000 | 0.0503 | *SUMO1* |
| 15 | 117275000 | 117325000 | 0.0503 | *SUMO1P3* |
| 15 | 117875000 | 117925000 | 0.0577 | *ALS2CR8* |
| 15 | 117900000 | 117950000 | 0.0530 | *ALS2CR8* |
| 16 | 47850000 | 47900000 | 0.0531 | *ERBB2IP* |
| 16 | 47875000 | 47925000 | 0.0528 | *ERBB2IP* |
| 16 | 47875000 | 47925000 | 0.0528 | *SFRS12* |
| 16 | 47900000 | 47950000 | 0.0793 | *ERBB2IP* |
| 16 | 47900000 | 47950000 | 0.0793 | *SFRS12* |
| 16 | 47925000 | 47975000 | 0.0703 | *SFRS12* |
| 16 | 51150000 | 51200000 | 0.0611 | *CCNB1* |
| 16 | 51150000 | 51200000 | 0.0611 | *SLC30A5* |
| 16 | 51175000 | 51225000 | 0.0523 | *SLC30A5* |
| 16 | 65075000 | 65125000 | 0.0644 | *MAT2B* |
| 16 | 65100000 | 65150000 | 0.0587 | *HMMR* |
| 16 | 65100000 | 65150000 | 0.0587 | *MAT2B* |
| 16 | 67050000 | 67100000 | 0.0682 | *GABRA6* |
| 16 | 86675000 | 86725000 | 0.0565 | *DAP* |
| 16 | 86700000 | 86750000 | 0.0582 | *DAP* |
| 17 | 12875000 | 12925000 | 0.0573 | *SLC20A2* |
| 17 | 41050000 | 41100000 | 0.0724 | *COMMD7* |
| 17 | 41075000 | 41125000 | 0.0521 | *COMMD7* |
| 17 | 43625000 | 43675000 | 0.0524 | *TRPC4AP* |
| 17 | 43850000 | 43900000 | 0.0675 | *UQCC* |
| 17 | 46425000 | 46475000 | 0.0537 | *KIAA0406* |
| 17 | 46425000 | 46475000 | 0.0537 | *VSTM2L* |
| 17 | 47025000 | 47075000 | 0.0534 | *RALGAPB* |
| 17 | 47050000 | 47100000 | 0.0551 | *ADIG* |
| 17 | 47050000 | 47100000 | 0.0551 | *ARHGAP40* |
| 17 | 47050000 | 47100000 | 0.0551 | *RALGAPB* |
| 17 | 53100000 | 53150000 | 0.0816 | *KCNS1* |
| 17 | 53100000 | 53150000 | 0.0816 | *PI3* |
| 17 | 53100000 | 53150000 | 0.0816 | *WFDC12* |
| 17 | 53100000 | 53150000 | 0.0816 | *WFDC5* |
| 17 | 57100000 | 57150000 | 0.0570 | *CSE1L* |
| 17 | 57100000 | 57150000 | 0.0570 | *DDX27* |
| 18 | 10450000 | 10500000 | 0.0514 | *HIPK2* |
| 18 | 10550000 | 10600000 | 0.0776 | *HIPK2* |
| X | 51775000 | 51825000 | 0.0582 | *HUWE1* |
| X | 91000000 | 91050000 | 0.0541 | *BTK* |
| X | 91000000 | 91050000 | 0.0541 | *TIMM8A* |
| X | 92700000 | 92750000 | 0.1271 | *FAM133A* |
| X | 141000000 | 141050000 | 0.0591 | *GABRA3* |
| X | 141050000 | 141100000 | 0.0506 | *GABRA3* |
| X | 141275000 | 141325000 | 0.0537 | *GABRQ* |
| X | 141300000 | 141350000 | 0.0576 | *GABRQ* |
| X | 141300000 | 141350000 | 0.0576 | *MAG* |
| X | 141300000 | 141350000 | 0.0576 | *MAGEA6* |
| X | 141325000 | 141375000 | 0.0610 | *CETN2* |
| X | 141325000 | 141375000 | 0.0610 | *MAG* |
| X | 141325000 | 141375000 | 0.0610 | *MAGEA6* |
| X | 141350000 | 141400000 | 0.0644 | *CETN2* |
| X | 141350000 | 141400000 | 0.0644 | *NSDHL* |
| X | 141375000 | 141425000 | 0.0527 | *CETN2* |
| X | 141375000 | 141425000 | 0.0527 | *NSDHL* |
| X | 142300000 | 142350000 | 0.0507 | *MECP2* |
| X | 143225000 | 143275000 | 0.0850 | *DKC1* |
| X | 143225000 | 143275000 | 0.0850 | *GAB3* |
| X | 143225000 | 143275000 | 0.0850 | *MPP1* |
| X | 143250000 | 143300000 | 0.0710 | *DKC1* |
| X | 143250000 | 143300000 | 0.0710 | *GAB3* |
| X | 143250000 | 143300000 | 0.0710 | *MPP1* |

| **ID** |  | | **Term** | | ***P*-value** | **Corrected *P*-value**^a^ | | **Associated Genes Found** | | |
| --- | --- | --- | --- | --- | --- | --- | --- | --- | --- | --- |
| **GO** |  |  | |  | | |  | |  |  |
| GO:0019373 |  | epoxygenase P450 pathway | | 8.40E-10 | | | 2.20E-08 | | CYP2A13, CYP2A6, CYP2A7, CYP2B6, CYP2F1, CYP2S1 |  |
| GO:0019369 |  | arachidonic acid metabolic process | | 4.30E-07 | | | 1.00E-05 | | CYP2A13, CYP2A6, CYP2A7, CYP2B6, CYP2F1, CYP2S1 |  |
| GO:0033559 |  | unsaturated fatty acid metabolic process | | 7.00E-05 | | | 1.40E-03 | | CYP2A13, CYP2A6, CYP2A7, CYP2B6, CYP2F1, CYP2S1 |  |
| GO:0001676 |  | long-chain fatty acid metabolic process | | 8.10E-07 | | | 1.90E-05 | | CYP2A13, CYP2A6, CYP2A7, CYP2B6, CYP2F1, CYP2S1, LIPE |  |
| GO:0006690 |  | icosanoid metabolic process | | 3.40E-05 | | | 7.90E-04 | | CYP2A13, CYP2A6, CYP2A7, CYP2B6, CYP2F1, CYP2S1 |  |
| GO:1901568 |  | fatty acid derivative metabolic process | | 3.40E-05 | | | 7.90E-04 | | CYP2A13, CYP2A6, CYP2A7, CYP2B6, CYP2F1, CYP2S1 |  |
| GO:0043011 |  | myeloid dendritic cell differentiation | | 1.20E-04 | | | 2.40E-03 | | RELB, TGFB1, TRAF6 |  |
| GO:0097028 |  | dendritic cell differentiation | | 1.40E-03 | | | 1.50E-02 | | RELB, TGFB1, TRAF6 |  |
| GO:0002709 |  | regulation of T cell mediated immunity | | 3.20E-03 | | | 2.20E-02 | | CLC, NECTIN2, TRAF6 |  |
| GO:0043388 |  | positive regulation of DNA binding | | 3.00E-03 | | | 2.40E-02 | | PLAUR, TGFB1, TRAF6 |  |
| GO:0002718 |  | regulation of cytokine production involved in immune response | | 5.00E-04 | | | 7.60E-03 | | CLC, FFAR3, TGFB1, TRAF6 |  |
| GO:0001773 |  | myeloid dendritic cell activation | | 4.40E-04 | | | 7.10E-03 | | RELB, TGFB1, TRAF6 |  |
| GO:0002367 |  | cytokine production involved in immune response | | 1.00E-03 | | | 1.40E-02 | | CLC, FFAR3, TGFB1, TRAF6 |  |
| GO:1903725 |  | regulation of phospholipid metabolic process | | 6.00E-03 | | | 2.40E-02 | | APOC1, APOC2, TGFB1 |  |
| GO:0046503 |  | glycerolipid catabolic process | | 3.70E-03 | | | 2.20E-02 | | APOC1, APOC2, LIPE |  |
| GO:0045806 |  | negative regulation of endocytosis | | 1.80E-03 | | | 1.80E-02 | | APOC1, APOC2, TGFB1 |  |
| GO:0031440 |  | regulation of mRNA 3'-end processing | | 1.60E-04 | | | 3.20E-03 | | PAF1, SNRPA, SUPT5H |  |
| GO:0006378 |  | mRNA polyadenylation | | 1.30E-03 | | | 1.60E-02 | | PAF1, SNRPA, SUPT5H |  |
| GO:0043631 |  | RNA polyadenylation | | 1.40E-03 | | | 1.50E-02 | | PAF1, SNRPA, SUPT5H |  |
| GO:0030512 |  | negative regulation of transforming growth factor beta receptor signaling pathway | | 6.00E-03 | | | 2.40E-02 | | CD109, EID2, TGFB1 |  |
| GO:1903845 |  | negative regulation of cellular response to transforming growth factor beta stimulus | | 6.40E-03 | | | 1.20E-02 | | CD109, EID2, TGFB1 |  |
| GO:0046323 |  | glucose import | | 1.00E-03 | | | 1.40E-02 | | FFAR3, GSK3A, RAB4B, SLC1A2 |  |
| GO:0046324 |  | regulation of glucose import | | 6.20E-03 | | | 1.80E-02 | | FFAR3, GSK3A, SLC1A2 |  |
| GO:0030728 |  | ovulation | | 3.20E-04 | | | 5.80E-03 | | CGB1, CGB2, LHB |  |
| GO:0007292 |  | female gamete generation | | 4.00E-04 | | | 6.80E-03 | | CGB1, CGB2, CGB3, CGB7, LHB |  |
| GO:0006501 |  | C-terminal protein lipidation | | 6.70E-03 | | | 6.70E-03 | | CD109, LYPD3, TEX101 |  |
| GO:1903313 |  | positive regulation of mRNA metabolic process | | 2.30E-03 | | | 2.10E-02 | | PAF1, PRR5L, ZFP36 |  |
| GO:0070231 |  | T cell apoptotic process | | 3.20E-03 | | | 2.20E-02 | | CLC, LGALS13, LGALS14 |  |
| **KEGG** |  |  | |  | | |  | |  |  |
| GO:0000980 |  | Metabolism of xenobiotics by cytochrome P450 | | 4.10E-05 | | | 9.00E-04 | | CYP2A13, CYP2A6, CYP2B6, CYP2F1, CYP2S1 |  |
| GO:0000830 |  | Retinol metabolism | | 4.60E-03 | | | 2.30E-02 | | CYP2A6, CYP2B6, CYP2S1 |  |

**Table S4.**  GO terms and KEGG pathways enriched by 107 common genes of three different grouping methods in the Bamaxiang pigs.

^a^Corrected *P*-values after Bonferroni correction for multiple testing.

**Table S5.** EPL scores and LL levels of different genotypes of candidate genes.

|  | | | | | | | | |
| --- | --- | --- | --- | --- | --- | --- | --- | --- |
| Gene | Genotype | Num^a^ | EPL score | | | LL level | | |
|  |  |  | Mean | Sd | *P*-value^b^ | Mean | Sd | *P*-value^b^ |
| TGFB1 | T/T | 281 | 23.20 | 22.56 | 1.01E-02 | 0.29 | 0.25 | 7.48E-03 |
|  | T/C | 25 | 11.96 | 19.59 |  | 0.16 | 0.17 |  |
| SNRPA | C/C | 296 | 23.32 | 22.50 | 4.35E-04 | 0.28 | 0.25 | 6.01E-02 |
|  | C/T | 17 | 5.05 | 6.73 |  | 0.17 | 0.19 |  |
| TRAF6 | C/C | 297 | 23.22 | 22.50 | 1.13E-03 | 0.29 | 0.25 | 2.57E-03 |
|  | C/G | 16 | 5.75 | 8.05 |  | 0.10 | 0.14 |  |
| CYP2B6 | C/C | 298 | 23.21 | 22.46 | 7.37E-04 | 0.28 | 0.25 | 2.41E-02 |
|  | C/A | 15 | 4.60 | 7.19 |  | 0.14 | 0.14 |  |
| CD44 | GG | 295 | 23.40 | 22.40 | 1.67E-04 | 0.29 | 0.25 | 1.37E-02 |
|  | GA | 18 | 4.20 | 7.20 |  | 0.14 | 0.16 |  |
| CD22 | CC | 296 | 23.32 | 22.50 | 1.28E-03 | 0.28 | 0.25 | 8.72E-02 |
|  | CT | 17 | 5.05 | 6.73 |  | 0.17 | 0.19 |  |
|  | TT | 1 | 2.00 | NA |  | 0.01 | NA |  |
| ^a^ Number of individuals. | | | | | | | | |
| ^b^ *P* value of F test of *genotype* in the model: *phenotype* = *genotype* + *sex* + *batch* +*1* | | | | | | | | |

**Table S6.** EPL scores and LL levels of combined genotypes of two candidate genes.

| Gene | Genotype | Num^a^ | EPL score | | | LL level | | |  |
| --- | --- | --- | --- | --- | --- | --- | --- | --- | --- |
|  |  |  | Mean | Sd | *P*-value^b^ | Mean | Sd | *P*-value^b^ |  |
| *CD22* and *CD44* | GG/CC | 284 | 24.08 | 22.60 | 4.78E-05 | 0.29 | 0.25 | 1.79E-02 |  |
|  | GA/CC or GG/CT | 23 | 6.04 | 7.87 |  | 0.15 | 0.16 |  |  |
|  | GA/CT | 6 | 2.00 | 3.52 |  | 0.18 | 0.22 |  |  |
|  | NA/TT | 1 | 2.00 | NA |  | 0.00 | NA |  |  |
| ^a^ Number of individuals | | | | | | | | | |
| ^b^ *P* value of F test of *genotype* in the model: *phenotype = genotype + sex + batch +1* | | | | | | | | | |

**Table S7.** The expression of 197 genes consisting of 107 common genes and 158 key-node genes in healthy lung tissues and affected lung lesions.

| **Gene** | **Helathy lung^a^** | **Affected lung^b^** | **Log_2_(FC)^c^** | ***P*adj^d^** | **Grouping (Allele^e^, RF^f^, SF^g^)** | | | |
| --- | --- | --- | --- | --- | --- | --- | --- | --- |
|  |  |  |  |  | **F_ST_^h^** | **EPL score** | **LL level** | **EPL & LL** |
| *VAV1* | 29.86 ± 5.01 | 72.12 ± 9.79 | -1.2535 | 3.72E-19 | / | G, 0.0873, 0.0080 | / | / |
| *MPP1* | 43.17 ± 5.34 | 89.45 ± 13.48 | -1.0390 | 7.65E-17 | / | A, 0.0952, 0.0120 | / | A, 0.1282, 0.0074 |
| *CD44* | 42.28 ± 4.35 | 77.94 ± 10.40 | -0.8742 | 5.24E-15 | Fst_intersection | A, 0.1048, 0.0100 | A, 0.0523, 0.0063 | A, 0.1579, 0.0037 |
| *GYS1* | 7.54 ± 0.76 | 13.04 ± 1.15 | -0.7843 | 1.84E-14 | Fst_intersection | T, 0.0873, 0.0160 | / | / |
| *GPI* | 83.26 ± 7.06 | 147.08 ± 23.25 | -0.8134 | 8.74E-13 | / | C, 0.0984, 0.0160 | / | C, 0.1579, 0.0110 |
| *CIC* | 12.71 ± 1.38 | 25.19 ± 5.86 | -0.9723 | 1.71E-11 | Fst_intersection | / | / | / |
| *SUPT5H* | 32.64 ± 1.77 | 47.6 ± 4.93 | -0.5413 | 3.78E-10 | Fst_intersection | / | G, 0.0523, 0.0095 | / |
| *FCGBP* | 2.03 ± 0.44 | 31.22 ± 28.30 | -3.1055 | 3.98E-10 | Fst_intersection | / | / | / |
| *CD22* | 2.37 ± 0.87 | 5.98 ± 0.64 | -1.2938 | 4.71E-10 | Fst_intersection | T, 0.0873, 0.0160 | / | T, 0.1410, 0.0110 |
| *PVRL2* | 33.93 ± 4.18 | 58.2 ± 10.38 | -0.7689 | 7.29E-09 | Fst_intersection | / | / | / |
| *RELB* | 5.77 ± 1.64 | 15.41 ± 5.16 | -1.3607 | 6.59E-09 | Fst_intersection | / | G, 0.0461, 0.0094 | / |
| *EHHADH* | 0.96 ± 0.77 | 30.08 ± 23.73 | -3.3120 | 7.50E-09 | / | / | T, 0.0520, 0.1509 | / |
| *PEPD* | 4.97 ± 0.56 | 7.79 ± 0.63 | -0.6408 | 2.08E-08 | Fst_intersection | C, 0.0887, 0.01606 | / | C, 0.1410, 0.0111 |
| *APOE* | 588.70 ± 145.33 | 1233.88 ± 311.26 | -1.0385 | 1.43E-07 | / | G, 0.0873, 0.0120 | / | G, 0.1410, 0.0110 |
| *BDNF* | 3.50 ± 0.81 | 1.38 ± 0.54 | 1.2867 | 1.73E-07 | / | G, 0.1167, 0.0141 | / | G, 0.1528, 0.0148 |
| *CADM4* | 1.16 ± 0.19 | 2.02 ± 0.21 | -0.7949 | 4.79E-07 | Fst_intersection | / | / | / |
| *AIFM1* | 6.59 ± 0.64 | 10.42 ± 1.87 | -0.6535 | 5.07E-07 | / | / | G, 0.1842, 0.0818 | / |
| *HFM1* | 2.74 ± 0.83 | 0.96 ± 0.50 | 1.4203 | 1.46E-06 | / | C, 0.2097, 0.0344 | / | / |
| *CD79A* | 2.34 ± 1.44 | 11.31 ± 4.96 | -1.9766 | 1.81E-06 | / | / | A, 0.1438, 0.0660 | A, 0.2308, 0.0662 |
| *BCAM* | 172.98 ± 31.97 | 94.59 ± 24.54 | 0.8516 | 1.88E-06 | Fst_intersection | / | / | G, 0.1410, 0.0110 |
| *SLC1A2* | 0.61 ± 0.30 | 1.98 ± 0.87 | -1.5693 | 2.44E-06 | Fst_intersection | / | / | / |
| *F8* | 16.38 ± 3.87 | 8.17 ± 1.97 | 0.9743 | 2.86E-06 | / | T, 0.0635, 0.0061 | / | / |
| *YY1* | 16.95 ± 1.53 | 12.80 ± 0.48 | 0.4025 | 2.07E-05 | / | / | C, 0.3046, 0.4528 | / |
| *LTBP4* | 85.23 ± 9.62 | 47.01 ± 17.63 | 0.8361 | 2.08E-05 | Fst_intersection | / | / | / |
| *TRAF6* | 3.38 ± 0.43 | 5.02 ± 0.22 | -0.5612 | 2.09E-05 | Fst_intersection | G, 0.0726, 0.0140 | G, 0.0490, 0.0031 | G, 0.1184, 0.0037 |
| *DMD* | 4.49 ± 0.96 | 2.58 ± 0.54 | 0.7811 | 2.54E-05 | / | / | T, 0.1678, 0.2394 | / |
| *SAMD4B* | 12.52 ± 0.99 | 16.40 ± 1.12 | -0.3870 | 2.83E-05 | Fst_intersection | / | / | / |
| *PSMA5* | 15.09 ± 1.46 | 21.73 ± 3.24 | -0.5207 | 2.83E-05 | / | / | / | C, 0.0897, 0.0000 |
| *GFPT1* | 13.48 ± 2.18 | 21.21 ± 3.88 | -0.6432 | 3.12E-05 | / | / | G, 0.4118, 0.2987 | / |
| *TRAP1* | 18.80 ± 3.20 | 28.55 ± 3.58 | -0.5943 | 4.82E-05 | / | / | T, 0.3826, 0.2816 | / |
| *SNRPA* | 22.32 ± 2.59 | 31.93 ± 3.99 | -0.5106 | 6.48E-05 | Fst_intersection | T, 0.0726, 0.0160 | T, 0.0458, 0.0094 | T, 0.1184, 0.0110 |
| *FAM133A* | 0.30 ± 0.14 | 0.05 ± 0.04 | 1.9412 | 1.47E-04 | Fst_intersection | / | / | / |
| *ZNF526* | 2.85 ± 0.45 | 4.39 ± 0.42 | -0.6137 | 1.65E-04 | Fst_intersection | / | / | / |
| *PPP2R5E* | 12.65 ± 1.30 | 9.54 ± 0.50 | 0.4040 | 1.79E-04 | / | G, 0.1935, 0.0935 | / | G, 0.2237, 0.0815 |
| *CETN2* | 54.95 ± 12.72 | 34.32 ± 3.97 | 0.6649 | 1.86E-04 | / | / | / | C, 0.3846, 0.1780 |
| *LGALS13* | 3.50 ± 2.66 | 0.36 ± 0.43 | 2.1528 | 2.01E-04 | Fst_intersection | / | / | / |
| *LHB* | 7.92 ± 4.14 | 2.10 ± 1.31 | 1.6311 | 2.18E-04 | Fst_intersection | / | / | / |
| *RHOJ* | 12.81 ± 1.94 | 8.51 ± 1.41 | 0.5802 | 2.25E-04 | / | T, 0.1129, 0.0140 | / | T, 0.1579, 0.01471 |
| *IRAK1* | 13.36 ± 1.97 | 19.61 ± 3.11 | -0.5458 | 2.58E-04 | / | G, 0.0635, 0.0080 | / | / |
| *ADCY9* | 4.37 ± 0.82 | 2.89 ± 0.24 | 0.5874 | 3.14E-04 | / | / | T, 0.3474, 0.2358 | / |
| *ERBB2IP* | 37.23 ± 4.43 | 27.76 ± 2.10 | 0.4197 | 3.47E-04 | Fst_intersection | / | / | / |
| *HCFC1* | 18.06 ± 2.92 | 25.36 ± 2.38 | -0.4837 | 6.85E-04 | / | C, 0.0556, 0.0060 | / | / |
| *TMPRSS11D* | 0.01 ± 0.03 | 0.38 ± 0.36 | -2.0373 | 1.37E-03 | / | / | C, 0.2119, 0.3065 | / |
| *GSK3B* | 17.84 ± 2.11 | 13.92 ± 0.72 | 0.3552 | 1.60E-03 | / | C, 0.3571, 0.2028 | / | / |
| *LRBA* | 7.36 ± 1.75 | 4.68 ± 0.65 | 0.6372 | 1.94E-03 | Fst_intersection | / | / | / |
| *TOMM40* | 18.32 ± 2.74 | 25.56 ± 4.15 | -0.4732 | 2.55E-03 | Fst_intersection | G, 0.0873, 0.0120 | / | G, 0.1410, 0.0110 |
| *C19ORF54* | 7.38 ± 1.29 | 10.84 ± 2.25 | -0.5437 | 3.19E-03 | Fst_intersection | / | / | / |
| *BAX* | 8.99 ± 1.29 | 12.83 ± 2.96 | -0.5043 | 3.25E-03 | / | / | / | A, 0.1538, 0.0147 |
| *HNRNPUL1* | 80.66 ± 6.21 | 97.11 ± 5.80 | -0.2664 | 3.79E-03 | Fst_intersection | / | / | / |
| *KCNN4* | 1.03 ± 0.30 | 0.43 ± 0.22 | 1.1358 | 3.80E-03 | / | / | / | A, 0.1282, 0.0110 |
| *PRR5L* | 12.57 ± 1.95 | 8.50 ± 2.43 | 0.5525 | 3.86E-03 | Fst_intersection | / | / | / |
| *SETBP1* | 3.06 ± 0.36 | 2.14 ± 0.55 | 0.5042 | 3.99E-03 | Fst_intersection | / | / | / |
| *ZFP36* | 0.29 ± 0.20 | 0.05 ± 0.03 | 1.6444 | 6.18E-03 | Fst_intersection | / | / | / |
| *HDAC9* | 1.04 ± 0.38 | 1.86 ± 0.43 | -0.7943 | 9.67E-03 | / | G, 0.2581, 0.1185 | / | G, 0.2949, 0.1029 |
| *RBBP7* | 33.46 ± 3.91 | 26.36 ± 3.51 | 0.3403 | 9.98E-03 | / | C, 0.0476, 0.0000 | / | / |
| *SMARCC1* | 1.83 ± 0.42 | 2.68 ± 0.26 | -0.5377 | 1.13E-02 | / | G, 0.2963, 0.1234 | / | / |
| *TIMM50* | 11.19 ± 1.53 | 14.84 ± 2.61 | -0.4001 | 1.57E-02 | Fst_intersection | / | / | / |
| *CTNNBL1* | 10.72 ± 1.45 | 14.08 ± 1.78 | -0.3868 | 1.85E-02 | / | T, 0.2661, 0.1084 | / | / |
| *HDAC8* | 0.74 ± 0.27 | 1.24 ± 0.13 | -0.7089 | 1.85E-02 | / | / | C, 0.0682, 0.1470 | / |
| *TGFB1* | 50.17 ± 7.57 | 69.35 ± 21.14 | -0.4570 | 1.89E-02 | Fst_intersection | T, 0.0726, 0.0160 | T, 0.0458, 0.0094 | T, 0.1184, 0.0110 |
| *EIF4A3* | 13.77 ± 1.62 | 16.67 ± 0.60 | -0.2734 | 1.89E-02 | / | / | C, 0.3444, 0.4969 | / |
| *STUB1* | 34.99 ± 4.43 | 43.11 ± 3.20 | -0.2982 | 2.04E-02 | / | / | G, 0.0230, 0.0520 | / |
| *HIPK2* | 4.09 ± 0.77 | 5.59 ± 1.11 | -0.4398 | 2.54E-02 | / | / | / | G, 0.2237, 0.0478 |
| *FFAR3* | 0.11 ± 0.10 | 0.41 ± 0.16 | -1.3802 | 2.55E-02 | Fst_intersection | / | / | / |
| *DNAJA3* | 17.48 ± 1.83 | 21.36 ± 2.77 | -0.2865 | 2.92E-02 | / | / | G, 0.3487, 0.2327 | / |
| *PTPRC* | 102.28 ± 16.68 | 130 ± 16.43 | -0.3411 | 3.13E-02 | / | G, 0.0887, 0.0160 | / | G, 0.1184, 0.0147 |
| *EPHA5* | 0.11 ± 0.10 | 0.01 ± 0.02 | 1.4357 | 3.44E-02 | / | / | A, 0.1118, 0.2075 | / |
| *SLIT3* | 16.64 ± 2.37 | 21.51 ± 4.67 | -0.3642 | 3.76E-02 | / | C, 0.3016, 0.1225 | / | / |
| *EXO1* | 0.87 ± 0.38 | 1.52 ± 0.49 | -0.7416 | 3.87E-02 | / | / | G, 0.1141, 0.0641 | / |
| *COMMD9* | 21.01 ± 2.46 | 16.98 ± 2.75 | 0.3032 | 4.14E-02 | Fst_intersection | / | / | / |
| *ERF* | 26.79 ± 3.15 | 20.93 ± 5.17 | 0.3500 | 4.61E-02 | Fst_intersection | / | / | / |
| *CYP2B6* | 44.22 ± 24.65 | 20.24 ± 11.7 | 0.9595 | 4.88E-02 | Fst_intersection | A, 0.0794, 0.0100 | A, 0.0422, 0.0063 | A, 0.1282, 0.0074 |
| *LIPE* | 3.11 ± 0.46 | 3.84 ± 0.23 | -0.2997 | 5.92E-02 | Fst_intersection | / | / | A, 0.1184, 0.0074 |
| *POLR1B* | 9.18 ± 0.89 | 10.58 ± 0.34 | -0.2030 | 7.55E-02 | / | / | A, 0.4967, 0.6038 | / |
| *FTL* | 385.04 ± 52.62 | 501.92 ± 171.85 | -0.3732 | 8.38E-02 | Fst_intersection | / | / | / |
| *ITGAV* | 14.03 ± 2.03 | 11.73 ± 1.01 | 0.2543 | 9.09E-02 | / | / | G, 0.411, 0.5514 | / |
| *BTK* | 1.75 ± 0.65 | 2.55 ± 0.37 | -0.5177 | 9.16E-02 | / | / | T, 0.0487, 0.0031 | / |
| *CLPTM1* | 64.92 ± 6.18 | 73.64 ± 3.33 | -0.1808 | 9.51E-02 | Fst_intersection | / | / | / |
| *UBA6* | 2.56 ± 0.55 | 3.30 ± 0.63 | -0.3596 | 1.03E-01 | / | / | T, 0.1136, 0.2044 | / |
| *GSK3A* | 31.88 ± 3.96 | 37.45 ± 3.42 | -0.2298 | 1.03E-01 | Fst_intersection | / | G, 0.0533, 0.0094 | G, 0.1447, 0.0110 |
| *MEGF8* | 6.48 ± 1.31 | 8.07 ± 1.10 | -0.3102 | 1.06E-01 | Fst_intersection | / | / | / |
| *CREBBP* | 15.11 ± 2.14 | 17.95 ± 1.75 | -0.2458 | 1.09E-01 | / | / | A, 0.3966, 0.2843 | / |
| *NEDD4L* | 8.76 ± 1.00 | 7.14 ± 1.68 | 0.2885 | 1.14E-01 | / | T, 0.1508, 0.0280 | / | T, 0.1923, 0.0221 |
| *CYP2F1* | 0.67 ± 0.33 | 0.42 ± 0.11 | 0.6135 | 1.28E-01 | Fst_intersection | / | / | / |
| *ENO3* | 19.99 ± 2.68 | 24.37 ± 5.73 | -0.2804 | 1.40E-01 | / | G, 0.3145, 0.1498 | / | / |
| *POLR3K* | 12.75 ± 1.09 | 10.94 ± 1.58 | 0.2180 | 1.46E-01 | / | / | G, 0.0171, 0.0521 | / |
| *RSRC1* | 10.08 ± 2.79 | 7.54 ± 1.96 | 0.4015 | 1.49E-01 | Fst_intersection | T, 0.0727, 0.0186 | / | / |
| *WT1* | 0.19 ± 0.27 | 0.58 ± 0.44 | -1.0238 | 1.50E-01 | / | A, 0.1190, 0.0100 | / | A, 0.1667, 0.0074 |
| *ZNF575* | 0.91 ± 0.30 | 0.64 ± 0.13 | 0.4838 | 1.60E-01 | Fst_intersection | / | / | / |
| *KIF18A* | 1.49 ± 0.59 | 2.07 ± 0.49 | -0.4480 | 1.73E-01 | / | C, 0.1111, 0.0121 | / | C, 0.1538, 0.0111 |
| *TMEM145* | 1.05 ± 0.37 | 1.47 ± 0.37 | -0.4527 | 1.81E-01 | Fst_intersection | / | / | / |
| *GTF2B* | 9.91 ± 1.10 | 11.38 ± 1.32 | -0.1975 | 1.98E-01 | / | / | G, 0.1111, 0.2389 | / |
| *NPHS1* | 0.09 ± 0.10 | 0.26 ± 0.21 | -0.9151 | 2.11E-01 | / | / | / | A, 0.1410, 0.0110 |
| *PI3* | 0.00 ± 0.00 | 0.75 ± 0.87 | -0.6171 | 2.29E-01 | / | T, 0.1694, 0.0740 | / | T, 0.2368, 0.0662 |
| *AR* | 0.19 ± 0.16 | 0.09 ± 0.03 | 0.7851 | 2.30E-01 | / | / | G, 0.0662, 0.0203 | / |
| *PLAUR* | 18.50 ± 2.00 | 21.52 ± 5.21 | -0.2149 | 2.66E-01 | Fst_intersection | T, 0.0726, 0.0160 | T, 0.0458, 0.0094 | T, 0.1184, 0.0110 |
| *PLEKHG2* | 0.49 ± 0.13 | 0.62 ± 0.14 | -0.3281 | 2.68E-01 | Fst_intersection | / | / | / |
| *PRKAR2A* | 14.02 ± 2.55 | 16.26 ± 1.92 | -0.2109 | 2.71E-01 | / | G, 0.0887, 0.0200 | / | G, 0.1316, 0.0257 |
| *NSDHL* | 11.40 ± 0.87 | 10.38 ± 0.53 | 0.1348 | 2.72E-01 | / | C, 0.3443, 0.1798 | / | C, 0.3846, 0.1780 |
| *XRCC1* | 12.32 ± 1.53 | 13.87 ± 1.51 | -0.1690 | 2.83E-01 | Fst_intersection | / | / | / |
| *ZNF576* | 5.73 ± 0.78 | 6.76 ± 1.62 | -0.2322 | 2.94E-01 | Fst_intersection | / | / | / |
| *ANAPC7* | 9.90 ± 1.20 | 11.03 ± 0.77 | -0.1537 | 2.95E-01 | / | / | G, 0.1837, 0.1154 | / |
| *GP1BA* | 0.49 ± 0.25 | 0.32 ± 0.13 | 0.5314 | 3.17E-01 | / | G, 0.3145, 0.1498 | / | / |
| *REV3L* | 2.50 ± 0.48 | 2.10 ± 0.41 | 0.2465 | 3.21E-01 | / | / | C, 0.3007, 0.2025 | / |
| *BRCC3* | 3.28 ± 0.58 | 3.82 ± 0.51 | -0.2154 | 3.26E-01 | / | / | C, 0.1164, 0.0503 | / |
| *ETHE1* | 12.83 ± 5.27 | 16.6 ± 5.24 | -0.3498 | 3.45E-01 | Fst_intersection | / | / | / |
| *CD109* | 1.66 ± 0.45 | 1.34 ± 0.51 | 0.2940 | 3.73E-01 | Fst_intersection | / | / | / |
| *CCNB1* | 6.57 ± 2.23 | 8.13 ± 2.38 | -0.2936 | 3.83E-01 | / | G, 0.4590, 0.2621 | / | T, 0.4868, 0.7426 |
| *RFC5* | 3.55 ± 0.48 | 3.19 ± 0.32 | 0.1496 | 3.94E-01 | / | A, 0.1290, 0.0140 | / | / |
| *SORT1* | 23.45 ± 4.65 | 26.76 ± 6.45 | -0.1862 | 4.31E-01 | / | T, 0.0484, 0.0060 | / | T, 0.0790, 0.0000 |
| *RUVBL2* | 31.92 ± 3.81 | 35.26 ± 5.86 | -0.1415 | 4.32E-01 | Fst_intersection | T, 0.0873, 0.0180 | / | / |
| *LDLRAD3* | 2.31 ± 0.63 | 1.96 ± 0.40 | 0.2282 | 4.51E-01 | Fst_intersection | / | / | / |
| *THOC2* | 10.04 ± 0.88 | 9.33 ± 1.03 | 0.1051 | 4.62E-01 | / | / | G, 0.4968, 0.6038 | / |
| *PAFAH1B3* | 1.10 ± 0.84 | 2.79 ± 2.02 | -0.5950 | 4.69E-01 | Fst_intersection | / | / | / |
| *HMMR* | 3.28 ± 0.96 | 3.84 ± 0.93 | -0.2201 | 4.86E-01 | / | A, 0.2750, 0.5204 | / | A, 0.1667, 0.5263 |
| *MLL* | 19.28 ± 2.6 | 21.17 ± 4.11 | -0.1328 | 5.13E-01 | / | C, 0.1557, 0.0783 | / | / |
| *ZNF428* | 5.72 ± 1.43 | 6.44 ± 0.82 | -0.1659 | 5.48E-01 | Fst_intersection | / | / | / |
| *GNA12* | 4.51 ± 0.37 | 4.25 ± 0.46 | 0.0850 | 5.57E-01 | / | C, 0.1032, 0.0140 | / | / |
| *PHLDB3* | 5.26 ± 1.12 | 5.87 ± 1.19 | -0.1547 | 5.60E-01 | Fst_intersection | / | / | / |
| *LYPD3* | 0.32 ± 0.17 | 0.22 ± 0.16 | 0.4103 | 5.65E-01 | Fst_intersection | / | / | / |
| *PPM1B* | 11.77 ± 2.05 | 12.72 ± 0.99 | -0.1110 | 5.95E-01 | / | / | A, 0.2138, 0.3365 | / |
| *MAG* | 0.36 ± 0.20 | 0.50 ± 0.13 | -0.3621 | 6.00E-01 | Fst_intersection | / | / | / |
| *GNAI3* | 20.37 ± 2.30 | 21.50 ± 1.23 | -0.0772 | 6.20E-01 | / | / | / | C, 0.1410, 0.0000 |
| *SUMO1* | 26.03 ± 7.26 | 28.81 ± 2.89 | -0.1426 | 6.20E-01 | / | / | G, 0.4628, 0.5651 | G, 0.3421, 0.5605 |
| *CCDC97* | 5.45 ± 0.99 | 5.89 ± 0.63 | -0.1103 | 6.38E-01 | Fst_intersection | / | / | / |
| *PAF1* | 15.77 ± 1.42 | 15.11 ± 0.86 | 0.0612 | 6.60E-01 | Fst_intersection | / | / | / |
| *GNAT2* | 0.13 ± 0.21 | 0.06 ± 0.08 | 0.3736 | 6.71E-01 | / | / | / | C, 0.1410, 0.0000 |
| *PRKG2* | 0.81 ± 0.24 | 0.94 ± 0.46 | -0.2005 | 7.01E-01 | / | / | / | G, 0.4868, 0.7904 |
| *TP63* | 0.85 ± 0.63 | 0.72 ± 0.15 | 0.2125 | 7.20E-01 | / | / | / | C, 0.1410, 0.0074 |
| *MECP2* | 7.54 ± 0.63 | 7.25 ± 0.72 | 0.0552 | 7.21E-01 | / | G, 0.0635, 0.0080 | / | G, 0.0513, 0.0000 |
| *IRGQ* | 3.88 ± 0.47 | 3.68 ± 0.82 | 0.0782 | 7.42E-01 | Fst_intersection | / | / | / |
| *DEDD2* | 8.45 ± 1.14 | 8.87 ± 1.05 | -0.0675 | 7.43E-01 | Fst_intersection | / | / | / |
| *EIF3M* | 24.45 ± 3.30 | 23.4 ± 2.84 | 0.0628 | 7.47E-01 | / | A, 0.1190, 0.0101 | / | A, 0.1667, 0.0074 |
| *SOBP* | 0.86 ± 0.24 | 0.92 ± 0.17 | -0.0897 | 8.25E-01 | Fst_intersection | / | / | / |
| *ITPKC* | 4.05 ± 0.80 | 4.23 ± 0.77 | -0.0626 | 8.26E-01 | Fst_intersection | / | / | / |
| *NCAPG* | 1.12 ± 0.26 | 1.17 ± 0.18 | -0.0578 | 8.49E-01 | / | C, 0.1210, 0.01235 | / | / |
| *NUMBL* | 1.88 ± 0.17 | 1.82 ± 0.32 | 0.0405 | 8.64E-01 | Fst_intersection | / | / | / |
| *RAB4B* | 8.11 ± 1.13 | 8.34 ± 1.34 | -0.0397 | 8.65E-01 | Fst_intersection | / | / | / |
| *LRFN3* | 0.62 ± 0.44 | 0.68 ± 0.08 | -0.1055 | 8.81E-01 | / | / | / | A, 0.1410, 0.0110 |
| *PARG* | 7.12 ± 0.90 | 7.27 ± 0.84 | -0.0289 | 8.95E-01 | Fst_intersection | / | / | / |
| *SNRNP70* | 121.24 ± 28.06 | 124.62 ± 17.96 | -0.0387 | 8.95E-01 | / | T, 0.0873, 0.0160 | / | T, 0.1410, 0.0110 |
| *PAICS* | 10.16 ± 2.17 | 9.91 ± 1.94 | 0.0347 | 9.08E-01 | / | / | T, 0.1667, 0.2759 | / |
| *NTF4* | 1.15 ± 0.44 | 1.21 ± 0.46 | -0.0699 | 9.08E-01 | Fst_intersection | / | / | / |
| *ADCK4* | 0.25 ± 0.19 | 0.22 ± 0.16 | 0.1007 | 9.13E-01 | Fst_intersection | / | / | / |
| *MED14* | 7.51 ± 0.91 | 7.64 ± 1.42 | -0.0258 | 9.14E-01 | / | / | G, 0.4267, 0.5757 | / |
| *GRIK5* | 0.39 ± 0.15 | 0.37 ± 0.17 | 0.0584 | 9.22E-01 | Fst_intersection | / | / | / |
| *ARRB2* | 55.51 ± 10.82 | 56.41 ± 15.45 | -0.0227 | 9.42E-01 | / | / | / | C, 0.4359, 0.6805 |
| *DEPDC7* | 1.81 ± 0.82 | 1.86 ± 0.32 | -0.0344 | 9.50E-01 | / | C, 0.0807, 0.0125 | / | C, 0.1316, 0.0078 |
| *PRR19* | 2.09 ± 0.66 | 2.06 ± 0.82 | 0.0247 | 9.58E-01 | Fst_intersection | / | / | / |
| *AIFM3* | 0.06 ± 0.06 | 0.06 ± 0.03 | -0.0537 | 9.58E-01 | / | / | A, 0.0828, 0.1709 | / |
| *CYP2A6* | 0.52 ± 0.29 | 0.54 ± 0.28 | -0.0370 | 9.63E-01 | Fst_intersection | G, 0.0794, 0.0120 | G, 0.0422, 0.0094 | G, 0.1282, 0.0110 |
| *MLH1* | 5.77 ± 0.41 | 5.74 ± 0.90 | 0.0075 | 9.74E-01 | / | G, 0.2661, 0.1446 | / | G, 0.3816, 0.1593 |
| *LRFN1* | 0.24 ± 0.16 | 0.23 ± 0.08 | 0.0240 | 9.78E-01 | Fst_intersection | / | / | / |
| *EID2* | 2.15 ± 0.69 | 2.17 ± 0.75 | -0.0124 | 9.81E-01 | Fst_intersection | / | / | / |
| *RPS16* | 1.46 ± 0.26 | 1.45 ± 0.45 | 0.0063 | 9.88E-01 | Fst_intersection | / | G, 0.0458, 0.0095 | / |
| *EGLN2* | 1.08 ± 0.44 | 1.08 ± 0.19 | 0.0023 | 9.96E-01 | Fst_intersection | / | / | / |
| *LASS3* | 0.00 ± 0.00 | 0.00 ± 0.00 | NA | NA | Fst_intersection | / | / | / |
| *DLL3* | 0.00 ± 0.01 | 0.00 ± 0.00 | 0.0069 | NA | Fst_intersection | / | / | / |
| *FSTL5* | 0.00 ± 0.00 | 0.00 ± 0.00 | NA | NA | Fst_intersection | / | / | / |
| *MIA* | 0.00 ± 0.00 | 0.00 ± 0.00 | NA | NA | Fst_intersection | / | / | / |
| *APOC2* | 0.00 ± 0.00 | 0.02 ± 0.05 | -0.0869 | NA | Fst_intersection | / | / | / |
| *APOC4* | 0.00 ± 0.00 | 0.00 ± 0.00 | NA | NA | Fst_intersection | / | / | / |
| *TEX101* | 0.00 ± 0.00 | 0.00 ± 0.00 | NA | NA | Fst_intersection | / | / | / |
| *IL28A* | 0.00 ± 0.00 | 0.02 ± 0.03 | -0.1472 | NA | Fst_intersection | / | / | / |
| *MED29* | 0.00 ± 0.01 | 0.01 ± 0.02 | -0.0534 | NA | Fst_intersection | / | / | / |
| *FFAR1* | 0.06 ± 0.08 | 0.09 ± 0.10 | -0.1990 | NA | Fst_intersection | / | / | / |
| *RAG2* | 0.00 ± 0.00 | 0.00 ± 0.00 | NA | NA | Fst_intersection | / | / | / |
| *GAPDHS* | 0.00 ± 0.00 | 0.03 ± 0.05 | -0.2075 | NA | Fst_intersection | / | T, 0.0520, 0.0094 | / |
| *NR5A1* | 0.00 ± 0.01 | 0.00 ± 0.00 | 0.0069 | NA | / | / | G, 0.4412, 0.5791 | / |
| *ACTRT1* | 0.00 ± 0.00 | 0.00 ± 0.00 | NA | NA | / | / | T, 0.1233, 0.0692 | / |
| *CRX* | 0.00 ± 0.00 | 0.00 ± 0.00 | NA | NA | / | / | C, 0.0422, 0.0094 | / |
| *FSHB* | 0.00 ± 0.00 | 0.00 ± 0.00 | NA | NA | / | / | / | G, 0.1029, 0.0037 |
| *CYP2S1* | #N/A^i^ | #N/A | #N/A | #N/A | Fst_intersection | / | / | / |
| *CDK19* | #N/A | #N/A | #N/A | #N/A | Fst_intersection | / | / | / |
| *SHKBP1* | #N/A | #N/A | #N/A | #N/A | Fst_intersection | / | / | / |
| *SRRM5* | #N/A | #N/A | #N/A | #N/A | Fst_intersection | / | / | / |
| *APOC1* | #N/A | #N/A | #N/A | #N/A | Fst_intersection | A, 0.0794, 0.0120 | / | A, 0.1282, 0.0110 |
| *CYP2A13* | #N/A | #N/A | #N/A | #N/A | Fst_intersection | / | / | / |
| *CYP2A7* | #N/A | #N/A | #N/A | #N/A | Fst_intersection | / | / | / |
| *IL29* | #N/A | #N/A | #N/A | #N/A | Fst_intersection | / | / | / |
| *CGB1* | #N/A | #N/A | #N/A | #N/A | Fst_intersection | / | / | / |
| *CGB2* | #N/A | #N/A | #N/A | #N/A | Fst_intersection | / | / | / |
| *CGB* | #N/A | #N/A | #N/A | #N/A | Fst_intersection | / | / | / |
| *CGB5* | #N/A | #N/A | #N/A | #N/A | Fst_intersection | / | / | / |
| *CGB8* | #N/A | #N/A | #N/A | #N/A | Fst_intersection | / | / | / |
| *CGB7* | #N/A | #N/A | #N/A | #N/A | Fst_intersection | / | / | / |
| *LGALS14* | #N/A | #N/A | #N/A | #N/A | Fst_intersection | / | / | / |
| *CLC* | #N/A | #N/A | #N/A | #N/A | Fst_intersection | / | / | / |
| *POU2F2* | #N/A | #N/A | #N/A | #N/A | Fst_intersection | / | / | / |
| *RPS16P10* | #N/A | #N/A | #N/A | #N/A | Fst_intersection | / | / | / |
| *RPS16P1* | #N/A | #N/A | #N/A | #N/A | Fst_intersection | / | / | / |
| *FTLP2* | #N/A | #N/A | #N/A | #N/A | Fst_intersection | / | / | / |
| *EID2B* | #N/A | #N/A | #N/A | #N/A | Fst_intersection | / | / | / |
| *CSTF3* | #N/A | #N/A | #N/A | #N/A | / | C, 0.0807, 0.0125 | / | C, 0.1316, 0.0078 |
| *H2AFB1* | #N/A | #N/A | #N/A | #N/A | / | A, 0.0952, 0.0120 | G, 0.0525, 0.0157 | / |
| *CXCL1* | #N/A | #N/A | #N/A | #N/A | / | C, 0.1429, 0.0340 | / | C, 0.1795, 0.0257 |
| *LGALS4* | #N/A | #N/A | #N/A | #N/A | / | / | / | T, 0.1410, 0.0110 |
| *AXL* | #N/A | #N/A | #N/A | #N/A | / | / | / | A, 0.1282, 0.0074 |

^a^ Genes expression (normalized gene counts) in healthy lung tissues derived from RNA_seq.

^b^ Genes expression (normalized gene counts) in affected lung lesions derived from RNA_seq.

^c^ Log2(fold change).

^d^ P values adjusted by Benjamini-Hochberg adjustment.

^e^ Minor allele.

^f^ The frequency of minor allele in the disease-resistant group.

^g^ The frequency of minor allele in the disease-susceptible group.

^h^ Whether it is in 107 common genes derived from the intersection of the top 1% F_ST_ loci of three different grouping methods.

^i^ #N/A means genes not found in RNA_seq results.
